# Knockout of the PKN Family of Rho Effector Kinases Reveals a Non-redundant Role for PKN2 in Developmental Mesoderm Expansion

**DOI:** 10.1016/j.celrep.2015.12.049

**Published:** 2016-01-07

**Authors:** Ivan Quétier, Jacqueline J.T. Marshall, Bradley Spencer-Dene, Sylvie Lachmann, Adele Casamassima, Claudio Franco, Sarah Escuin, Joseph T. Worrall, Priththivika Baskaran, Vinothini Rajeeve, Michael Howell, Andrew J. Copp, Gordon Stamp, Ian Rosewell, Pedro Cutillas, Holger Gerhardt, Peter J. Parker, Angus J.M. Cameron

**Affiliations:** 1 Kinase Biology Laboratory, John Vane Science Centre, Barts Cancer Institute, Queen Mary University of London, Charterhouse Square, London EC1M 6BQ, UK; 2 Protein Phosphorylation Laboratory, Francis Crick Institute, 44 Lincoln’s Inn Fields, London WC2A 3LY, UK; 3 Francis Crick Institute, 44 Lincoln’s Inn Fields, London WC2A 3LY, UK; 4 Instituto Medicina Molecular (iMM), Faculdade de Medicina da Universidade de Lisboa, 1649-028 Lisbon, Portugal; 5 Newlife Birth Defects Research Centre, Institute of Child Health, University College, London WC1N 1EH, UK; 6 John Vane Science Centre, Barts Cancer Institute, Queen Mary University of London, Charterhouse Square, London EC1M 6BQ, UK; 7 Genetic Manipulation Services, Francis Crick Institute, Clare Hall, Herts EN6 3LD, UK; 8 Max-Delbrück-Center for Molecular Medicine, Robert-Rössle-Strasse 10, 13125 Berlin, Germany; 9 Division of Cancer Studies, King’s College London, New Hunt’s House, Saint Thomas Street, London SE1 1UL, UK

**Keywords:** PKC, protein kinase C, PKN, Protein kinase N, MEF, mouse embryonic fibroblasts, ES cells, Embryonic stem cells, 4-OHT, 4-hydroxytamoxifen, NCCs, neural crest cells

## Abstract

In animals, the protein kinase C (PKC) family has expanded into diversely regulated subgroups, including the Rho family-responsive PKN kinases. Here, we describe knockouts of all three mouse PKN isoforms and reveal that PKN2 loss results in lethality at embryonic day 10 (E10), with associated cardiovascular and morphogenetic defects. The cardiovascular phenotype was not recapitulated by conditional deletion of PKN2 in endothelial cells or the developing heart. In contrast, inducible systemic deletion of PKN2 after E7 provoked collapse of the embryonic mesoderm. Furthermore, mouse embryonic fibroblasts, which arise from the embryonic mesoderm, depend on PKN2 for proliferation and motility. These cellular defects are reflected in vivo as dependence on PKN2 for mesoderm proliferation and neural crest migration. We conclude that failure of the mesoderm to expand in the absence of PKN2 compromises cardiovascular integrity and development, resulting in lethality.

## Highlights

• PKN2, but not PKN1 or PKN3, is essential during mouse embryogenesis
• PKN2 knockout causes severe cardiovascular and morphogenetic abnormalities
• PKN2 is required for mesenchymal growth and neural crest migration in vivo

## Graphical Abstract

**Figure:**
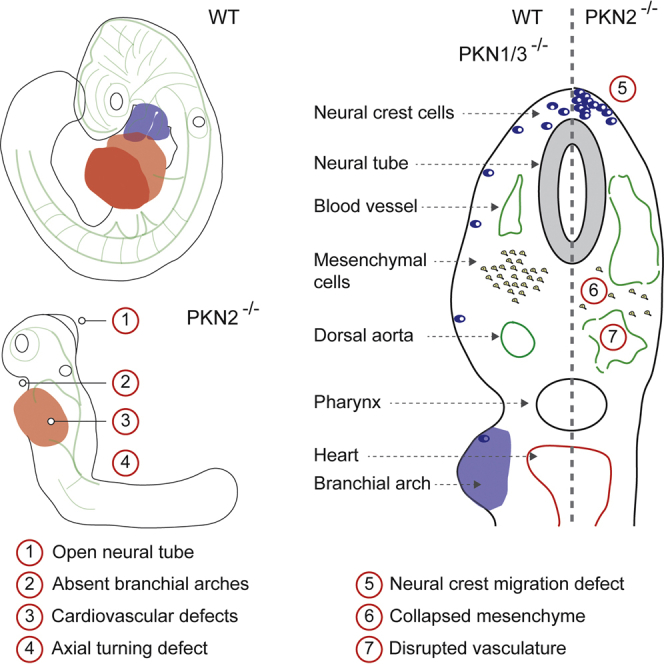

## Teaser

The Rho effector PKN kinases regulate diverse cellular functions, but their in vivo function is unexplored. By systematically targeting the PKN family, Quetier et al. reveal a unique role for PKN2 during developmental growth and morphogenesis. These findings impact on developmental disorders and the targeting of PKN in disease.

## Introduction

The Rho family guanosine triphosphatases (GTPases), which includes Rho, Rac, and Cdc42, are regulators of cell shape, adhesion, and motility and as such are critical in development (Bustelo et al., 2007). Numerous studies have examined the roles of Rho family members at a biochemical and cellular level; however, their ubiquitous expression and pleiotropic roles have made elucidation of in vivo function difficult. This has in part been addressed using tissue-specific conditional alleles of Rho family members (D’Amico et al., 2009). To gain a deeper insight, it is necessary to examine the roles of specific effector pathways. Among the diverse array of effectors are many Ser/Thr kinases, which include the Rho family-activated kinases (ROCK1/2), p21-activated kinases (PAK1–PAK6), and protein kinase N (PKN) kinases (Zhao and Manser, 2005).

The PKN kinases represent a subfamily of the protein kinase C (PKC) family, sharing a high degree of homology within their C-terminal kinase domains (Mukai, 2003). The divergent N-terminal regulatory domains bear a C2 domain and three polybasic coiled-coil HR1 domains, which confer binding and regulation by Rho family members. While other mammalian PKCs lack HR1 domains and Rho responsiveness, PKC orthologs in yeast bear two HR1 domains and signal downstream of Rho1; yeast PKC also retains the regulatory C1 and C2 domains found in mammalian PKC isoforms, suggesting an evolutionary split between the Rho-responsive and the Rho-independent functions of the PKC family. There are three PKN family members in mammals (PKN1–PKN3, also known as PRKs), which vary in their regulation. While both PKN1 and PKN2 are activated by RhoA and Rac1 (Flynn et al., 1998, Vincent and Settleman, 1997), they respond differently to phosphoinositides and fatty acids (Mukai, 2003). PKN isoforms also respond to distinct stimuli and bind to distinct effectors, highlighting functional divergence within the family. Expression patterns also differ; PKN1 and PKN2 mRNAs are ubiquitous in expression, while PKN3 is more restricted to muscle, liver, and endothelial cells (Aleku et al., 2008, Palmer et al., 1995). In vivo, a role for PKN1 in germinal center formation has been reported (Yasui et al., 2012).

Here, we report genetic ablation of all three mouse PKN isoforms to reveal a non-redundant essential role for PKN2 in the developing embryonic mesenchyme.

## Results

### PKN2 Plays a Non-redundant Role in Development

The mouse PKN1 and PKN3 genes were targeted in embryonic stem cells (ESCs) by homologous recombination (Figure S1), and germline transmission resulted in fertile heterozygous mice. Gene disruption was assessed by RT-PCR and western blot (Figures S1D–S1G). PKN1 and PKN3 crosses generated Mendelian ratios of wild-type (WT), heterozygous, and knockout (KO) mice (Table S1). Mice were fertile and exhibited no overt phenotype. To assess redundancy, the PKN1 and PKN3 mice were interbred, which generated Mendelian numbers of healthy, fertile, double-KO mice (Table S1).

PKN2-targeted “KO-first” ESCs were obtained from the Knockout Mouse Project (KOMP) consortium (Skarnes et al., 2011). The KO-first strategy generates a null allele (PKN2^tm1a^) but allows conversion to a conditional PKN2^flox^ allele through flp/FRT recombination (PKN2^tm1c^; Figure S1). Germline transmission was achieved with two independent ESC clones, but crossing PKN2^tm1a^ heterozygotes (herein referred to as PKN2^+/−^) from either ESC clone generated no homozygous PKN2 KO mice (Table S1). To confirm that this results from disruption of the PKN2 gene, the locus was flp/FRT recombined (PKN2^tm1c^) by crossing with a Flp deleter mouse; recombination rescued expression of PKN2 and survival of homozygous offspring (Table S1). Finally, PKN2^+/−^ mice crossed onto a PKN1/3 KO background were generated at the expected frequency with no overt phenotype (n = 10), indicating that a single allele of PKN2 is sufficient for embryogenesis.

### PKN2 Loss Results in Embryonic Lethality by E10

**Figure 1:**
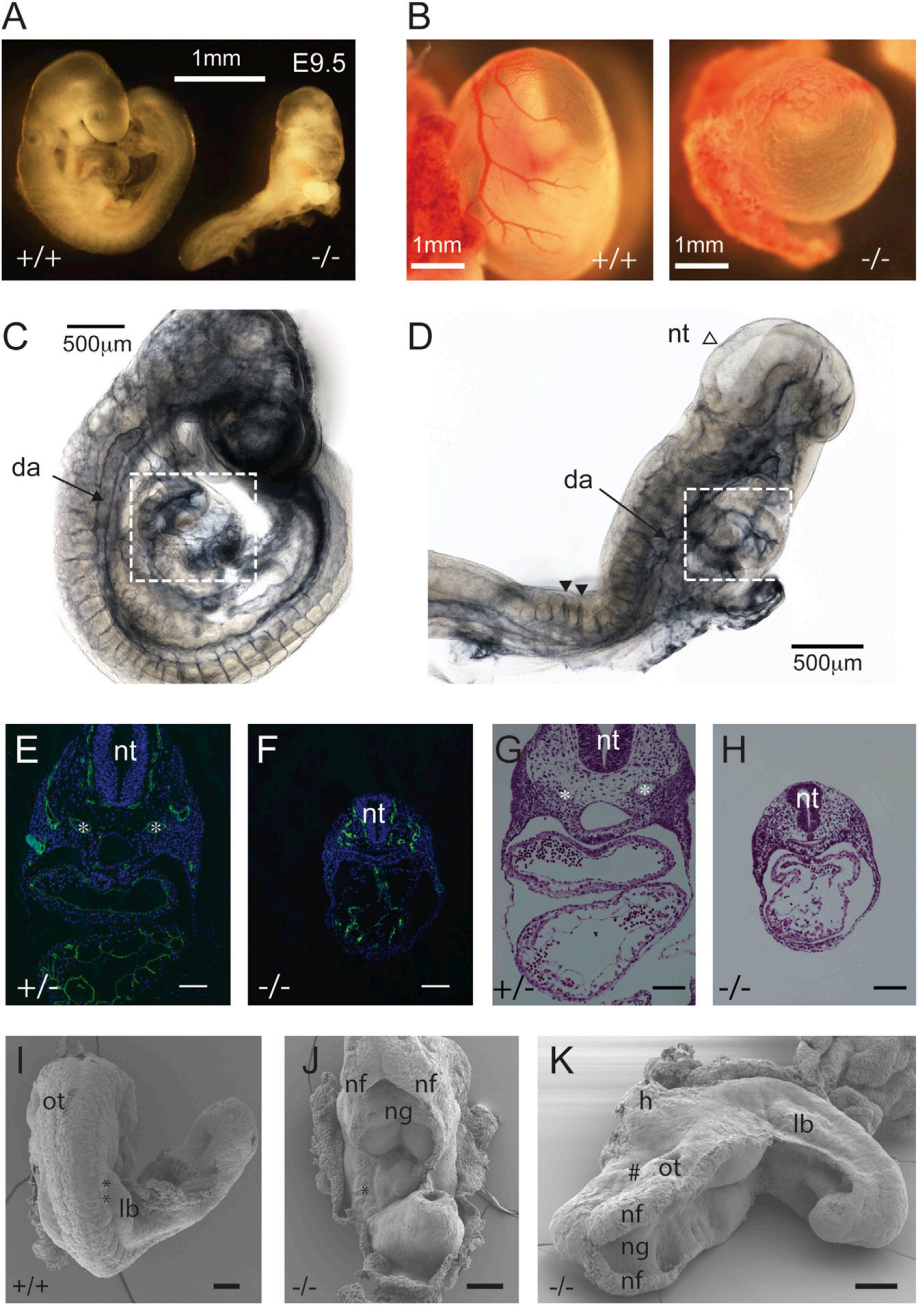
PKN2 Ablation Is Embryonic Lethal and Causes Growth, Morphogenetic, and Cardiovascular Defects (A) PKN2^−/−^ embryos are smaller than littermates, do not undergo axial turning, and exhibit no branchial arches. (B) Yolk sacs from mutant embryos have no vitelline vessels and exhibit an immature vascular plexus. (C and D) Whole-mount PECAM1 staining of E9.5 PKN2^+/−^ and PKN2^−/−^ embryos. KO embryos exhibit collapsed dorsal aorta and underdeveloped heart (boxed). The open neural tube example (open arrowhead) and intersomitic vessel examples (closed arrowheads) are indicated. (E–H) Transverse sections through E9.5 embryos were subjected to endomucin staining (E and F) or H&E (G and H). Asterisks indicate dorsal aorta in control PKN2^+/−^ embryos. (I–K) Scanning electron microscopy of WT (+/+) and PKN2^−/−^ E9.5 embryos indicating fully open neural tube in PKN2^−/−^ embryos. Asterisks indicate somites; hash mark indicates under-developed head. Da, dorsal aorta; nt, neural tube; ng, neural groove; nf, neural fold; ot, otic vesicle; lb, limb bud. Scale bars represent 100 μm unless stated.

Homozygous embryos derived from both independent ESC clones died at embryonic day 10 (E10; Table S1) and failed to undergo axial turning (Figure 1A). No PKN2 protein or truncated fragments were detectable in KO E8 embryos or yolk sacs, while PKN2^+/−^ embryos expressed half as much PKN2, confirming gene disruption (Figures S2A and S2B). Compressed somites (14–18 somite pairs) in E9.5 mutant embryos demonstrate a failure to elongate, consistent with defects in convergent extension. A lack of branchial arch formation and head development implied a lack of mesenchymal expansion (Figures 1A and 1K). The absence of vitelline vessels on the yolk sacs and pericardial edema indicated a cardiovascular or angiogenic defect (Figure 1B), and the neural tube remained open to varying degrees in all embryos (Figures 1D, 1J, and 1K). To exclude trophoblast or placental defects as the cause of lethality, we conducted tetraploid rescue experiments, which indicated that PKN2 is required in the embryo or yolk sac (Figure S2C).

Immunostaining indicated ubiquitous expression of PKN2 in E10 embryo frozen sections, with some accumulation at the apical surface of the neuroepithelium and in the ectoderm (Figure S2D). LacZ reporter expression (β-galactosidase) confirmed broad PKN2 expression (Figure S2E). qRT-PCR analysis of PKN1 and PKN3 expression levels in E8 embryos indicated no mRNA expression compensation in the PKN2 KOs (Figure S2F). PKN1 and PKN2 mRNAs were both readily detectable by RNAscope broadly throughout the embryo. While PKN3 protein could not be detected in embryo extracts, mRNA could be detected in endothelial cells (as previously reported) and at low levels in other embryo tissues (Figure S2G).

### PKN2 Null Embryos Have Cardiovascular and Neural Tube Defects

Immunostaining and histology of yolk sacs revealed an immature vascular plexus (Figures S3A and S3B) and separation of the endodermal and mesodermal layers in PKN2 KOs (Figure S3A). These phenotypes are associated with both cardiac and angiogenic defects. Heart and vascular development are intrinsically linked processes, because hemodynamic force is necessary for vascular remodeling (Lucitti et al., 2007). Substantial numbers of primitive nucleated blood cells within the vasculature of PKN2 KO embryos, and beating hearts, indicated that circulation is established, typically occurring at E8.5–E9. Whole-mount staining of the vasculature indicated collapsed major vessels and sparse peripheral vasculature (Figures 1C and 1D). This was confirmed in transverse sections, where a disorganized endothelial network is apparent throughout the mesenchyme (Figures 1E and 1F). Histology also revealed a delay in cardiac looping, more typical of an embryonic heart at E8 (Figures 1G and 1H; Figure S3), further confirmed by staining E8.5 whole-mount and transverse sections of embryo hearts for desmin; 3D reconstructions revealed a delay in heart morphogenesis (Figures S3C and S3D).

PKN2 KO embryos exhibit a range of neural tube defects. Some prospective forebrain development in E9.5 embryos was observed, with closure of the forebrain neural fold to form a neural lumen, diencephalon, and optic vesicles (Figure S3E). The prospective hind-brain neural tube was always open, often with no discernible neural groove. Some embryos exhibit chranioraschischisis (Figures 1I–1K), a phenotype associated with planar cell polarity (PCP) mutants, such as looptail (Murdoch et al., 2001), which fail to undergo convergent extension.

### Conditional PKN2^flox^ Deletion in Either the Endothelium or the Heart Does Not Phenocopy the Embryonic Vascular Defect

**Figure 2:**
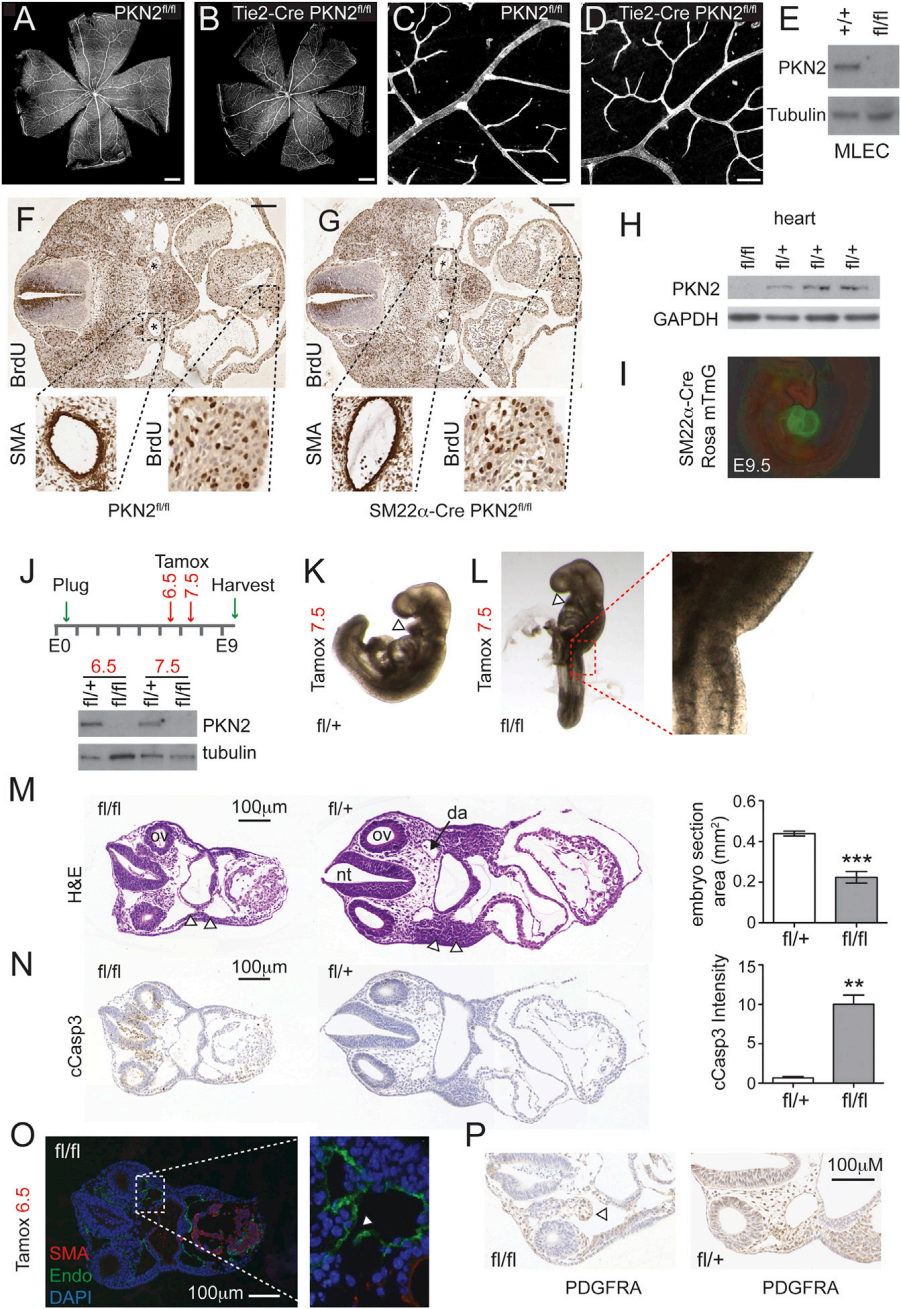
Inducible Deletion at E6.5, but Not Tie2-Cre or SM22α-Cre, Phenocopies the Constitutive Knockout (A–D) Retinal vasculature was stained for isolectin B. Scale bars represent 500 μm (A and B) and 50 μm (C and D). (E) Western blots indicate that endothelial PKN2 is deleted in Tie2-Cre PKN2^fl/fl^ mice. (F and G) Transverse sections through E11.5 embryos reveal normal heart and major vessel development in SM22α-Cre PKN2^fl/fl^ embryos. Scale bars represent 200 μm. Heart ventricle proliferation (BrdU) and SMA staining of the dorsal aorta (^∗^) were comparable. (H) Western blots indicate PKN2 deletion in adult heart tissue from SM22α-Cre PKN2^fl/fl^ mice. (I) SM22α-Cre recombination in hearts is robust at E9.5, as assessed by the Rosa mTmG reporter. (J) Tamoxifen was given on day 6 or 7 of pregnancy before embryo harvest at E9.5; PKN2 protein was lost in PKN2^fl/fl^ embryos at E9.5 (bottom panels). (K and L) Deletion of PKN2 at E7.5 induced axial turning defects in iCre PKN2^fl/fl^ embryos. (M and N) Transverse sections from E9.5 embryos following E6.5 deletion were stained for H&E and cleaved caspase 3. Littermate embryo cross sectional area and caspase 3 pixel intensity were measured using panoramic viewer software; data are mean ± SD (n = 3). Open arrowheads indicate neural crest-derived branchial arches, absent following PKN2 deletion (M). Sections show the otic vesicles (ov), neural tube (nt), and dorsal aorta (da). Brightfield slide images were acquired on a Pannoramic 250 slide scanner (3Dhistech) and may comprise multiple instrument merged panels. (O) Endomucin and SMA staining revealed collapsing vessels (arrowhead) and an unlooped heart. (P) PDGFRA staining confirmed mesenchymal regression.

To investigate an endothelial role, we crossed our conditional PKN2^flox^ mouse (PKN2^tm1c^; Figure S1) with the endothelial-specific Tie2-Cre (Koni et al., 2001). Tie2-Cre has been used to uncover embryonic endothelial dependencies displaying phenotypes similar to our PKN2 KO, including phosphatidylinositol 3-kinase p110α (Graupera et al., 2008). In contrast to global PKN2 KO, Tie2-Cre PKN2^fl/fl^ mice were born at Mendelian frequency and displayed no overt phenotype (Table S2). An analysis of adult mouse retinal vasculature also revealed no discernible angiogenic differences in Tie2-Cre PKN2^fl/fl^ mice (Figures 2A–2D). Deletion was confirmed by immunoblot of mouse lung endothelial cell lysates (Figure 2E). We conclude that PKN2 is largely dispensable in endothelial cells. Although Tie2 is expressed in all endothelial cells (Schlaeger et al., 1997), a role for PKN2 in endothelial precursors before Tie2-Cre recombination cannot be entirely excluded.

Mutants with severe cardiac defects, such as the contraction-deficient MLC2a deletion (Huang et al., 2003) exhibit embryonic vascular defects and die mid-gestation, indicating the need for hemodynamic force in vascular remodeling (Lucitti et al., 2007). To delete PKN2 in the embryonic heart, we crossed PKN2^flox^ mice with SM22α-Cre, which is expressed in the developing heart tube (from E7.5) and smooth muscle cells (Lepore et al., 2005). SM22α-Cre-mediated PKN2 deletion shows a partially penetrant lethal phenotype (Table S2). Surviving mice were fertile, but pathology revealed cardiac hypertrophy, lung fibrosis, and alveolar enlargement. To examine effects on heart development, we collected several sets of embryos (E9.5–E13.5). Embryo numbers were Mendelian, and KOs were indistinguishable from littermates (Table S2). Transverse sections revealed no gross differences in heart size or cardiomyocyte bromodeoxyuridine (BrdU) incorporation (Figures 2F and 2G). Smooth muscle actin (SMA) staining also demonstrated comparable smooth muscle staining of the dorsal aorta. Recombination was confirmed by loss of PKN2 protein in adult heart ventricle extracts (Figure 2H), and robust Cre expression throughout the E9.5 embryo heart was confirmed by crossing with the Rosa mTmG reporter mouse (Figure 2I). Although SM22 partial lethality and adult pathology indicate an unexplored role for PKN2 in the heart or smooth muscle cells, the survival of some offspring, and the near-normal embryonic development, implies that PKN2 is not essential for early cardiovascular function.

### Inducible Deletion of PKN2 In Vivo Results in Mesenchymal Collapse

Next, we examined the temporal requirements for PKN2 by crossing with Rosa26 Cre-ERT2 mice (iCre; Taconic Biosciences), which allows for systemic tamoxifen-induced regulation of Cre activity in vivo. We crossed homozygous iCre PKN2^fl/fl^ mice with PKN2^fl/+^ mice to generate litters with a 1:1 ratio of iCre (heterozygous) PKN2^fl/fl^:PKN2^fl/+^. Because PKN2^+/−^ embryos are indistinguishable from WT embryos, iCre PKN2^fl/+^ act as controls. A single dose of tamoxifen (3 mg oral gavage) induced loss of PKN2 within 48 hr (Figure 2J). Tamoxifen administration at E7.5 was sufficient to recapitulate the axial turning defects in most (7/10) E9.5 PKN2^fl/fl^ embryos (Figure 2L). Tamoxifen administration at E6.5 results in recapitulation of the gross phenotypes of the full PKN2 KO (Figures 2M–2O). PKN2^fl/fl^ embryos also exhibit collapse of the mesenchyme, apoptosis, vascular disintegration (Figures 2M–2P), and loss of branchial arches (Figures 2K–2M). Together, our conditional KOs support a model in which the heart and vascular defects arise secondary to a deficit within the mesodermal and neural crest-derived mesenchyme.

### PKN2-Deficient MEFs Exhibit Proliferative and Migratory Defects

**Figure 3:**
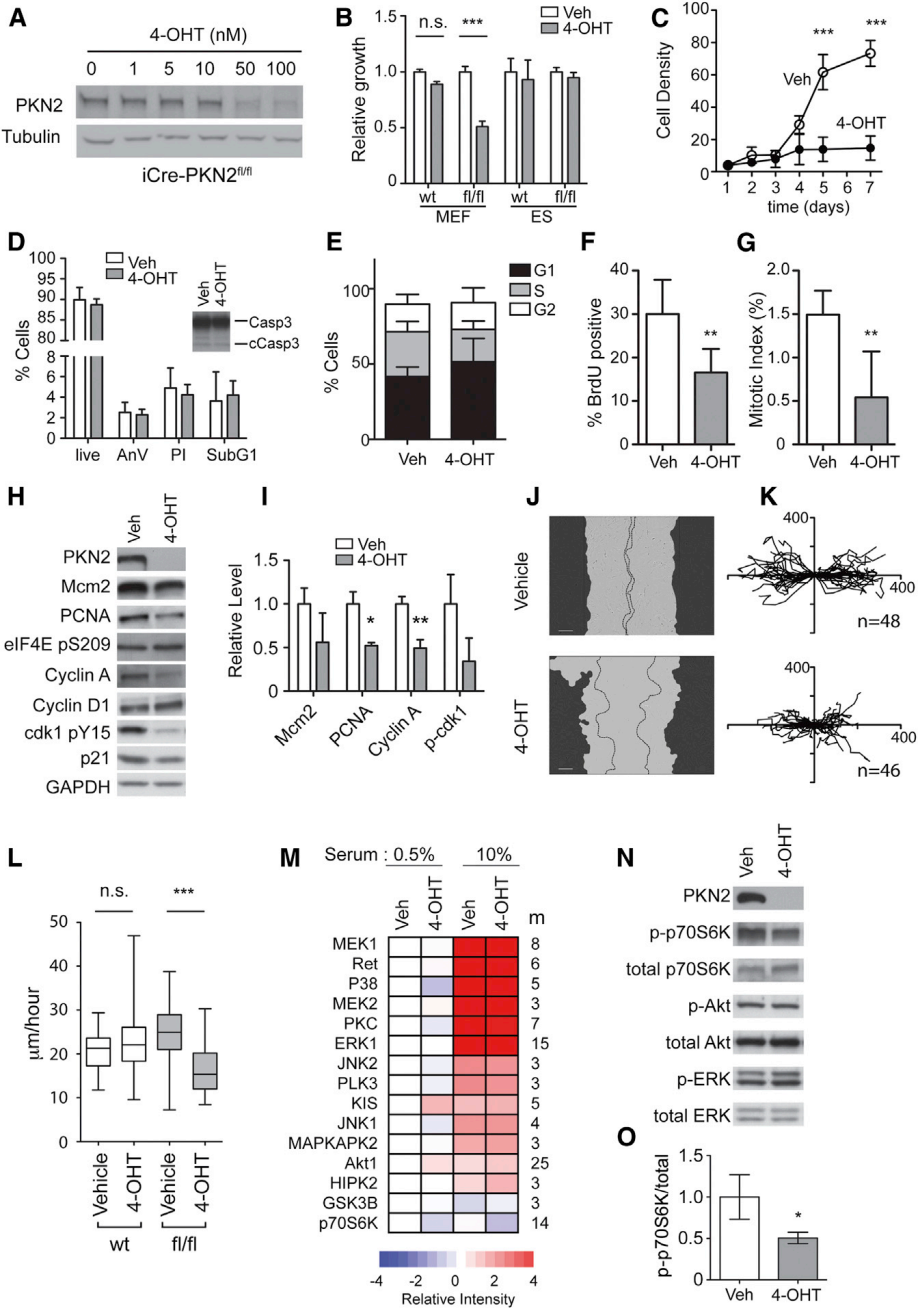
PKN2 Deletion Results in Reduced Proliferation and Migration of MEFs (A) 4-OHT treatment of iCre-PKN2 MEFs results in dose-dependent loss of PKN2. (B and C) 4-OHT induction results in reduced cell viability (B) and slower growth (C) for iCre PKN2^fl/fl^ but not iCre PKN2 WT cells (B; n = 5; ^∗∗∗^p < 0.001). (B) ESC viability is not decreased by PKN2 deletion. (D) No increase in annexin V binding, propidium iodide uptake, sub-G1 cell fraction, or cleaved caspase 3 (cCasp3; inset western blot) indicates no increase in cell death following PKN2 deletion. (E–G) Cell-cycle analysis, BrdU uptake, and p-Histone H3 binding indicate accumulation in the G1/G0 fraction and loss of S-phase and mitotic cells. (H and I) PKN2 deletion caused loss of cell-cycle proteins. Blots were quantified relative to GAPDH; data are mean ± SD (n = 3; p < 0.05, ^∗∗^p < 0.01). (J) 4-OHT Cre induction results in slower scratch wound closure for iCre PKN2^fl/fl^ but not iCre PKN2 WT cells. Mask indicates starting cell front, and dotted lines indicate cell front after 16 hr of migration. (K and L) Single-cell migration speed is reduced; cell tracks (K; axes indicate micrometers) and the speed over the 16 hr time course are displayed (L). Box and whiskers indicate the average, quartiles, and range (^∗∗∗^p < 0.001 ANOVA). (M) Quantitative mass spectrometry (MS) analysis and KSEA indicate broadly comparable serum-induced activation of major signaling pathways following PKN2 deletion. MEFs were serum starved (0.5%) and re-stimulated (10%) for 15 min before analysis by LC-MS/MS (n = 4). Pathway activation (m = number of kinase substrate peptides quantified) relative to control extracts is presented as a heatmap. (N and O) Akt, ERK, and p70S6K activation levels in control and PKN2-depleted cells were assessed by western blot and quantified by densitometry; data are mean ± SD (n = 3; ^∗^p < 0.05).

It was not possible to derive mouse embryonic fibroblasts (MEFs)—which derive from the embryonic mesenchyme—from PKN2^−/−^ embryos; MEFs failed to proliferate post-extraction in contrast to littermate WT and PKN2^+/−^ controls. To examine the cell biological role of PKN2, we instead derived MEFs from the iCre PKN2^fl/fl^ embryos and sex-matched littermate iCre PKN2^+/+^ controls. iCre PKN2^+/+^ MEFs allow for control for nonspecific toxicity of 4-hydroxytamoxifen (4-OHT) or Cre induction. Treatment of iCre PKN2^fl/fl^ MEFs with 100 nM 4-OHT for 1 hr is sufficient to induce PKN2 recombination, as observed by loss of PKN2 after 48 hr (Figure 3A). PKN2 deletion attenuated MEF growth (Figures 3B and 3C), in line with the failure of PKN2 null MEFs to proliferate. No decrease in cell growth was observed for control iCre PKN2^+/+^ cells, and comparable results were observed for multiple iCre PKN2^fl/fl^ MEF lines. Growth dependence is cell type specific, because deletion of PKN2 protein (data not shown) from iCre ESCs had no effect on growth (Figure 3B), as expected given the normal cellular expansion during early embryogenesis.

PKN2 loss did not result in cell death in MEFs; no increase in annexin V staining, propidium iodide uptake, sub-G1 cells, or cleaved caspase 3 was observed following PKN2 deletion (Figure 3D), and non-proliferating cells remained viable through multiple passages. This suggests that cell death observed in the embryo is likely secondary to the death of the embryo.

PKN2 has been reported to regulate mitotic entry and cytokinesis in HeLa cells (Schmidt et al., 2007). In contrast, deletion of PKN2 in MEFs was associated with accumulation of cells in G1/G0 with an accompanied loss of S-phase and mitotic cells, as assessed by BrdU incorporation, phospho-Histone (p-Histone) H3 staining, and cell-cycle analysis (Figures 3E–3G). Decreased expression levels of cyclin A, PCNA, and Mcm2 were consistently observed, as well as decreased levels of phosphorylated Cdk1 (Figures 3H and 3I).

We reported a specific non-redundant role for PKN2 in cancer cell migration (Lachmann et al., 2011). Comparably, scratch wound closure rate and single-cell migration speed were reduced following PKN2^fl/fl^ deletion, while control iCre PKN2^+/+^ MEF migration was unaffected (Figures 3J–3L). The actin cytoskeleton, however, was largely unchanged by PKN2 deletion (Figure S4A).

To gain an unbiased view of PKN2 signaling, we conducted quantitative phosphoproteomic analysis. MEFs ± PKN2 were serum starved overnight and re-stimulated with serum for 15 min before analysis by liquid chromatography-tandem mass spectrometry (LC-MS/MS). In excess of 6,000 individual phosphopeptides were quantified across four biological replicates. Ontological analysis identified numerous phosphopeptides derived from proteins associated with cell cycle, S phase, and mitosis that were significantly depleted in response to PKN2 loss (Table S3). These findings corroborate cell-cycle withdrawal following PKN2 loss. We also found enrichment for phosphopeptides associated with cytoskeletal organization (Table S4), in line with the decreased cell motility observed here and the known functions of the PKN kinases.

Phosphoproteomic pathway mapping through kinase substrate enrichment analysis (KSEA; Casado et al., 2013, Rajeeve et al., 2014) allows assessment of signal transduction by linking phosphosites with their known kinases to determine pathway activation. KSEA revealed that PKN2 loss is associated with minimal changes in serum-induced activation of proliferative signaling (Figures 3M and 3N). The Akt pathway was marginally elevated under serum-starved conditions following PKN2 deletion, although serum-activated Akt levels were comparable. KSEA identified partial suppression of the p70S6K pathway. These findings were confirmed by immunoblot, where active p70S6K was reduced (Figures 3N and 3O), while active ERK and Akt in serum were unchanged. This indicates that serum growth factor coupling to key proliferative signaling axes remains largely intact upon PKN2 deletion and identifies p70S6K as a potential PKN2 effector pathway.

### PKN2 Loss Results in Reduced Proliferation of the Paraxial Mesoderm and Reduced Neural Crest Migration In Vivo

**Figure 4:**
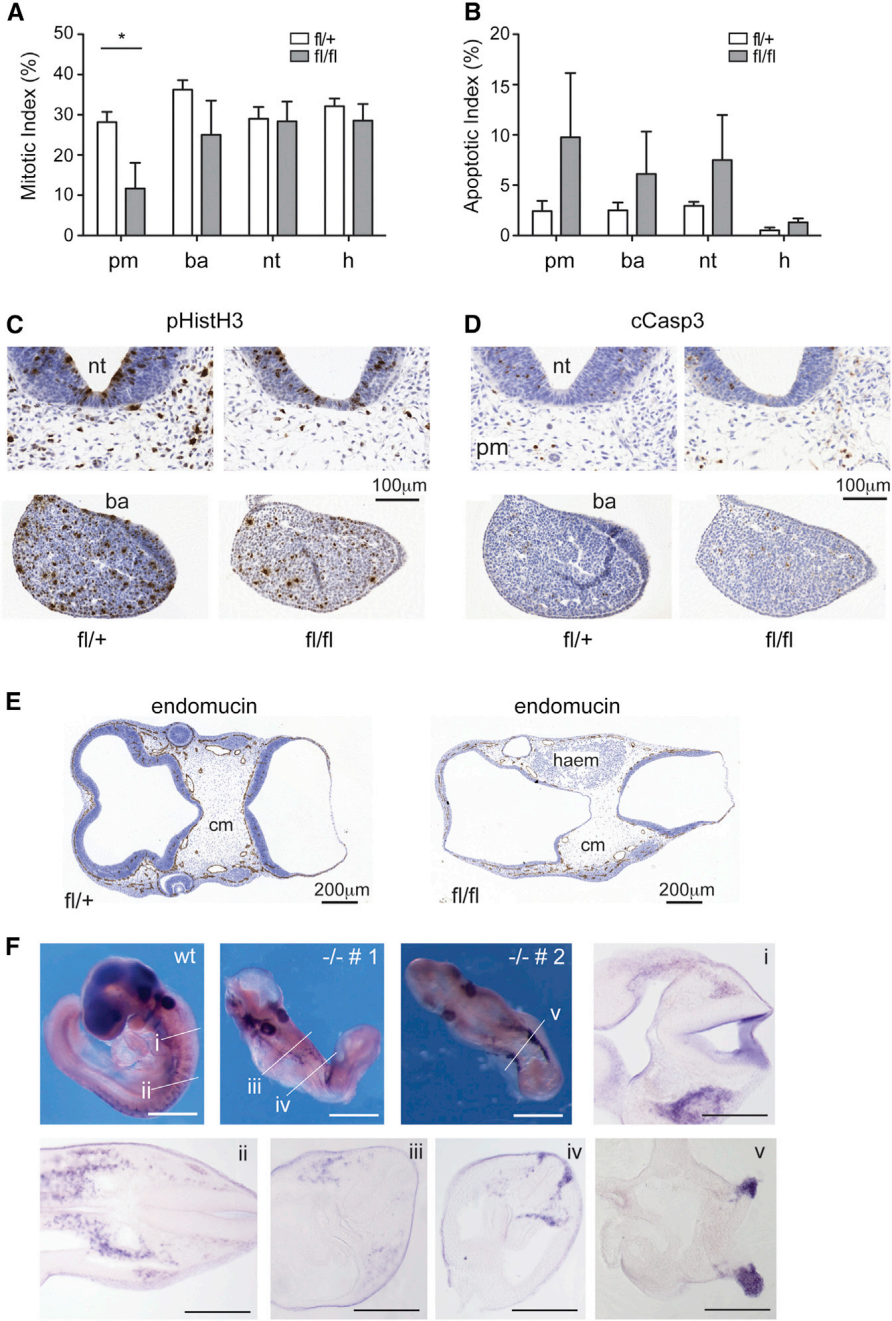
Induced Deletion of PKN2 In Vivo Decreases Mesodermal Proliferation and NCC Migration (A–E) Tamoxifen was given on day 8 of pregnancy before embryo harvest at E10. Embryos were sectioned and stained for p-Histone H3 (pHistH3) to measure mitotic index and cleaved caspase 3 (cCasp3) to measure apoptotic index. (A) Aligned sections were counted to reveal a significant decrease in mitotic index in the pharyngeal mesoderm (pm) but not the branchial arches (ba), neural tube (nt), or heart (h) following PKN2 deletion in PKN2^fl/fl^ but not PKN2^fl/+^ embryos (n = 3; ^∗^p < 0.05). (B) Apoptosis was variably increased across all tissues but did not reach significance. (C and D) Examples of pHistH3 and cCasp3 section stains are displayed. (E) PKN2 loss results in collapse of the cephalic mesoderm (cm) as evidenced by hemorrhage (haem) and loss of cellularity. (F) Whole-mount in situ staining reveals a deficit of migrating NCCs in PKN2 KO embryos. A single-WT and two KO embryos are presented; −/− # 1 has a partially closed neural tube, whereas −/− # 2 exhibits craniorachischisis. Vibratome sections reveal a deficit of NCCs and reduced ventral migration. Scale bars represent 100 μm.

To corroborate the observed mesenchymal cell biology defects, we conducted a series of induced deletions in the iCre PKN2 mouse. Staining of E10 embryos 48 hr after tamoxifen administration allowed quantification of mitosis and apoptosis within distinct tissues. Pharyngeal mesoderm displayed a significant decrease in mitotic cells in PKN2^fl/fl^ embryos when compared with littermate PKN2^fl/+^ controls (Figures 4A and 4C). In contrast, the branchial arch, neural tube, and heart mitotic index remained comparable. Apoptosis in the same tissues was variable between embryos and non-tissue specific (Figures 4B and 4D). These embryos exhibited a deficit of cephalic mesoderm and display evidence of hemorrhage (Figure 4E). Together, these observations support a model in which failure of the mesoderm to proliferate results in loss of support for vascular development and embryo death. This conclusion is reinforced by the extensive apoptosis observed following longer periods of PKN2 deletion (Figure 2N; data not shown).

Global PKN2 KO or induced deletion before E7 results in loss of branchial arches, which derive from both the cranial neural crest and the cranial mesoderm (Noden and Trainor, 2005). Neural crest cells (NCCs) delaminate from the neuroendothelium and migrate ventrally through the mesoderm. We conducted in situ hybridization for erbB3 in whole-mount embryos, which labels migrating NCCs (Britsch et al., 1998). In PKN2 KO embryos, NCCs remain close to where they delaminate instead of migrating ventrally through the mesoderm, as in WT controls. In severe cases, in which embryos exhibit craniorachischisis, NCCs, where detectable, do not migrate (Figure 4F). These data implicate PKN2 in the development and migration of NCCs in vivo.

In summary, our findings support a mechanism by which mesodermal and neural crest lineages acquire dependence on PKN2 for growth and migration mid-gestation. Failure of these lineages to expand and migrate results in loss of the physical support required for cardiovascular integrity and development. The critical contribution of pharyngeal mesoderm and NCCs to the secondary heart field is also likely to contribute to the retarded unlooped hearts in PKN2 null embryos (Plein et al., 2015, Tzahor and Evans, 2011).

## Discussion

PKN kinases are emerging as regulators of cancer growth, invasion, and metastasis. Despite this, and in contrast to other Rho-activated kinases, little is known about their in vivo physiological roles. By targeting the PKN family in vivo, we have uncovered a non-redundant role for PKN2 during development. Here we establish key roles in morphogenesis, lineage-specific growth, migration, and support of cardiovascular development.

Failure to establish a circulatory system in the embryo results in death at mid-gestation (Copp, 1995), the point at which PKN2-null embryos die. Because this process requires interplay among numerous co-dependent systems, unraveling cause and effect can be challenging. Many of the genes identified as essential for establishing circulation are those involved in heart development and endothelial cell function; conditional KOs suggest that PKN2 is largely dispensable here. In contrast, induced loss of PKN2 mid-gestation resulted in collapse of the embryonic paraxial mesoderm, essential for vascular support and heart development (Tzahor and Evans, 2011).

A role for PKN2 in the mesoderm was supported by experiments with mesenchymal-derived MEFs, which depend on PKN2 for growth and motility. In HeLa cells PKN2 regulates mitosis (Schmidt et al., 2007), and in starfish oocytes PKN2 can directly phosphorylate (Ser209), and regulate, the key S-phase initiator, eIF4E (Lee et al., 2000). In contrast, here, in a physiological context, PKN2 loss was associated with arrest in G1/G0, a decrease in S-phase and mitotic cells, and no change in eIF4E phosphorylation (Ser209) or downstream cyclin D1 expression.

PKN2 loss also results in impaired NCC migration in vivo and impaired MEF cell motility in vitro, in concordance with embryo morphogenetic defects. We previously reported a non-redundant role for PKN2 in bladder cancer cell migration (Lachmann et al., 2011), perhaps shedding light on PKN2 isoform specificity. Functionally, the actin cytoskeleton was largely unaffected by PKN2 loss in MEFs, suggesting that RhoA regulation of actin stress fibers is functional. Likewise, F-actin and Rac1 localized independently of PKN2 at the leading edge of migrating cells (Figure S4A). We did, however, identify modulation of many cytoskeletal components in our phosphoproteomics following PKN2 loss (Figure S4), and detailed studies on cytoskeletal dynamics are clearly warranted.

Among the well-described pathways regulating growth and migration in the developing embryo is the lysophosphatidic acid (LPA) axis, where mutants (e.g., autotaxin and Gα12/13) largely phenocopy loss of PKN2, with vascular, neural tube, and axial turning defects (Tanaka et al., 2006, van Meeteren et al., 2006). In support of a role in LPA signaling, deletion of PKN2 from early-passage MEFs was found to blunt LPA-induced migration (Figure S4). However, no differences in LPA-induced actin rearrangement, proliferative signaling, or transcription (CTGF or CYR61) were observed. Thus, while PKN2 may play some role, many aspects of LPA function remain intact.

The Rho GTPases are established regulators of convergent extension and neural tube formation, with focus on the Wnt/PCP pathway (Schlessinger et al., 2009). PCP pathway mutants exhibit craniorachischisis, a severe dorsal neural tube defect (Harris and Juriloff, 1999) also observed in PKN2^−/−^ embryos. Closure of the forebrain neural fold, observed for our PKN2 KO, is further consistent with PCP mutants such as looptail/vangl2 (Copp et al., 1994). Apical accumulation of Rho as a PCP effector has been reported in neural plate morphogenesis in the chick embryo (Kinoshita et al., 2008), and PKN2 is present in this compartment (Figure S2D). Intriguingly, ROCK has been implicated as a key Rho effector here in both the mouse (Escuin et al., 2015, Ybot-Gonzalez et al., 2007) and the chick (Kinoshita et al., 2008) because of sensitivity to the ROCK inhibitor Y27632. However, our phenotypic observations, coupled with the reported sensitivity of PKN2 to Y27632 inhibition (Davies et al., 2000, Falk et al., 2014), place PKN2 as a potentially important Rho effector kinase in this context.

From an evolutionary perspective, *Drosophila* has a single PKN ortholog (dPkn), which is essential during embryogenesis, with mutant flies exhibiting dorsal closure failure (Lu and Settleman, 1999). In flies, dorsal closure occurs via a mechanism apparently requiring both contraction of the underlying amnioserosa and migration of the overlying epithelial layers (Jacinto et al., 2002). Considering the parallels between *Drosophila* dorsal closure and mammalian neural tube closure, it is tempting to speculate that PKN2 acts as the mammalian ortholog to dPkn in this context.

The genetic ablation of the PKN family reported here completes the description of KOs for the entire PKC superfamily in mice and reveals that PKN2 is one of only two family members essential for viability; atypical PKCλ (PKCι in *Homo sapiens*) KOs also die mid-gestation with gross morphological abnormalities (Soloff et al., 2004). The finding that mammalian PKN2 is essential also unifies the requirement for Rho family-responsive PKC activity from yeast to man and highlights a surprising degree of isoform selectivity within the PKN family.

## Experimental Procedures

### Mice

Studies in animals were approved by the Animal Ethics Committee of the London Research Institute (now the Francis Crick Institute) and the UK Home Office. PKN1 and PKN3 KO mice were generated by homologous recombination, and PKN2-targeted KO-first ESCs were purchased from KOMP consortium. Tie2-Cre and SM22α-Cre were provided by Taija Makinen and Ralf Adams. Rosa26CreERT2 mice (Gt(ROSA)26Sortm9(cre/ESR1)Arte) are from Taconic Biosciences. All mice were backcrossed to a C57BL/6J background.

### Pathology and Immunostaining

Embryos and tissues were stained according to standard techniques. For PKN1, PKN2, and PKN3 in situ staining, an RNAscope 2.0 formalin fixed paraffin embedded assay kit was used (Advanced Cell Diagnostics) according to the manufacturer’s instructions. Whole-mount ErbB3 in situ staining was carried out as described elsewhere (Pryor et al., 2014). Images were acquired on a 3DHISTECH slide scanner, Nikon Eclipse, or Zeiss 710 confocal microscope.

### Cell Biology and Proteomics

MEF and ESC lines were derived according to standard protocols (see the Supplemental Information). Cell growth and viability were assessed by cell counting and MTT (3-(4,5-dimethylthiazol-2-yl)-2,5-diphenyltetrazolium bromide). For immunoblots, proteins were resolved by SDS-PAGE, transferred to nitrocellulose, and incubated with appropriate antibodies before enhanced chemiluminescence visualization. For cell cycle (propidium iodide), BrdU incorporation, and mitosis, cells were fixed in ice-cold 70% ethanol, stained with appropriate antibodies, and assessed by fluorescence-activated cell sorting. Migration was followed by time lapse (Essen IncuCyte). For RT-PCR, RNA (QIAGEN) was transcribed to cDNA and amplified with TaqMan RT and Sybr Green PCR Mix (Applied Biosystems). Quantification and analysis of phosphopeptides was performed by LC-MS/MS (Casado et al., 2013, Rajeeve et al., 2014).

### Statistical Methods

For two-group comparisons, statistical significance was assessed using paired (cell cycle) or unpaired (viability, embryo quantifications, and protein expression or phosphorylation) Student’s t tests; for multiple comparisons (growth curves and migration), statistical significance was assessed by ANOVA (GraphPad Prism). For phosphoproteomics, statistical significance across four independent biological replicates was assessed with a Benjamini and Hochberg adjusted p value < 0.05.

Full details are in the Supplemental Experimental Procedures.

## Author Contributions

A.J.M.C. and P.J.P. conceived the project and wrote the manuscript. I.Q., A.J.M.C., and J.J.T.M. conducted the majority of the experiments. S.L., A.C., A.J.M.C., and I.R. derived the KO mice. C.F. and H.G. conducted cardiovascular studies. M.H., P.B., and J.T.W. assisted with cell migration. S.E. and A.J.C. conducted neural crest analysis. B.S.-D. conducted dissections and pathology with G.S. V.R. and P.C. contributed the MS analysis.

## Supplemental Information

Document S1. Supplemental Experimental Procedures, Figures S1–S4, and Tables S1–S4

Document S2. Article plus Supplemental Information
