# Supplementary material for: Knockout of the PKN Family of Rho Effector Kinases Reveals a Non-redundant Role for PKN2 in Developmental Mesoderm Expansion
Source: Cell Rep. 2016 Jan 7;14(3):440–8. doi: 10.1016/j.celrep.2015.12.049 (PMC4733087; doi:10.1016/j.celrep.2015.12.049)
Supplement: Document S1. Supplemental Experimental Procedures, Figures S1–S4, and Tables S1–S4 [file mmc1.pdf]

Cell Reports

Supplemental Information

Knockout of the PKN Family of Rho Effector Kinases Reveals a Non-redundant Role for PKN2 in Developmental Mesoderm Expansion

Ivan Quétier, Jacqueline J.T. Marshall, Bradley Spencer-Dene, Sylvie Lachmann, Adele Casamassima, Claudio Franco, Sarah Escuin, Joseph T. Worrall, Priththivika Baskaran, Vinothini Rajeeve, Michael Howell, Andrew J. Copp, Gordon Stamp, Ian Rosewell, Pedro Cutillas, Holger Gerhardt, Peter J. Parker, and Angus J.M. Cameron

Figure S1

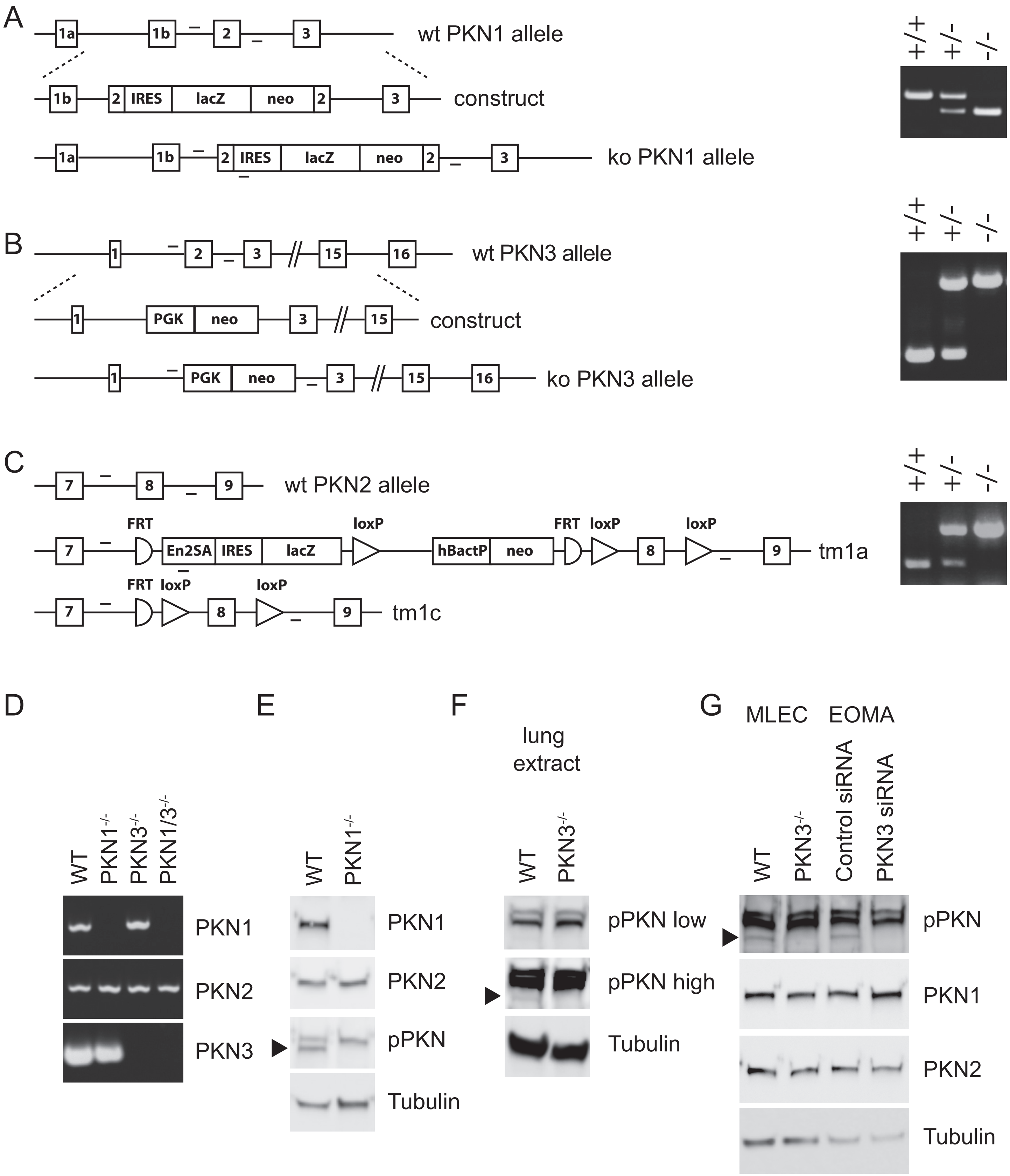

A
1a
1b
2
3
wt PKN1 allele
construct
IRES
lacZ
neo
ko PKN1 allele
+/+
+/-
-/-
B
1
15
16
wt PKN3 allele
PGK
ko PKN3 allele
C
7
8
9
wt PKN2 allele
FRT
loxP
En2SA
hBactP
tm1a
tm1c
D
WT
PKN1-/-
PKN3-/-
PKN1/3-/-
PKN1
PKN2
PKN3
E
pPKN
Tubulin
F
lung extract
pPKN low
pPKN high
G
MLEC
EOMA
Control siRNA
PKN3 siRNA

Figure S1. Generation of PKN1, PKN2 and PKN3 knockouts, related to figure 1. Schematic representation of the wild-type (wt) and targeted alleles for the three mouse PKN genes. For PKN1 (A) and PKN3 (B) a representation of the targeting construct is also provided. The PKN2 tm1a allele is a knockout first allele obtained from the KOMP consortium (C). WT and targeted alleles for each of the three PKN isoforms can be distinguished by PCR (right hand panels). The position of the primers used for PCR are indicated on the schematic and details of the primers and product sizes are provided in supplementary experimental procedures. RT-PCR demonstrating disruption of PKN1 and PKN3 mRNA extracted from mouse lungs (D). Specific primers used are detailed in supplementary experimental procedures. PKN expression in wt and PKN1 knockout (-/-) mouse embryo extracts was assessed by immunoblot (E). PKN3 was undetectable in mouse embryos but could be detected using a phospho-PKN specific antibody in mouse lung extracts (F) and mouse lung endothelial cells (G). To positively identify PKN3, protein was depleted by siRNA from murine EOMA cells. Detection of α-tubulin was used to control for protein loading.

Figure S2

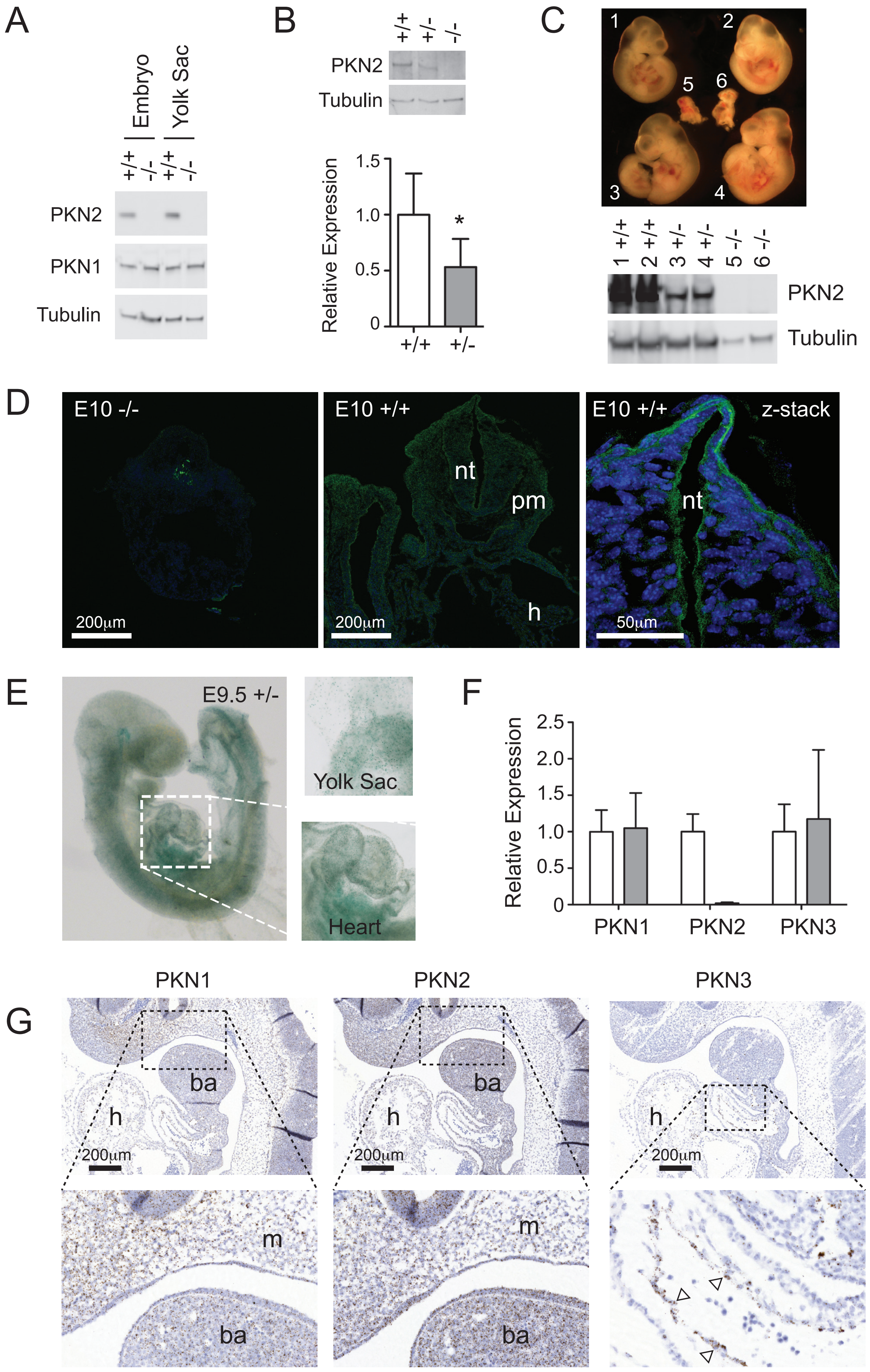

A
Embryo
Yolk Sac
+/+
-/-
+/+
-/-
PKN2
PKN1
Tubulin
B
+/+
+/-
-/-
PKN2
Tubulin
Relative Expression
1.5
1.0
0.5
0
*
+/+
+/-
C
1
2
3
4
5
6
1 +/+
2 +/+
3 +/-
4 +/-
5 -/-
6 -/-
PKN2
Tubulin
D
E10 -/-
200μm
E10 +/+
nt
pm
h
200μm
E10 +/+
z-stack
nt
50μm
E
E9.5 +/-
Yolk Sac
Heart
F
Relative Expression
2.5
2.0
1.5
1.0
0.5
0
PKN1
PKN2
PKN3
G
PKN1
PKN2
PKN3
ba
h
200μm
m
ba
ba
h
200μm
m
ba
h
200μm

Figure S2. PKN2 is broadly expressed and is required in the embryo proper, related to figure 1. PKN2 protein is expressed in the embryo and yolk sac (A) and is expressed at approximately half wild-type levels in heterozygous embryos (B); error bars represent S.D.; * < P 0.05, n=4). A single tetraploid rescue litter comprising two wild-type, two heterozygous and two knockout embryos (C). Genotypes were determined directly from genomic DNA isolated from the embryos. To assay for PKN2 protein, embryo lysates were subjected to Western blot analysis and α-Tubulin was used as a control for protein loading (C, bottom panels). Frozen section of E10 knockout (-/-) and wt embryos were stained with anti-PKN2 mAb (R&D systems) and an Alexa-488 secondary antibody. DAPI counterstained embryos were visualised by confocal microscopy (D). To visualise apical accumulation of PKN2, z-stack images were compiled as a projection (D, right hand panel). Whole mount x-gal staining reveals broadly ubiquitous expression in heterozygous embryos (E). Expression of PKN isoform mRNA expression was assessed in E8 embryo extracts by qPCR (F). PKN2 disruption is confirmed by loss of PKN2 mRNA. No expression compensation of PKN1 or PKN3 was observed on loss of PKN2. PKN1, PKN2 and PKN3 mRNA expression in E10 embryos was assessed by RNAscope *in situ* hybridisation as described in supplementary experimental procedures (G). Broad expression of PKN1 and PKN2 can be seen in all tissues whereas PKN3 is restricted; open arrow heads indicate high PKN3 expression in the endocardial lining of the bulbus arteriosus. Abbreviations: nt, neural tube; pm, pharyngeal mesoderm; h, heart; ba, branchial arch; m, mesenchyme.

Figure S3

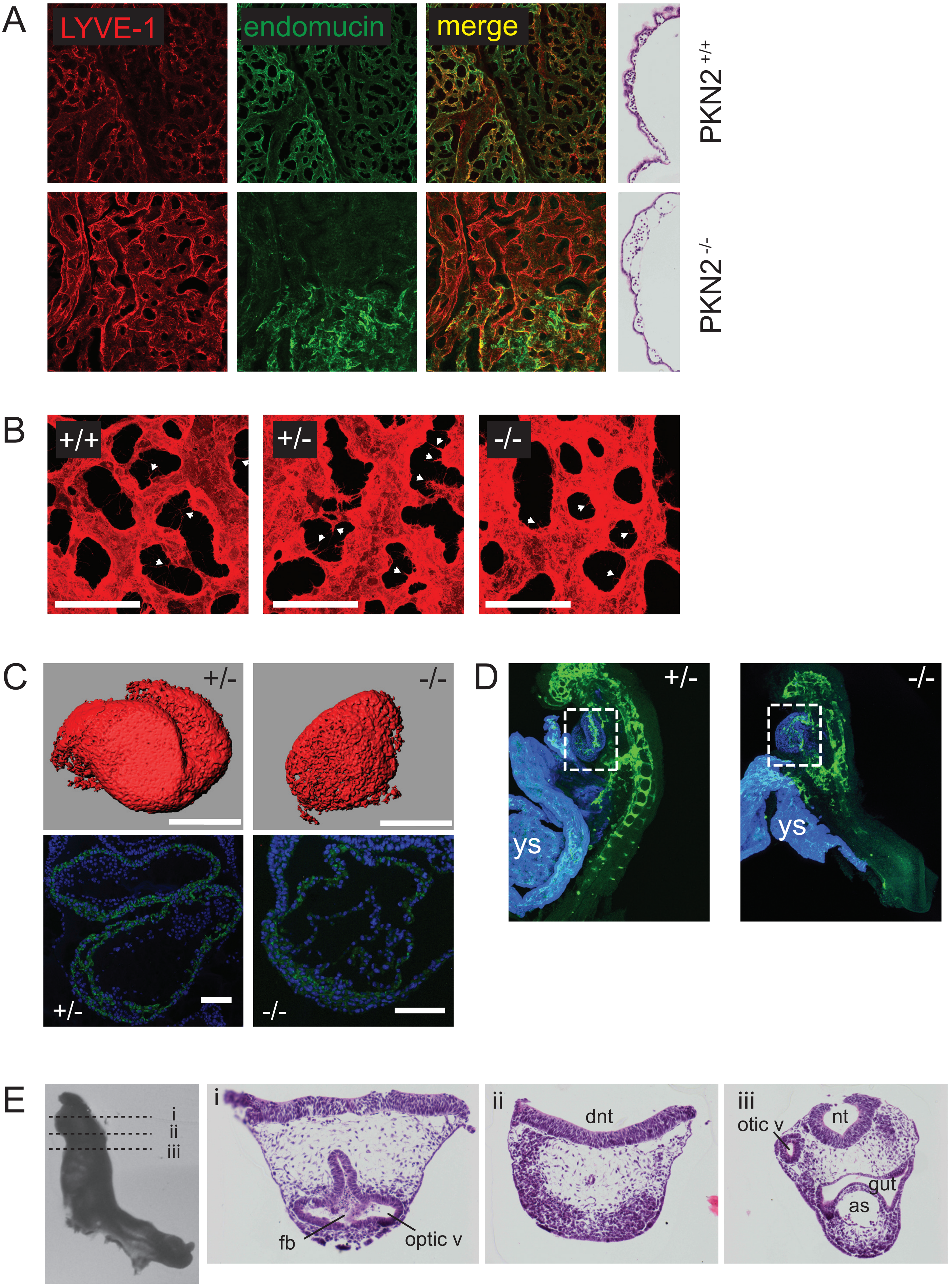
A
LYVE-1
endomucin
merge
PKN2 +/+
PKN2 -/-
B
+/+
+/-
-/-
C
+/-
-/-
+/-
-/-
D
+/-
-/-
ys
ys
E
i
ii
iii
i
fb
optic v
ii
dnt
iii
otic v
nt
gut
as

Figure S3. PKN2 knockout yolk sacs exhibit an immature vascular plexus and heart development is retarded, related to figure 1. Yolk sac endothelial cells were stained with endomucin and anti-LYVE-1 antibodies (A). Yolk sacs from E9.5 embryos were also paraffin embedded and stained with H&E (A, right hand panels). High magnification images of LYVE-1 staining reveal endothelial protrusions, indicated by arrows (B). To examine the delay in cardiac looping, E8.5 embryos were whole mount stained for desmin and 3D reconstructions were processed using Bitplane Imaris software (C, upper panels). Transverse sections were also stained for desmin followed by Alexa-488 secondary to reveal cardiomyocytes (C; lower panels). (D) Whole mount staining of PKN2 heterozygous and knockout embryos and yolk sacs (ys) with desmin (blue - Alexa 647) and endomucin (green – Alexa 488). Desmin stained hearts (boxed) are un-looped in the knockout embryos compared to littermates. Sections were counterstained with DAPI. Histological H&E transverse sections through the head of an E9.5 PKN2 knockout embryo revealed a fully open hind-brain neural tube (E). Abbreviations are: ys, yolk sac; otic v, otic vesicle; optic v, optic vesicle; nt, neural tube; dnt, dorsal neural tube; fb, forebrain; as, aortic sac.

Figure S4

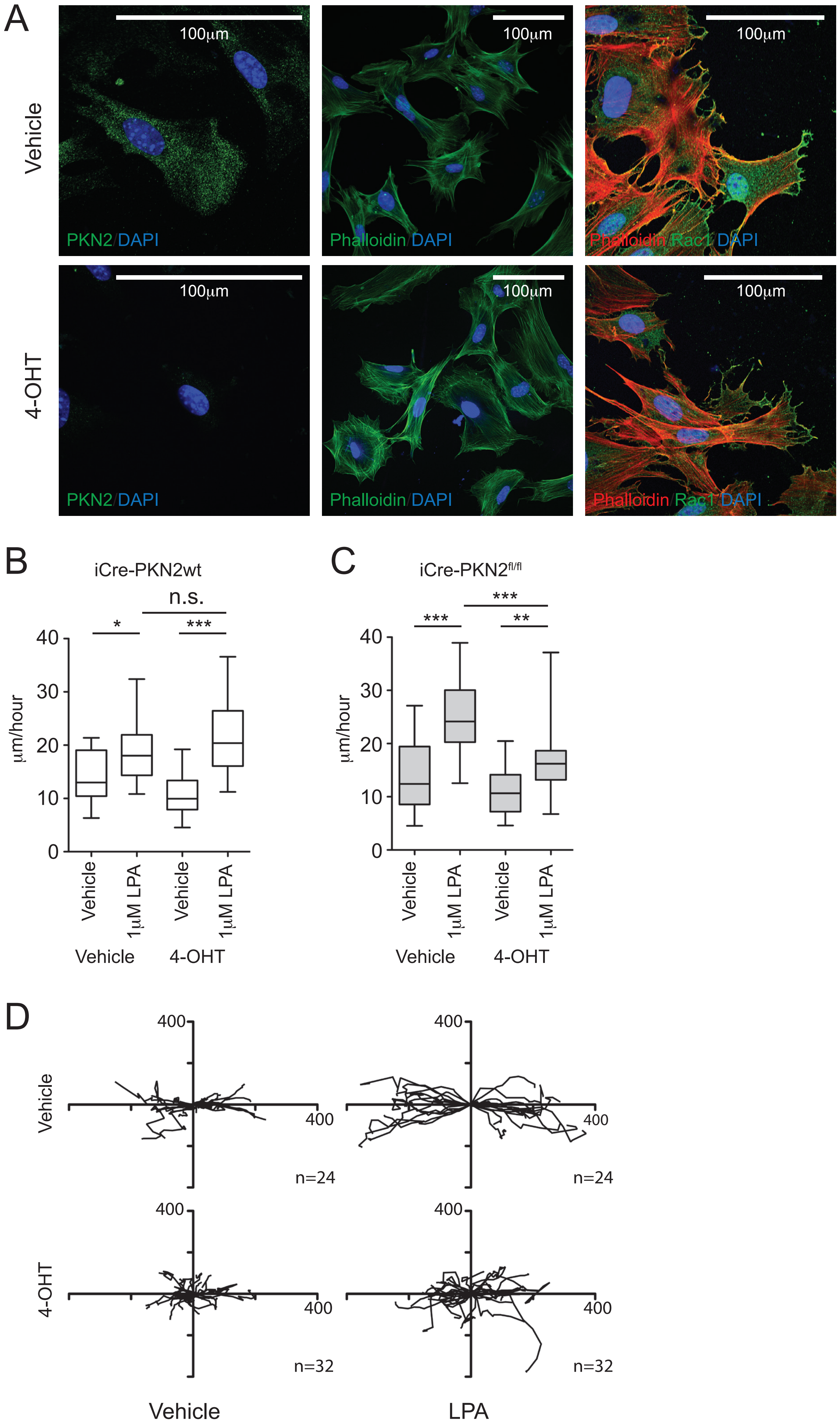

A
Vehicle
4-OHT
100µm
PKN2/DAPI
Phalloidin/DAPI
Phalloidin/Rac1/DAPI
B
iCre-PKN2wt
n.s.
*
***
µm/hour
40
30
20
10
0
Vehicle
1µM LPA
Vehicle
1µM LPA
Vehicle
4-OHT
C
iCre-PKN2fl/fl
***
***
**
µm/hour
Vehicle
1µM LPA
Vehicle
1µM LPA
Vehicle
4-OHT
D
Vehicle
4-OHT
400
n=24
n=32
Vehicle
LPA

Figure S4. Effects of PKN2 deletion on actin organisation and LPA induced migration, related to figure 3. The actin cytoskeleton remains largely unaffected by 4-OHT induced PKN2 deletion (A). MEFs were methanol fixed and stained with anti-PKN2 mAb and alexa-488 labelled secondary, and DAPI, prior to confocal imaging (left panels); 4-OHT induced deletion of PKN2 (left bottom panel). Following PFA fixation, F-actin was stained with FITC-phalloidin (actin) and nuclei with DAPI (A, middle panels). Rac1 and actin are localised at the leading edge of migrating cells in PKN2 expressing or PKN2 deleted cells (A, right panels). Cells were stained with anti-Rac1 mAb and alexa-488 labelled secondary, Alexa-555 labelled phalloidin and DAPI. Single cell tracking reveals that PKN2 deletion supresses LPA induced migration speed of MEFs in scratch wound assays (B-D); 4-OHT induced Cre activity reduces migration of $PKN2^{fl/fl}$ but not PKN2wt cells. Box and whisker plots indicate the average, quartiles and range of individual cell speed; *<P 0.05; **p<0.01;***p<0.001 ANOVA. Single cell tracks from both wound edges are displayed for the iCre-$PKN2^{fl/fl}$ cells (D; axes indicate distance in μm).

Parent Genotype (male and female)	Age	Offspring/ Embryos	Wild-Type	Heterozygous	Knockout/ Homozygous
PKN1 $^{+/-}$	-	91	21	44	26
PKN3 $^{+/-}$	-	168	43	87	38
PKN1 $^{+/-}$ PKN3 $^{+/-}$	-	222	20	190	12
PKN2$^{tm1a\ +/-}$	-	171	81	90***†	0***
PKN2$^{tm1c\ +/fl}$	-	36	11	18	7
PKN2$^{tm1a\ +/-}$	E8.5	30	9	13	8
PKN2$^{tm1a\ +/-}$	E9.5	76	22	34	20
PKN2$^{tm1a\ +/-}$	E10.5	23	6	15	2
PKN2$^{tm1a\ +/-}$	E11.5+	56	15	41	0***

Table S1. Disruption of the three PKN genes in mouse reveals that PKN2 is essential in development, related to figure 1. All mice are on a C57B/6J background. For PKN1 $^{+/-}$ PKN3 $^{+/-}$ double het crosses, wild-type and knockout numbers refer to homozygosity at both alleles; heterozygous numbers indicate heterozygosity at either allele. PKN2^{tm1a} is a knockout first allele (KOMP), which is converted to a floxed (fl) conditional allele by flp recombinase to give pkn2^{tm1c}. †On this background there was a partially penetrant haplo-insufficiency despite adult wt and heterozygous mice being fertile, normal weight and overtly indistinguishable. Statistically significant deviation for expected numbers are indicated by asterisks; ***P < 0.001; χ^2 test.

Parent Genotypes	Age	Offspring/ Embryos	Cre negative		Cre positive	
			PKN2$^{fl/+}$	PKN2$^{fl/fl}$	PKN2$^{fl/+}$	PKN2$^{fl/fl}$
Tie2-Cre$^{+/-}$PKN2$^{fl/+}$ x PKN2$^{fl/fl}$	-	45	8	9	16	12
SM22-Cre$^{+/-}$PKN2$^{fl/+}$ x PKN2$^{fl/fl}$	-	48	48	31	40	7***
SM22-Cre$^{+/-}$PKN2$^{fl/+}$ x PKN2$^{fl/fl}$	E9.5	16	2	5	4	5
	E11.5	19	5[a]	6	13	4[b]
	E13.5	8	3	2	1	2
	E9.5-13.5	52	10	13	18	11
	Expected Fraction:		0.25	0.25	0.25	0.25

Table S2. Conditional PKN2 knockout offspring and embryo numbers, related to figure 2. All mice are on a C57B/6J background. Statistically significant deviation for expected numbers are indicated by asterisks; ***$P < 0.001$; χ^2 test. [a]one embryo malformed and dead; [b]one embryo small and dead.

Gene name and phosphorylation site identified/Protein name/Description and processes regulated.	Vehicle: 0.5% Serum	4-OHT: 0.5% Serum	Vehicle: 10% Serum	4-OHT: 10% Serum	Fold change in serum (Vehicle/4-OHT)
**Pkn2 pS582**: Deleted following 4-OHT treatment.				**	55.3
**Zc3hc1 pS394**: NIPA; E3 ligase complex component – mitotic entry.				**	17.6
**Anln pS180**: Anillin; structural integrity of cleavage furrow – cytokinesis.		***		***	12.9
**Top2a pT1350**: Topoisomerase 2; chromosome segregation –mitosis.		***		**	11.3
**Tpx2 pS486**: Spindle assembly factor and microtubule nucleation – mitosis.				*	8.5
**Anln pS293**: Anillin; structural integrity of cleavage furrow – cytokinesis.		*		*	7.9
**Tpx2 pS737**: Spindle assembly factor and microtubule nucleation – mitosis.		*		*	6.2
**Ska3 pS154**: Kinetochore complex component – mitsosis.		*		**	5.6
**Pkn2 pS619**: Deleted following 4-OHT treatment.		**		*	5.3
**Rgcc pS97**: Regulator of Cell Cycle; enhances cdk1 activity – cell cycle.		*		**	4.9
**Arpp19 pS23**: cAMP regulated pp19; mitotic PP2A inhibitor - mitosis		***		**	4.7
**Anln pS180**: Anillin; structural integrity of cleavage furrow – cytokinesis.		**		*	4.3
**Lig1 pS49**: DNA ligase 1; seals nicks during DNA replication – S-phase.		**		**	4.2
**Cep55 pS428**: Centrosomal protein required for mitotic exit - mitosis		*		***	3.8
**Lig1 pT77**: DNA ligase 1; seals nicks during DNA replication – S-phase.					3.6
**Ercc6l pS1021**: Spindle checkpoint helicase binding Mad2 – mitosis.		**		*	3.4
**Lig1 pS51**: DNA ligase 1; seals nicks during DNA replication – S-phase.		**		**	3.2
**Lig1 pS188**: DNA ligase 1; seals nicks during DNA replication – S-phase.		*		**	2.8
**Mcm3 pS672**: DNA replication licencing factor – replicative helicase – S-phase.				***	2.6
**Ncapd2 pS1320**: Condensin complex subunit 1 – mitosis.		**		*	2.6

**Mcm3 pS672**: DNA replication licencing factor – replicative helicase – S-phase		*		**	2.5
**Mcm2 pS41**: DNA replication licencing factor – replicative helicase – S-phase				*	1.7
**Mcm2 pS21**: DNA replication licencing factor – replicative helicase – S-phase		*		*	1.7
**Hmga2 pS101 pS104**: Transcriptional regulator - cell cycle		*			0.8
**Pard3 pS174**: Cell polarisation complex protein – cell cycle.		*			0.7
**Pard3 pS221**: Cell polarisation complex protein – cell cycle.				*	0.6
**Mcm2 pT39 pS41**: DNA replication licencing factor; helicase – S-phase.		*			0.5
**Hmga2 pT99 pS100 pS101**: Transcriptional regulator - cell cycle				**	0.4
**Arhgef2 pS955**: Rho GTPase exchange factor regulating division – mitosis.				**	0.1

-5	-4	-3	-2	-1	0	1	2	3	4	5

Key: Relative Fold Change

Table S3. Summary of cell cycle and mitosis associated phosphopeptides significantly modulated by PKN2 deletion, related to figure 3. PKN2 iMEFs were treated with 4-OHT or Vehicle, cultured for 48 hours to allow PKN2 loss, serum starved overnight and, where indicated, re-stimulated with serum for 30 minutes prior to cell lysis. Phosphopeptides were analysed by LC-MS/MS across 4 biological replicates. Hits are compiled from combined cell division (GO:0051301), mitosis (GO:0007067) and cell cycle (GO:0007049) ontologies. Only peptides significantly modulated by PKN2 loss are included and hits are ordered according to average fold change after 10% serum re-stimulation. Colours represent phosphopeptide abundance relative to the sum of the four conditions. Asterisks indicate significant differences between Vehicle and 4-OHT treatments under either serum free or serum stimulated conditions as indicated; Benjamini & Hochberg corrected p-values: * <0.05; **<0.01; ***<0.001.

**Gene name and phosphorylation site identified/Protein name/Description and processes regulated.**	Vehicle: 0.5% Serum	4-OHT: 0.5% Serum	Vehicle: 10% Serum	4-OHT: 10% Serum	Fold change in serum (Vehicle/4-OHT)
**Anln pS180:** Anillin; actin binding protein that regulates cytoskeletal dynamics.		***		***	12.9
**Anln pS293:** Anillin; actin binding protein that regulates cytoskeletal dynamics.		*		*	7.9
**Myo18a pS83:** Myosin 18a; modulates lamellar actomyosin - cell protrusion and migration		**		*	3
**Ppp1r18 pS212:** Protein Phosphatase 1, Regulatory Subunit 18; targets PP1 to F-actin				*	2.5
**Map1b pS1293 pS1307:** Microtubule-Associated Protein 1B; microtubule assembly		**		**	2.4
**Nes pS169:** Nestin; intermediate filament protein - cytoskeleton		**		*	2.4
**Flna pS1084:** Filamin A; actin-binding protein, links actin to membrane		*		***	2.3
**Cgn pS131:** Cingulin; *actin binding* – cytoskeletal dynamics		*			2.2
**Nes pS731:** Nestin; intermediate filament protein – cytoskeletal dynamics		*		***	2
**Nes pS688:** Nestin; intermediate filament protein – cytoskeletal dynamics		**		**	2
**Nes pS623:** Nestin; intermediate filament protein – cytoskeletal dynamics		*		**	1.8
**Zyx pS336:** Zyxin; concentrates at focal adhesions – cytoskeletal organization				*	1.6
**Palld pS782:** Palladin; component of actin-containing microfilaments - cytoskeletal dynamics				**	1.6
**Fhod1 pS502:** Formin Homology 2 Domain Containing 1; Role in assembly of F-actin structures				**	0.9
**Svil pS960:** Supervillin; links actin cytoskeleton and the membrane – cytoskeletal dynamics				*	0.7
**Epb41l1 pS782:** Erythrocyte Membrane Protein Band 4.1-Like 1 – actin binding		*		**	0.7
**Ssh3 pS639:** Slingshot Protein Phosphatase 3 – actin filament dynamics		**		***	0.4
**Ppp1r18 pS224**: Protein Phosphatase 1, Regulatory Subunit 18; targets PP1 to F-actin				*	0.3
**Myo1h pT356 pS364 pS365:** Myosin-1H; actin-based motor molecules with ATPase activity				**	0.2
**Arhgef2 pS955:** Rho/Rac Guanine Nucleotide Exchange Factor (GEF) 2; Activates Rho-GTPases				**	0.1

-5	-4	-3	-2	-1	0	1	2	3	4	5

Key: Relative Fold Change

Table S4. Summary of cytoskeleton, intermediate filament and focal adhesion associated phosphopeptides significantly modulated by PKN2 deletion, related to figure 3. PKN2 iMEFs were treated with 4-OHT or Vehicle, cultured for 48 hours to allow PKN2 loss, serum starved overnight and, where indicated, re-stimulated with serum for 30 minutes prior to cell lysis. Phosphopeptides were analysed by LC-MS/MS across 4 biological replicates. Hits are compiled from combined actin cytoskeleton (GO:0031523; GO:0003779), focal adhesion (GO:0005925), microtubule (GO:0001578) and intermediate filament (GO:0005882) ontologies. Only peptides significantly modulated by PKN2 loss are included and hits are ordered according to average fold change after 10% serum re-stimulation. Colours represent phosphopeptide abundance relative to the sum of the four conditions. Asterisks indicate significant differences between Vehicle and 4-OHT treatments under either serum free or serum stimulated conditions as indicated; Benjamini & Hochberg corrected p-values: * <0.05; **<0.01; ***<0.001.

SUPPLEMENTARY EXPERIMENTAL PROCEDURES

Mice

To generate PKN1 null mice, a HindIII/SpeI fragment of the PKN1 genomic sequence was cloned and an IRES/β-Galactosidase/neomycin cassette was introduced into a SmaI site in the second exon; PKN1 has two alternate transcripts which differ in their first exon usage (termed 1a and 1b) and the disrupted exon is the first exon common to both transcripts. PKN1 targeted GK129 Embryonic Stem clones (PKN1$^{+/-}$) were isolated and injected into C57BL/6 blastocysts to generate chimeras. Several chimeras gave germ line transmissions, which were used to establish the homozygous PKN1-deficient mouse line. Mice were backcrossed onto C57BL/6 for a minimum of 6 generations. Genotypic characterization of recombinant ES cells and adult mice was confirmed by Southern blot analysis of KpnI-digested genomic DNA. Mice were genotyped from ear clip genomic DNA with PKN1 specific sense (PKN1-F; 5'-AGAGCACTATGTGTTTTTAGCTGCTGAGCC-3') and antisense (PKN1-R; 5'-TGTCCTTAGGTATCTGGTGACAGTGTGGG-3') primers and an IRES cassette antisense (IRES-R; 5'-GCTAGACTAGTCTAGCTAGAGCGGCC-3') to generate a 900bp fragment for the wt locus (PKN1-F with PKN1-R) and a 450bp fragment for the PKN1 ko locus (PKN1-F with IRES-R).

To generate PKN3 null mice, a fragment of the PKN3 genomic sequence was subcloned from a BAC clone (BACPAC RP24-364D12) into the NotI and XhoI sites of pFlrt (Ralf Adams); the PKN3 fragment was bounded by the forward cloning primer, SHA-NotI-F (5'-ATAAGAATGCGGCCGCGTTACCGTGGAATATGCCACCG-3') and a naturally occurring XhoI site situated between exon 15 and exon 16 of PKN3. The coding sequence of Exon 2 was replaced by a PGK-neo cassette, to generate the PKN3 targeting construct using the Quick and Easy Conditional Knockout Kit (Gene Bridges) according to the manufacturers instructions. PKN3 targeted Primogenix B6.1 (C57Bl/6N) ES cells were identified by PCR

screening and injected into C57Bl/6 blastocysts to generate chimeras and germ line transmission. Correct integration was confirmed by multiple diagnostic PCR of genomic DNA using primers flanking both the short and long homology arms of the PKN3 knockout construct. Mice were genotyped from earclip genomic DNA using PKN3 specific sense (PKN3-F3; 5'- CCAGAAGGCACTGGGCGAAC -3) and antisense (PKN3-R1; 5'-CAACACAAGGCTAGAGTTCA -3) primers to generate a 540bp fragment for the wt locus and a 2kb fragment for the ko locus.

To generate PKN2 null mice targeted ES cells were obtained from the KOMP Repository (www.komp.org: Project ID66263 - pkn2 MGI:109211). Two independent knockout first ES cell clones underwent germline transmission: clone G05 (allele: Pkn2$^{tm1a(KOMP)Wtsi}$) and clone G08 (allele: Pkn2$^{tm1e(KOMP)Wtsi}$). Clone G08 has lost the 3' loxP site necessary to allow generation of a conditional mouse through FLP/FRT recombination so provides a null allele only. Mice were genotyped from ear clip genomic DNA with PKN2 specific sense (PKN2-F2; 5'- GGTTTGGTGACCAGTAAAAACTG -3') and antisense (PKN2-R1; 5'-GGAATACAGAACAAGGCAAAAGC -3') primers and an insert specific antisense primer (En2R; 5'-CCAACTGACCTTGGGCAAGAACAT -3') to generate a 1089bp fragment for the wt locus and a 395bp fragment for the PKN2 ko locus. To convert the PKN2 to a conditional allele, PKN2 heterozygous mice were crossed with a Flp deleter mouse (Tg(CAG-Flpo)1Afst; background C57Bl/6N); to genotype, sense primer PKN2-F2 was used with a second gene specific antisense primer (PKN2-R2; 5'- CTGAAGACACTTTGAAAAGGATG -3') to generate 489bp and 635bp products for the wt and conditional alleles respectively.

Tetraploid embryos for rescue experiments were generated by fusing 2 cell embryos (B6CBAF1 x F1) on the Cell-fusion instrument, CF-150B available from BLS Ltd., Hungary. Successfully fused embryos were held in culture and allowed to develop to the 8 cell

stage. 8 cell stage tetraploid embryos and PKN2 embryos (stemming from heterozygote x heterozygote matings) were rendered zona free with Acid Tyrodes solution before aggregation of 1-2 tetraploid embryos with each PKN2 embryo according to standard protocols (Nagy and Rossant, 1993). Aggregated embryos were transferred into pesudopregnant surrogate mice.

Tie2-Cre (Koni et al., 2001) and SM22α-Cre mice (Lepore et al., 2005) on a C57BL/6 background were kindly provided by Taija Makinen and Ralf Adams. Rosa26CreERT2 mice (Gt(ROSA)26Sortm9(cre/ESR1)Arte) are from Taconic biosciences. Cre positive mice were identified using Cre sense (Cre-F; 5'- ACTATCCAGCAACATTTGGGCC -3') and antisense (Cre-R; 5'- CCGGCAAAACAGGTAATTCGG -3') primers to generate a 450bp fragment. For *in-vivo* bromodeoxyuridine (BrdU) labeling SM22α-Cre mice were injected with 2mg BrdU i.p. 2 hours prior to sacrifice.

Antibodies and Staining

Antibodies used were: PRK1 mAb (BD biosciences), PKN2 mAb (Clone 509105; N-terminal epitope amino acids 18-207; R&D systems), PKN2 mAb (Clone 1D1; C-terminal epitope amino acids 555-718, Abcam), Tubulin mAb (Sigma; Clone B-5-1-2), LYVE-1 (Reliatech GmbH), PECAM-1 rat mAb (BD Biosciences), Desmin mAb (Dako), BrdU mAb (Dako), caspase 3 (R&D systems), Annexin V (Biolegend), phospho-Histone H3 S10 (EMD Millipore), anti-endomucin rat mAb (Santa Cruz), GAPDH (Santa Cruz), Cyclin A (Santa Cruz), Cyclin D1 (Spring Bioscience), PCNA (Oncogene Research Products), Mcm2 (Bethyl) and phospho-specific polyclonal antibodies (PRK1/2 T744, ERK, Akt, p70S6K, cdk1 Y15, eIF4E S209; Cell Signaling Technology). Secondary HRP (Amersham) and Alexa 488/555/647 (Invitrogen) antibodies were used as appropriate. For immunostaining, embryos were fixed overnight in 10% neutral buffered formalin, embedded in paraffin; 4μm sections were treated with

sodium citrate (pH 6.0) buffer prior to immunostaining. HRP-secondary antibodies were developed with 3,3'-diaminobenzidine (DAB). Fluorescently stained sections were incubated for 30 minutes in 0.1 % Sudan Black in 70% EtOH and mounted in Hardset mount with DAPI (Vector). For β-galactosidase staining, embryos were fixed in 0.4% paraformaldehyde and stained using X-gal according to standard protocols. For standard histological analysis 4μm sections were stained with hematoxylin and eosin (H&E). For frozen sections, embryos were embedded and frozen in OCT. 8mm sections were dried at room temperature for 30 min, permeabilized with 0.1% Triton-x100 for 10 min, blocked in PBS-BSA 2% and stained with anti-PKN2mAb (R&D systems), alexa488 secondary and DAPI. Images were acquired on a Nikon Eclipse 90i upright microscope or Zeiss LSM confocal and processed using NIS-elements software, Zen and Adobe Photoshop CS5.

For whole-mount staining, embryos were dissected, fixed in 4% PFA, permeabilised with 0.5% triton X100, blocked with PBSMT (PBS supplemented with 3% milk and 0.1% Tween 20) and stained overnight with appropriate primary antibodies. For PECAM stains embryos were then washed and incubated overnight with a peroxidase-conjugated goat anti-rat IgG (Pierce), washed and developed with Substrate G (Vector) and cleared in glycerol, based on published protocols (Adams et al., 1999). Images were acquired on a Nikon Eclipse 90i upright microscope and processed using NIS-elements software. For fluorescent staining appropriate alexa 488/555/647 were used as indicated and confocal images were acquired on a Zeiss LSM 710/780 inverted microscope and processed using ImageJ, Zen and Adobe Photoshop; 3D reconstructions of desmin stained hearts were processed using Bitplane Imaris software.

***In-situ* hybridization**

For PKN1, PKN2 and PKN3 in-situ staining, an RNAscope 2.0 FFPE assay kit was used

(Advanced Cell Diagnostics) according to the manufacturers instructions. The sections were incubated with RNAscope custom oligonucleotide probes: Mouse PKN1, accession no. NM_001199593.1, target region 384-1432; mouse PKN2, accession no. NM_178654.4, target region 634-1628; mouse PKN3, accession no. NM_153805.1, target region 401-1352. Probes were incubated for 2 hours at 40°C and signals developed according to manufacturers instructions using the RNAscope 2.0 HD Assay- BROWN system and hematoxylin counterstain. Whole-mount *in-situ* staining of ErbB3 was carried out as elsewhere described (Pryor et al., 2014).

Cells and treatments

For siRNA depletion of mouse PKN3, EOMA cells were transfected using 10 nM Dharmacon ON-TARGET plus siRNA smartpool (4 duplexes: #1: 5'- GCACCGACUUCGAGUAGAG -3'; #2: 5'- GGAAGGAAUUGGCUUUGGU -3'; #3: 5'- GGACACUUGUGAAACCUAU -3'; #4: 5'- GUACAAAGGAACAGGGAAA -3') using HiPerfect (Qiagen). Cells were harvested after 48 hours and examined by immunoblot. Mouse lung endothelial cells (MLEC) were isolated and cultured as described by Reynolds et al (Reynolds and Hodivala-Dilke, 2006). For mouse embryonic fibroblasts (MEF), embryos were decapitated and foetal liver was removed prior to trypsin digestion and serial passage in DMEM with 10% FBS. Lines were immortalised using a 3T3 protocol of serial passage and subsequent senescence escape. ES cells were isolated from blastocysts collected from heterozygous PKN2$^{fl/+}$ RosaCreERT crosses to enable inducible knockout of PKN2 in culture. Cells were derived as described (Bryja et al., 2006) with the addition of a MEK inhibitor (PD0325901) for the initial stages to improve plating efficiency. After 4 passages the cells were grown in standard, serum containing ES cell media. Male cell lines with a good chromosome count were selected. Induction of Cre in RosaCreERT cells was achieved through incubation with 4-hydroxytamoxifen (4-OHT;

typically 100nM) for 1 hour prior to media replacement.

Immunoblotting

Lysates were resolved by SDS page and transferred to PVDF or nitrocellulose membranes. Immunoblots were blocked in TBST (TBS containing 0.1% Tween 20) with 3% BSA and probed with primary antibodies as indicated. Following incubation with appropriate HRP conjugated secondary antibodies (Amersham), bands were visualised by ECL using an Image Quant digital imaging system (GE healthcare) or X-ray film.

Cell Growth and viability

Cell growth was assessed by cell counting, MTT (Methylthiazolyldiphenyl-tetrazolium), and FACs analysis. For MTT assays, cell grown in 96 well plates were incubated with 1mM MTT (Sigma) for 1 hour prior to DMSO solubilisation of formazan product and 550nm absorbance measurement (Tecan). For cell colony staining, monolayers were fixed with 4%PFA and incubated with 0.1% Crystal Violet for 30 minutes.

Cell cycle and apoptosis

For cell cycle and subG1 analysis, cells were fixed in ice cold 70% ethanol, washed with PBS and RNAse A treated prior to staining with propidium iodide. For 5-Bromo-2´-Deoxyuridine (BrdU) analysis, cells were incubated in culture with 10μM BrdU prior to ethanol fixation, acid treatment (2M HCl) and anti-BrdU mAb followed by alexa-488 secondary staining. Mitotic nuclei were stained with anti-phospho-Histone H3 (S10) antibody and alexa-488 secondary. For BrdU and mitotic assays, cell cycle was simultaneously assessed as described above. To measure apoptosis, cells were stained with alexa-647 conjugated anti-annexin V antibody (Biolegend) and propidium iodide in appropriate buffer (10 mM HEPES, pH 7.4; 140 mM NaCl; 2,5 mM $CaCl_2$). Data were acquired on an LSRII fortessa (BD biosciences)

and analysed using FlowJo.

Cell Motility

For migration analysis, confluent cell monolayers were scratch wounded in 96 well format using a 96-well Wound–maker (Essen). Wound closure was followed by time-lapse video capture on an Essen IncuCyte. Individual cells were tracked using the Manual Tracking plugin for ImageJ. A combined minimum of thirty, non-mitotic cells migrating from opposing edges of the scratch were tracked from three experimental repeats for each condition.

RT-PCR

RNA was isolated from mouse lungs and embryos using the RNeasy (Qiagen) RNA isolation system and converted to cDNA using TaqMan (Applied Biosystems) reverse transcription reagents. Specific primers spanning intron exon boundaries for PKN1 and PKN3 were used to assay disruption of transcripts in PKN1 and PKN3 knockout mice. PKN2 was used as an internal control. Specific primers used were: PKN1, sense 5'- GCGACGCCGTACAGAGTGA -3'and antisense 5'- CTGCAAGGGATTGGGTAGCA -3' (336bp), PKN2; sense 5'- AGCGGCATGGCATGTGTCTCT -3'and antisense 5'- TGCGTGCGTCAACGACTGGC -3' (303bp) and PKN3; sense 5'- GAGCCCCCAAGGATGAGAAA -3'and antisense 5'- AGTGTCCCTGTCAAGGCAATAG -3' (790bp). For real-time quantitative assessment of PKN1, PKN2 and PKN3, cDNA was amplified with Sybr Green PCR Master Mix (Applied Biosystems). Data were normalized using the DeltaDeltaCt method with GAPDH as the housekeeping gene, and expressed as the relative mRNA level compared to the control. Primers used were: PKN1, sense 5'-ATCCGGCACACTGGAGACA-3' and antisense 5'- TGGTGGATTCATGGGTTGGAG -3'; PKN2, sense 5'- CTGTGCCTGCAACAGTGC -3' and antisense 5'- CTGTAGAGTCACTGGCCGGA-3', PKN3, sense 5'-CTTGGGAACCCCCAACCTTT-3' and antisense

5'-CAGATCTTTGCAGCCCAGGA-3' and GAPDH, 5'-GAAGCAGGCATCTGAGGGCC-3'and 5'-AGGCCATGTAGGCCATGAGG-3'.

Quantitative Phosphoproteomics

Following sample extraction and trypsin digestion of control and treated samples, TiO_2 chromatography was used to enrich phosphopeptides(Montoya et al., 2011). Identification and quantification of these phosphopeptides across quadruplicate samples was performed by LC-MS/MS as described elsewhere(Casado and Cutillas, 2011; Rajeeve et al., 2014). Statistical significance of phosphorylation site changes across conditions is assessed and sites are selected with a Benjamini & Hochberg adjusted p-value <0.05. Kinase substrate enrichment analysis (KSEA) was used to infer kinase activity from the phosphoproteomics data(Casado et al., 2013). Ontologies and pathways enriched in the phosphoproteomics data was investigated my matching to existing repositories(Ashburner et al., 2000; Schaefer et al., 2009).

REFERENCES

Adams, R.H., Wilkinson, G.A., Weiss, C., Diella, F., Gale, N.W., Deutsch, U., Risau, W., and Klein, R. (1999). Roles of ephrinB ligands and EphB receptors in cardiovascular development: demarcation of arterial/venous domains, vascular morphogenesis, and sprouting angiogenesis. Genes Dev *13*, 295-306.

Ashburner, M., Ball, C.A., Blake, J.A., Botstein, D., Butler, H., Cherry, J.M., Davis, A.P., Dolinski, K., Dwight, S.S., Eppig, J.T., *et al.* (2000). Gene ontology: tool for the unification of biology. The Gene Ontology Consortium. Nature genetics *25*, 25-29.

Bryja, V., Bonilla, S., Cajanek, L., Parish, C.L., Schwartz, C.M., Luo, Y., Rao, M.S., and Arenas, E. (2006). An efficient method for the derivation of mouse embryonic stem cells. Stem cells *24*, 844-849.

Casado, P., and Cutillas, P.R. (2011). A self-validating quantitative mass spectrometry method for assessing the accuracy of high-content phosphoproteomic experiments. Molecular & cellular proteomics : MCP *10*, M110 003079.

Casado, P., Rodriguez-Prados, J.C., Cosulich, S.C., Guichard, S., Vanhaesebroeck, B., Joel, S., and Cutillas, P.R. (2013). Kinase-substrate enrichment analysis provides insights into the heterogeneity of signaling pathway activation in leukemia cells. Science signaling *6*, rs6.

Koni, P.A., Joshi, S.K., Temann, U.A., Olson, D., Burkly, L., and Flavell, R.A. (2001). Conditional vascular cell adhesion molecule 1 deletion in mice: impaired lymphocyte migration to bone marrow. The Journal of experimental medicine *193*, 741-754.

Lepore, J.J., Cheng, L., Min Lu, M., Mericko, P.A., Morrisey, E.E., and Parmacek, M.S. (2005). High-efficiency somatic mutagenesis in smooth muscle cells and cardiac myocytes in SM22alpha-Cre transgenic mice. Genesis *41*, 179-184.

Montoya, A., Beltran, L., Casado, P., Rodriguez-Prados, J.C., and Cutillas, P.R. (2011). Characterization of a TiO(2) enrichment method for label-free quantitative phosphoproteomics. Methods *54*, 370-378.

Nagy, A., and Rossant, J. (1993). Production of completely ES cell-derived fetuses. In Gene Targeting: A practical approach, A.L. Joyner, ed. (Oxford University Press), pp. 143-179.

Pryor, S.E., Massa, V., Savery, D., Andre, P., Yang, Y., Greene, N.D., and Copp, A.J. (2014). Vangl-dependent planar cell polarity signalling is not required for neural crest migration in mammals. Development *141*, 3153-3158.

Rajeeve, V., Vendrell, I., Wilkes, E., Torbett, N., and Cutillas, P.R. (2014). Cross-species Proteomics Reveals Specific Modulation of Signaling in Cancer and Stromal Cells by Phosphoinositide 3-kinase (PI3K) Inhibitors. Molecular & cellular proteomics : MCP *13*, 1457-1470.

Reynolds, L.E., and Hodivala-Dilke, K.M. (2006). Primary mouse endothelial cell culture for assays of angiogenesis. Methods in molecular medicine *120*, 503-509.

Schaefer, C.F., Anthony, K., Krupa, S., Buchoff, J., Day, M., Hannay, T., and Buetow, K.H. (2009). PID: the Pathway Interaction Database. Nucleic acids research *37*, D674-679.
