# Supplementary material for: Knockout of the PKN Family of Rho Effector Kinases Reveals a Non-redundant Role for PKN2 in Developmental Mesoderm Expansion
Source: Cell Rep. 2016 Jan 7;14(3):440–8. doi: 10.1016/j.celrep.2015.12.049 (PMC4733087; doi:10.1016/j.celrep.2015.12.049)
Supplement: Document S2. Article plus Supplemental Information [file mmc2.pdf]

Cell Reports

Report

Knockout of the PKN Family of Rho Effector Kinases Reveals a Non-redundant Role for PKN2 in Developmental Mesoderm Expansion

Graphical Abstract

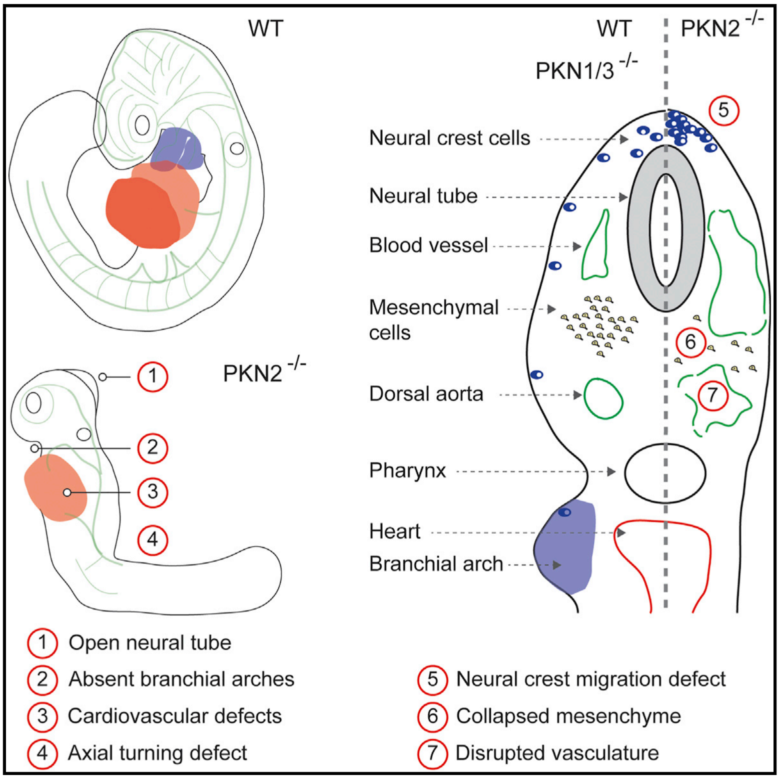

Highlights

- PKN2, but not PKN1 or PKN3, is essential during mouse embryogenesis
- PKN2 knockout causes severe cardiovascular and morphogenetic abnormalities
- PKN2 is required for mesenchymal growth and neural crest migration in vivo

Authors

Ivan Quétier, Jacqueline J.T. Marshall, Bradley Spencer-Dene, ..., Holger Gerhardt, Peter J. Parker, Angus J.M. Cameron

Correspondence

peter.parker@crick.ac.uk (P.J.P.), a.cameron@qmul.ac.uk (A.J.M.C.)

In Brief

The Rho effector PKN kinases regulate diverse cellular functions, but their in vivo function is unexplored. By systematically targeting the PKN family, Quetier et al. reveal a unique role for PKN2 during developmental growth and morphogenesis. These findings impact on developmental disorders and the targeting of PKN in disease.

Quétier et al., 2016, Cell Reports *14*, 440–448
January 26, 2016 ©2016 The Authors
http://dx.doi.org/10.1016/j.celrep.2015.12.049

OPEN ACCESS
CellPress

Cell Reports
Report

Knockout of the PKN Family of Rho Effector Kinases Reveals a Non-redundant Role for PKN2 in Developmental Mesoderm Expansion

Ivan Quétier,[1] Jacqueline J.T. Marshall,[2] Bradley Spencer-Dene,[3] Sylvie Lachmann,[2] Adele Casamassima,[2] Claudio Franco,[4] Sarah Escuin,[5] Joseph T. Worrall,[6] Priththivika Baskaran,[1] Vinothini Rajeeve,[6] Michael Howell,[3] Andrew J. Copp,[5] Gordon Stamp,[3] Ian Rosewell,[7] Pedro Cutillas,[6] Holger Gerhardt,[8] Peter J. Parker,[2,9,*] and Angus J.M. Cameron[1,*]
[1]Kinase Biology Laboratory, John Vane Science Centre, Barts Cancer Institute, Queen Mary University of London, Charterhouse Square, London EC1M 6BQ, UK
[2]Protein Phosphorylation Laboratory, Francis Crick Institute, 44 Lincoln's Inn Fields, London WC2A 3LY, UK
[3]Francis Crick Institute, 44 Lincoln's Inn Fields, London WC2A 3LY, UK
[4]Instituto Medicina Molecular (iMM), Faculdade de Medicina da Universidade de Lisboa, 1649-028 Lisbon, Portugal
[5]Newlife Birth Defects Research Centre, Institute of Child Health, University College, London WC1N 1EH, UK
[6]John Vane Science Centre, Barts Cancer Institute, Queen Mary University of London, Charterhouse Square, London EC1M 6BQ, UK
[7]Genetic Manipulation Services, Francis Crick Institute, Clare Hall, Herts EN6 3LD, UK
[8]Max-Delbrück-Center for Molecular Medicine, Robert-Rössle-Strasse 10, 13125 Berlin, Germany
[9]Division of Cancer Studies, King's College London, New Hunt's House, Saint Thomas Street, London SE1 1UL, UK
*Correspondence: peter.parker@crick.ac.uk (P.J.P.), a.cameron@qmul.ac.uk (A.J.M.C.)
http://dx.doi.org/10.1016/j.celrep.2015.12.049
This is an open access article under the CC BY license (http://creativecommons.org/licenses/by/4.0/).

SUMMARY

In animals, the protein kinase C (PKC) family has expanded into diversely regulated subgroups, including the Rho family-responsive PKN kinases. Here, we describe knockouts of all three mouse PKN isoforms and reveal that PKN2 loss results in lethality at embryonic day 10 (E10), with associated cardiovascular and morphogenetic defects. The cardiovascular phenotype was not recapitulated by conditional deletion of PKN2 in endothelial cells or the developing heart. In contrast, inducible systemic deletion of PKN2 after E7 provoked collapse of the embryonic mesoderm. Furthermore, mouse embryonic fibroblasts, which arise from the embryonic mesoderm, depend on PKN2 for proliferation and motility. These cellular defects are reflected in vivo as dependence on PKN2 for mesoderm proliferation and neural crest migration. We conclude that failure of the mesoderm to expand in the absence of PKN2 compromises cardiovascular integrity and development, resulting in lethality.

INTRODUCTION

The Rho family guanosine triphosphatases (GTPases), which includes Rho, Rac, and Cdc42, are regulators of cell shape, adhesion, and motility and as such are critical in development (Bustelo et al., 2007). Numerous studies have examined the roles of Rho family members at a biochemical and cellular level; however, their ubiquitous expression and pleiotropic roles have made elucidation of in vivo function difficult. This has in part been addressed using tissue-specific conditional alleles of Rho family members (D'Amico et al., 2009). To gain a deeper insight, it is necessary to examine the roles of specific effector pathways. Among the diverse array of effectors are many Ser/Thr kinases, which include the Rho family-activated kinases (ROCK1/2), p21-activated kinases (PAK1–PAK6), and protein kinase N (PKN) kinases (Zhao and Manser, 2005).

The PKN kinases represent a subfamily of the protein kinase C (PKC) family, sharing a high degree of homology within their C-terminal kinase domains (Mukai, 2003). The divergent N-terminal regulatory domains bear a C2 domain and three polybasic coiled-coil HR1 domains, which confer binding and regulation by Rho family members. While other mammalian PKCs lack HR1 domains and Rho responsiveness, PKC orthologs in yeast bear two HR1 domains and signal downstream of Rho1; yeast PKC also retains the regulatory C1 and C2 domains found in mammalian PKC isoforms, suggesting an evolutionary split between the Rho-responsive and the Rho-independent functions of the PKC family. There are three PKN family members in mammals (PKN1–PKN3, also known as PRKs), which vary in their regulation. While both PKN1 and PKN2 are activated by RhoA and Rac1 (Flynn et al., 1998; Vincent and Settleman, 1997), they respond differently to phosphoinositides and fatty acids (Mukai, 2003). PKN isoforms also respond to distinct stimuli and bind to distinct effectors, highlighting functional divergence within the family. Expression patterns also differ; PKN1 and PKN2 mRNAs are ubiquitous in expression, while PKN3 is more restricted to muscle, liver, and endothelial cells (Aleku et al., 2008; Palmer et al., 1995). In vivo, a role for PKN1 in germinal center formation has been reported (Yasui et al., 2012).

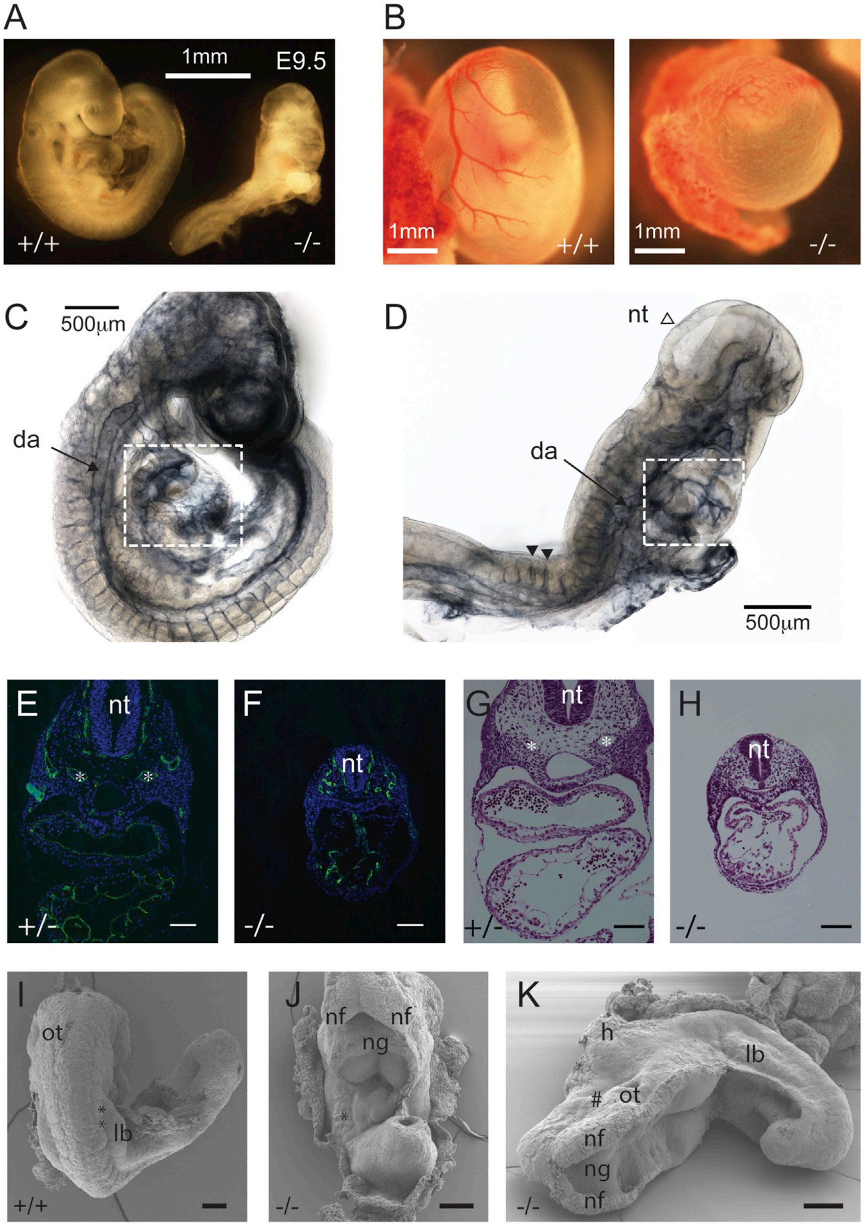

Figure 1. PKN2 Ablation Is Embryonic Lethal and Causes Growth, Morphogenetic, and Cardiovascular Defects

(A) PKN2$^{-/-}$ embryos are smaller than littermates, do not undergo axial turning, and exhibit no branchial arches.

(B) Yolk sacs from mutant embryos have no vitelline vessels and exhibit an immature vascular plexus.

(C and D) Whole-mount PECAM1 staining of E9.5 PKN2$^{+/-}$ and PKN2$^{-/-}$ embryos. KO embryos exhibit collapsed dorsal aorta and underdeveloped heart (boxed). The open neural tube example (open arrowhead) and intersomitic vessel examples (closed arrowheads) are indicated.

(E–H) Transverse sections through E9.5 embryos were subjected to endomucin staining (E and F) or H&E (G and H). Asterisks indicate dorsal aorta in control PKN2$^{+/-}$ embryos.

(I–K) Scanning electron microscopy of WT (+/+) and PKN2$^{-/-}$ E9.5 embryos indicating fully open neural tube in PKN2$^{-/-}$ embryos. Asterisks indicate somites; hash mark indicates under-developed head.

Da, dorsal aorta; nt, neural tube; ng, neural groove; nf, neural fold; ot, otic vesicle; lb, limb bud. Scale bars represent 100 μm unless stated.

Here, we report genetic ablation of all three mouse PKN isoforms to reveal a non-redundant essential role for PKN2 in the developing embryonic mesenchyme.

RESULTS

PKN2 Plays a Non-redundant Role in Development

The mouse PKN1 and PKN3 genes were targeted in embryonic stem cells (ESCs) by homologous recombination (Figure S1), and germline transmission resulted in fertile heterozygous mice. Gene disruption was assessed by RT-PCR and western blot (Figures S1D–S1G). PKN1 and PKN3 crosses generated Mendelian ratios of wild-type (WT), heterozygous, and knockout (KO) mice (Table S1). Mice were fertile and exhibited no overt phenotype. To assess redundancy, the PKN1 and PKN3 mice were interbred, which generated Mendelian numbers of healthy, fertile, double-KO mice (Table S1).

PKN2-targeted "KO-first" ESCs were obtained from the Knockout Mouse Project (KOMP) consortium (Skarnes et al., 2011). The KO-first strategy generates a null allele (PKN2^{tm1a}) but allows conversion to a conditional PKN2^{flox} allele through flp/FRT recombination (PKN2^{tm1c}; Figure S1). Germline transmission was achieved with two independent ESC clones, but crossing PKN2^{tm1a} heterozygotes (herein referred to as PKN2$^{+/-}$) from either ESC clone generated no homozygous PKN2 KO mice (Table S1). To confirm that this results from disruption of the PKN2 gene, the locus was flp/FRT recombined (PKN2^{tm1c}) by crossing with a Flp deleter mouse; recombination rescued expression of PKN2 and survival of homozygous offspring (Table S1). Finally, PKN2$^{+/-}$ mice crossed onto a PKN1/3 KO background were generated at the expected frequency with no overt phenotype (n = 10), indicating that a single allele of PKN2 is sufficient for embryogenesis.

PKN2 Loss Results in Embryonic Lethality by E10

Homozygous embryos derived from both independent ESC clones died at embryonic day 10 (E10; Table S1) and failed to undergo axial turning (Figure 1A). No PKN2 protein or truncated fragments were detectable in KO E8 embryos or yolk sacs, while

$PKN2^{+/-}$ embryos expressed half as much PKN2, confirming gene disruption (Figures S2A and S2B). Compressed somites (14–18 somite pairs) in E9.5 mutant embryos demonstrate a failure to elongate, consistent with defects in convergent extension. A lack of branchial arch formation and head development implied a lack of mesenchymal expansion (Figures 1A and 1K). The absence of vitelline vessels on the yolk sacs and pericardial edema indicated a cardiovascular or angiogenic defect (Figure 1B), and the neural tube remained open to varying degrees in all embryos (Figures 1D, 1J, and 1K). To exclude trophoblast or placental defects as the cause of lethality, we conducted tetraploid rescue experiments, which indicated that PKN2 is required in the embryo or yolk sac (Figure S2C).

Immunostaining indicated ubiquitous expression of PKN2 in E10 embryo frozen sections, with some accumulation at the apical surface of the neuroepithelium and in the ectoderm (Figure S2D). LacZ reporter expression (β-galactosidase) confirmed broad PKN2 expression (Figure S2E). qRT-PCR analysis of PKN1 and PKN3 expression levels in E8 embryos indicated no mRNA expression compensation in the PKN2 KOs (Figure S2F). PKN1 and PKN2 mRNAs were both readily detectable by RNAscope broadly throughout the embryo. While PKN3 protein could not be detected in embryo extracts, mRNA could be detected in endothelial cells (as previously reported) and at low levels in other embryo tissues (Figure S2G).

PKN2 Null Embryos Have Cardiovascular and Neural Tube Defects

Immunostaining and histology of yolk sacs revealed an immature vascular plexus (Figures S3A and S3B) and separation of the endodermal and mesodermal layers in PKN2 KOs (Figure S3A). These phenotypes are associated with both cardiac and angiogenic defects. Heart and vascular development are intrinsically linked processes, because hemodynamic force is necessary for vascular remodeling (Lucitti et al., 2007). Substantial numbers of primitive nucleated blood cells within the vasculature of PKN2 KO embryos, and beating hearts, indicated that circulation is established, typically occurring at E8.5–E9. Whole-mount staining of the vasculature indicated collapsed major vessels and sparse peripheral vasculature (Figures 1C and 1D). This was confirmed in transverse sections, where a disorganized endothelial network is apparent throughout the mesenchyme (Figures 1E and 1F). Histology also revealed a delay in cardiac looping, more typical of an embryonic heart at E8 (Figures 1G and 1H; Figure S3), further confirmed by staining E8.5 whole-mount and transverse sections of embryo hearts for desmin; 3D reconstructions revealed a delay in heart morphogenesis (Figures S3C and S3D).

PKN2 KO embryos exhibit a range of neural tube defects. Some prospective forebrain development in E9.5 embryos was observed, with closure of the forebrain neural fold to form a neural lumen, diencephalon, and optic vesicles (Figure S3E). The prospective hind-brain neural tube was always open, often with no discernible neural groove. Some embryos exhibit chranioraschischisis (Figures 1I–1K), a phenotype associated with planar cell polarity (PCP) mutants, such as looptail (Murdoch et al., 2001), which fail to undergo convergent extension.

Conditional $PKN2^{flox}$ Deletion in Either the Endothelium or the Heart Does Not Phenocopy the Embryonic Vascular Defect

To investigate an endothelial role, we crossed our conditional $PKN2^{flox}$ mouse ($PKN2^{tm1c}$; Figure S1) with the endothelial-specific Tie2-Cre (Koni et al., 2001). Tie2-Cre has been used to uncover embryonic endothelial dependencies displaying phenotypes similar to our PKN2 KO, including phosphatidylinositol 3-kinase p110α (Graupera et al., 2008). In contrast to global PKN2 KO, Tie2-Cre $PKN2^{fl/fl}$ mice were born at Mendelian frequency and displayed no overt phenotype (Table S2). An analysis of adult mouse retinal vasculature also revealed no discernible angiogenic differences in Tie2-Cre $PKN2^{fl/fl}$ mice (Figures 2A–2D). Deletion was confirmed by immunoblot of mouse lung endothelial cell lysates (Figure 2E). We conclude that PKN2 is largely dispensable in endothelial cells. Although Tie2 is expressed in all endothelial cells (Schlaeger et al., 1997), a role for PKN2 in endothelial precursors before Tie2-Cre recombination cannot be entirely excluded.

Mutants with severe cardiac defects, such as the contraction-deficient MLC2a deletion (Huang et al., 2003) exhibit embryonic vascular defects and die mid-gestation, indicating the need for hemodynamic force in vascular remodeling (Lucitti et al., 2007). To delete PKN2 in the embryonic heart, we crossed $PKN2^{flox}$ mice with SM22α-Cre, which is expressed in the developing heart tube (from E7.5) and smooth muscle cells (Lepore et al., 2005). SM22α-Cre-mediated PKN2 deletion shows a partially penetrant lethal phenotype (Table S2). Surviving mice were fertile, but pathology revealed cardiac hypertrophy, lung fibrosis, and alveolar enlargement. To examine effects on heart development, we collected several sets of embryos (E9.5–E13.5). Embryo numbers were Mendelian, and KOs were indistinguishable from littermates (Table S2). Transverse sections revealed no gross differences in heart size or cardiomyocyte bromodeoxyuridine (BrdU) incorporation (Figures 2F and 2G). Smooth muscle actin (SMA) staining also demonstrated comparable smooth muscle staining of the dorsal aorta. Recombination was confirmed by loss of PKN2 protein in adult heart ventricle extracts (Figure 2H), and robust Cre expression throughout the E9.5 embryo heart was confirmed by crossing with the Rosa mTmG reporter mouse (Figure 2I). Although SM22 partial lethality and adult pathology indicate an unexplored role for PKN2 in the heart or smooth muscle cells, the survival of some offspring, and the near-normal embryonic development, implies that PKN2 is not essential for early cardiovascular function.

Inducible Deletion of PKN2 In Vivo Results in Mesenchymal Collapse

Next, we examined the temporal requirements for PKN2 by crossing with Rosa26 Cre-ERT2 mice (iCre; Taconic Biosciences), which allows for systemic tamoxifen-induced regulation of Cre activity in vivo. We crossed homozygous iCre $PKN2^{fl/fl}$ mice with $PKN2^{fl/+}$ mice to generate litters with a 1:1 ratio of iCre (heterozygous) $PKN2^{fl/fl}$:$PKN2^{fl/+}$. Because $PKN2^{+/-}$ embryos are indistinguishable from WT embryos, iCre $PKN2^{fl/+}$ act as controls. A single dose of tamoxifen (3 mg oral gavage) induced loss of PKN2 within 48 hr (Figure 2J). Tamoxifen administration at E7.5 was sufficient to recapitulate the axial turning defects in most

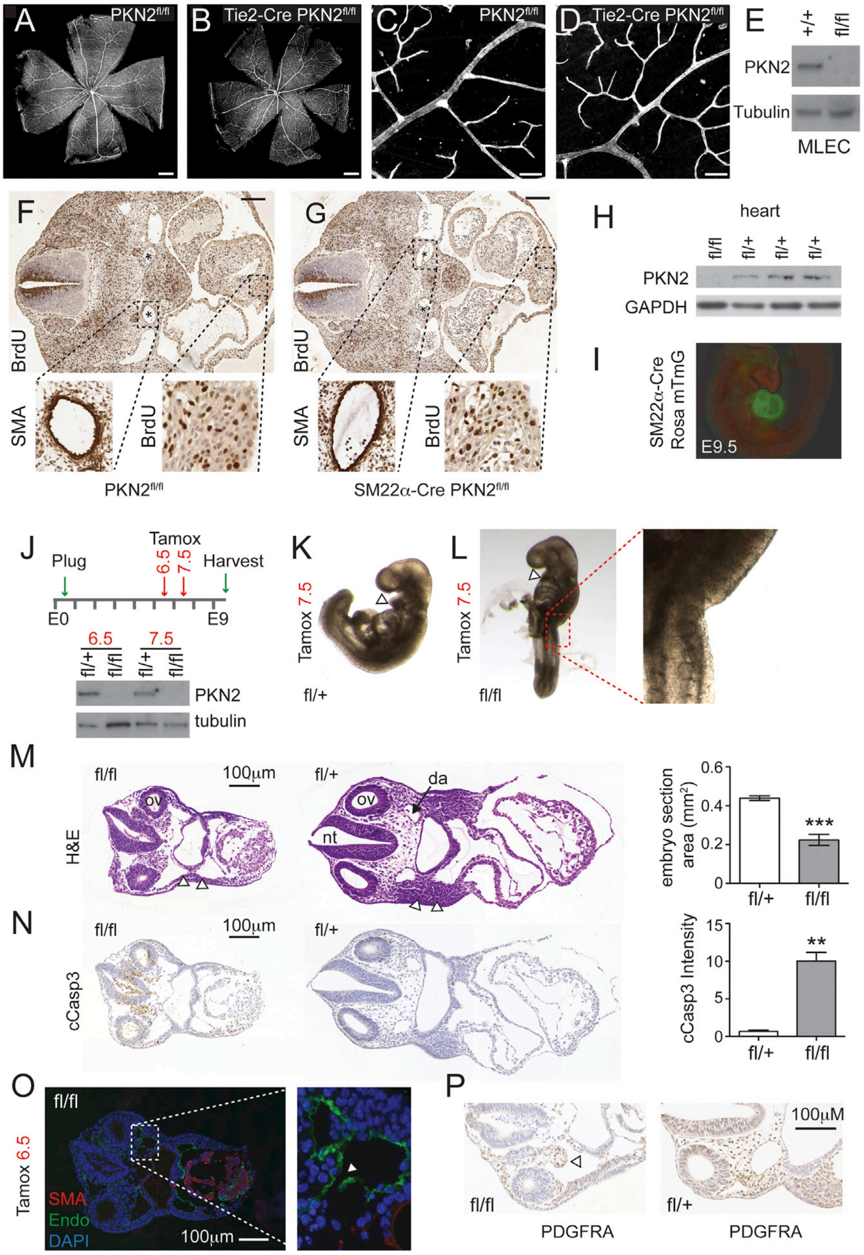

Figure 2. Inducible Deletion at E6.5, but Not Tie2-Cre or SM22α-Cre, Phenocopies the Constitutive Knockout

(A–D) Retinal vasculature was stained for isolectin B. Scale bars represent 500 μm (A and B) and 50 μm (C and D).

(E) Western blots indicate that endothelial PKN2 is deleted in Tie2-Cre PKN2$^{fl/fl}$ mice.

(F and G) Transverse sections through E11.5 embryos reveal normal heart and major vessel development in SM22α-Cre PKN2$^{fl/fl}$ embryos. Scale bars represent 200 μm. Heart ventricle proliferation (BrdU) and SMA staining of the dorsal aorta (*) were comparable.

(H) Western blots indicate PKN2 deletion in adult heart tissue from SM22α-Cre PKN2$^{fl/fl}$ mice.

(I) SM22α-Cre recombination in hearts is robust at E9.5, as assessed by the Rosa mTmG reporter.

(J) Tamoxifen was given on day 6 or 7 of pregnancy before embryo harvest at E9.5; PKN2 protein was lost in PKN2$^{fl/fl}$ embryos at E9.5 (bottom panels).

(K and L) Deletion of PKN2 at E7.5 induced axial turning defects in iCre PKN2$^{fl/fl}$ embryos.

(M and N) Transverse sections from E9.5 embryos following E6.5 deletion were stained for H&E and cleaved caspase 3. Littermate embryo cross sectional area and caspase 3 pixel intensity were measured using panoramic viewer software; data are mean ± SD (n = 3). Open arrowheads indicate neural crest-derived branchial arches, absent following PKN2 deletion (M). Sections show the otic vesicles (ov), neural tube (nt), and dorsal aorta (da). Brightfield slide images were acquired on a Pannoramic 250 slide scanner (3Dhistech) and may comprise multiple instrument merged panels.

(O) Endomucin and SMA staining revealed collapsing vessels (arrowhead) and an unlooped heart.

(P) PDGFRA staining confirmed mesenchymal regression.

(7/10) E9.5 PKN2$^{fl/fl}$ embryos (Figure 2L). Tamoxifen administration at E6.5 results in recapitulation of the gross phenotypes of the full PKN2 KO (Figures 2M–2O). PKN2$^{fl/fl}$ embryos also exhibit collapse of the mesenchyme, apoptosis, vascular disintegration (Figures 2M–2P), and loss of branchial arches (Figures 2K–2M). Together, our conditional KOs support a model in which the heart and vascular defects arise secondary to a deficit within the mesodermal and neural crest-derived mesenchyme.

PKN2-Deficient MEFs Exhibit Proliferative and Migratory Defects

It was not possible to derive mouse embryonic fibroblasts (MEFs)—which derive from the embryonic mesenchyme—from PKN2$^{-/-}$ embryos; MEFs failed to proliferate post-extraction in contrast to littermate WT and PKN2$^{+/-}$ controls. To examine the cell biological role of PKN2, we instead derived MEFs from the iCre PKN2$^{fl/fl}$ embryos and sex-matched littermate iCre PKN2$^{+/+}$ controls. iCre PKN2$^{+/+}$ MEFs allow for control for nonspecific toxicity of 4-hydroxytamoxifen (4-OHT) or Cre induction. Treatment of iCre PKN2$^{fl/fl}$ MEFs with 100 nM 4-OHT for 1 hr is sufficient to induce PKN2 recombination, as observed by loss of PKN2 after 48 hr (Figure 3A). PKN2 deletion attenuated MEF growth (Figures 3B and 3C), in line with the failure of PKN2 null MEFs to proliferate. No decrease in cell growth was observed for control iCre PKN2$^{+/+}$ cells, and comparable results were observed for multiple iCre PKN2$^{fl/fl}$ MEF lines. Growth dependence is cell type specific, because deletion of PKN2 protein (data not shown) from iCre ESCs had no effect on growth (Figure 3B), as expected given the normal cellular expansion during early embryogenesis.

OPEN
ACCESS
CellPress

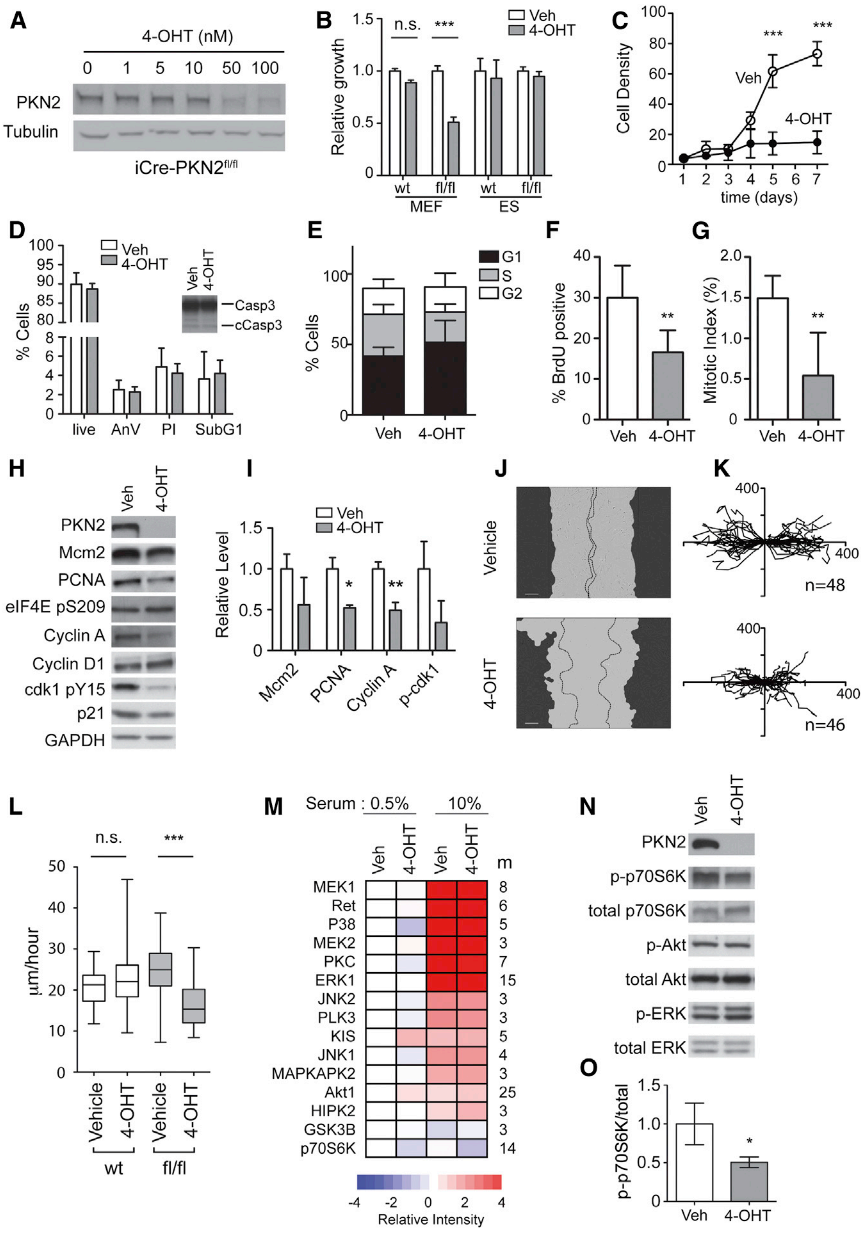

Figure 3. PKN2 Deletion Results in Reduced Proliferation and Migration of MEFs

(A) 4-OHT treatment of iCre-PKN2 MEFs results in dose-dependent loss of PKN2.

(B and C) 4-OHT induction results in reduced cell viability (B) and slower growth (C) for iCre PKN2$^{fl/fl}$ but not iCre PKN2 WT cells (B; n = 5; ***p < 0.001). (B) ESC viability is not decreased by PKN2 deletion.

(D) No increase in annexin V binding, propidium iodide uptake, sub-G1 cell fraction, or cleaved caspase 3 (cCasp3; inset western blot) indicates no increase in cell death following PKN2 deletion.

(E–G) Cell-cycle analysis, BrdU uptake, and p-Histone H3 binding indicate accumulation in the G1/G0 fraction and loss of S-phase and mitotic cells.

(H and I) PKN2 deletion caused loss of cell-cycle proteins. Blots were quantified relative to GAPDH; data are mean ± SD (n = 3; p < 0.05, **p < 0.01).

(J) 4-OHT Cre induction results in slower scratch wound closure for iCre PKN2$^{fl/fl}$ but not iCre PKN2 WT cells. Mask indicates starting cell front, and dotted lines indicate cell front after 16 hr of migration.

(K and L) Single-cell migration speed is reduced; cell tracks (K; axes indicate micrometers) and the speed over the 16 hr time course are displayed (L). Box and whiskers indicate the average, quartiles, and range (***p < 0.001 ANOVA).

(M) Quantitative mass spectrometry (MS) analysis and KSEA indicate broadly comparable serum-induced activation of major signaling pathways following PKN2 deletion. MEFs were serum starved (0.5%) and re-stimulated (10%) for 15 min before analysis by LC-MS/MS (n = 4). Pathway activation (m = number of kinase substrate peptides quantified) relative to control extracts is presented as a heatmap.

(N and O) Akt, ERK, and p70S6K activation levels in control and PKN2-depleted cells were assessed by western blot and quantified by densitometry; data are mean ± SD (n = 3; *p < 0.05).

PKN2 loss did not result in cell death in MEFs; no increase in annexin V staining, propidium iodide uptake, sub-G1 cells, or cleaved caspase 3 was observed following PKN2 deletion (Figure 3D), and non-proliferating cells remained viable through multiple passages. This suggests that cell death observed in the embryo is likely secondary to the death of the embryo.

PKN2 has been reported to regulate mitotic entry and cytokinesis in HeLa cells (Schmidt et al., 2007). In contrast, deletion of PKN2 in MEFs was associated with accumulation of cells in G1/G0 with an accompanied loss of S-phase and mitotic cells, as assessed by BrdU incorporation, phospho-Histone (p-Histone) H3 staining, and cell-cycle analysis (Figures 3E–3G). Decreased expression levels of cyclin A, PCNA, and Mcm2 were consistently observed, as well as decreased levels of phosphorylated Cdk1 (Figures 3H and 3I).

We reported a specific non-redundant role for PKN2 in cancer cell migration (Lachmann et al., 2011). Comparably, scratch wound closure rate and single-cell migration speed were reduced following PKN2$^{fl/fl}$ deletion, while control iCre PKN2$^{+/+}$ MEF migration was unaffected (Figures 3J–3L). The actin cytoskeleton, however, was largely unchanged by PKN2 deletion (Figure S4A).

To gain an unbiased view of PKN2 signaling, we conducted quantitative phosphoproteomic analysis. MEFs ± PKN2 were serum starved overnight and re-stimulated with serum for 15 min before analysis by liquid chromatography-tandem mass spectrometry (LC-MS/MS). In excess of 6,000 individual phosphopeptides were quantified across four biological replicates. Ontological analysis identified numerous phosphopeptides

derived from proteins associated with cell cycle, S phase, and mitosis that were significantly depleted in response to PKN2 loss (Table S3). These findings corroborate cell-cycle withdrawal following PKN2 loss. We also found enrichment for phosphopeptides associated with cytoskeletal organization (Table S4), in line with the decreased cell motility observed here and the known functions of the PKN kinases.

Phosphoproteomic pathway mapping through kinase substrate enrichment analysis (KSEA; Casado et al., 2013; Rajeeve et al., 2014) allows assessment of signal transduction by linking phosphosites with their known kinases to determine pathway activation. KSEA revealed that PKN2 loss is associated with minimal changes in serum-induced activation of proliferative signaling (Figures 3M and 3N). The Akt pathway was marginally elevated under serum-starved conditions following PKN2 deletion, although serum-activated Akt levels were comparable. KSEA identified partial suppression of the p70S6K pathway. These findings were confirmed by immunoblot, where active p70S6K was reduced (Figures 3N and 3O), while active ERK and Akt in serum were unchanged. This indicates that serum growth factor coupling to key proliferative signaling axes remains largely intact upon PKN2 deletion and identifies p70S6K as a potential PKN2 effector pathway.

PKN2 Loss Results in Reduced Proliferation of the Paraxial Mesoderm and Reduced Neural Crest Migration In Vivo

To corroborate the observed mesenchymal cell biology defects, we conducted a series of induced deletions in the iCre PKN2 mouse. Staining of E10 embryos 48 hr after tamoxifen administration allowed quantification of mitosis and apoptosis within distinct tissues. Pharyngeal mesoderm displayed a significant decrease in mitotic cells in PKN2$^{fl/fl}$ embryos when compared with littermate PKN2$^{fl/+}$ controls (Figures 4A and 4C). In contrast, the branchial arch, neural tube, and heart mitotic index remained comparable. Apoptosis in the same tissues was variable between embryos and non-tissue specific (Figures 4B and 4D). These embryos exhibited a deficit of cephalic mesoderm and display evidence of hemorrhage (Figure 4E). Together, these observations support a model in which failure of the mesoderm to proliferate results in loss of support for vascular development and embryo death. This conclusion is reinforced by the extensive apoptosis observed following longer periods of PKN2 deletion (Figure 2N; data not shown).

Global PKN2 KO or induced deletion before E7 results in loss of branchial arches, which derive from both the cranial neural crest and the cranial mesoderm (Noden and Trainor, 2005). Neural crest cells (NCCs) delaminate from the neuroendothelium and migrate ventrally through the mesoderm. We conducted in situ hybridization for erbB3 in whole-mount embryos, which labels migrating NCCs (Britsch et al., 1998). In PKN2 KO embryos, NCCs remain close to where they delaminate instead of migrating ventrally through the mesoderm, as in WT controls. In severe cases, in which embryos exhibit craniorachischisis, NCCs, where detectable, do not migrate (Figure 4F). These data implicate PKN2 in the development and migration of NCCs in vivo.

In summary, our findings support a mechanism by which mesodermal and neural crest lineages acquire dependence on PKN2 for growth and migration mid-gestation. Failure of these lineages to expand and migrate results in loss of the physical support required for cardiovascular integrity and development. The critical contribution of pharyngeal mesoderm and NCCs to the secondary heart field is also likely to contribute to the retarded unlooped hearts in PKN2 null embryos (Plein et al., 2015; Tzahor and Evans, 2011).

DISCUSSION

PKN kinases are emerging as regulators of cancer growth, invasion, and metastasis. Despite this, and in contrast to other Rho-activated kinases, little is known about their in vivo physiological roles. By targeting the PKN family in vivo, we have uncovered a non-redundant role for PKN2 during development. Here we establish key roles in morphogenesis, lineage-specific growth, migration, and support of cardiovascular development.

Failure to establish a circulatory system in the embryo results in death at mid-gestation (Copp, 1995), the point at which PKN2-null embryos die. Because this process requires interplay among numerous co-dependent systems, unraveling cause and effect can be challenging. Many of the genes identified as essential for establishing circulation are those involved in heart development and endothelial cell function; conditional KOs suggest that PKN2 is largely dispensable here. In contrast, induced loss of PKN2 mid-gestation resulted in collapse of the embryonic paraxial mesoderm, essential for vascular support and heart development (Tzahor and Evans, 2011).

A role for PKN2 in the mesoderm was supported by experiments with mesenchymal-derived MEFs, which depend on PKN2 for growth and motility. In HeLa cells PKN2 regulates mitosis (Schmidt et al., 2007), and in starfish oocytes PKN2 can directly phosphorylate (Ser209), and regulate, the key S-phase initiator, eIF4E (Lee et al., 2000). In contrast, here, in a physiological context, PKN2 loss was associated with arrest in G1/G0, a decrease in S-phase and mitotic cells, and no change in eIF4E phosphorylation (Ser209) or downstream cyclin D1 expression.

PKN2 loss also results in impaired NCC migration in vivo and impaired MEF cell motility in vitro, in concordance with embryo morphogenetic defects. We previously reported a non-redundant role for PKN2 in bladder cancer cell migration (Lachmann et al., 2011), perhaps shedding light on PKN2 isoform specificity. Functionally, the actin cytoskeleton was largely unaffected by PKN2 loss in MEFs, suggesting that RhoA regulation of actin stress fibers is functional. Likewise, F-actin and Rac1 localized independently of PKN2 at the leading edge of migrating cells (Figure S4A). We did, however, identify modulation of many cytoskeletal components in our phosphoproteomics following PKN2 loss (Figure S4), and detailed studies on cytoskeletal dynamics are clearly warranted.

Among the well-described pathways regulating growth and migration in the developing embryo is the lysophosphatidic acid (LPA) axis, where mutants (e.g., autotaxin and Gα12/13) largely phenocopy loss of PKN2, with vascular, neural tube, and axial turning defects (Tanaka et al., 2006; van Meeteren et al., 2006). In support of a role in LPA signaling, deletion of PKN2 from early-passage MEFs was found to blunt LPA-induced

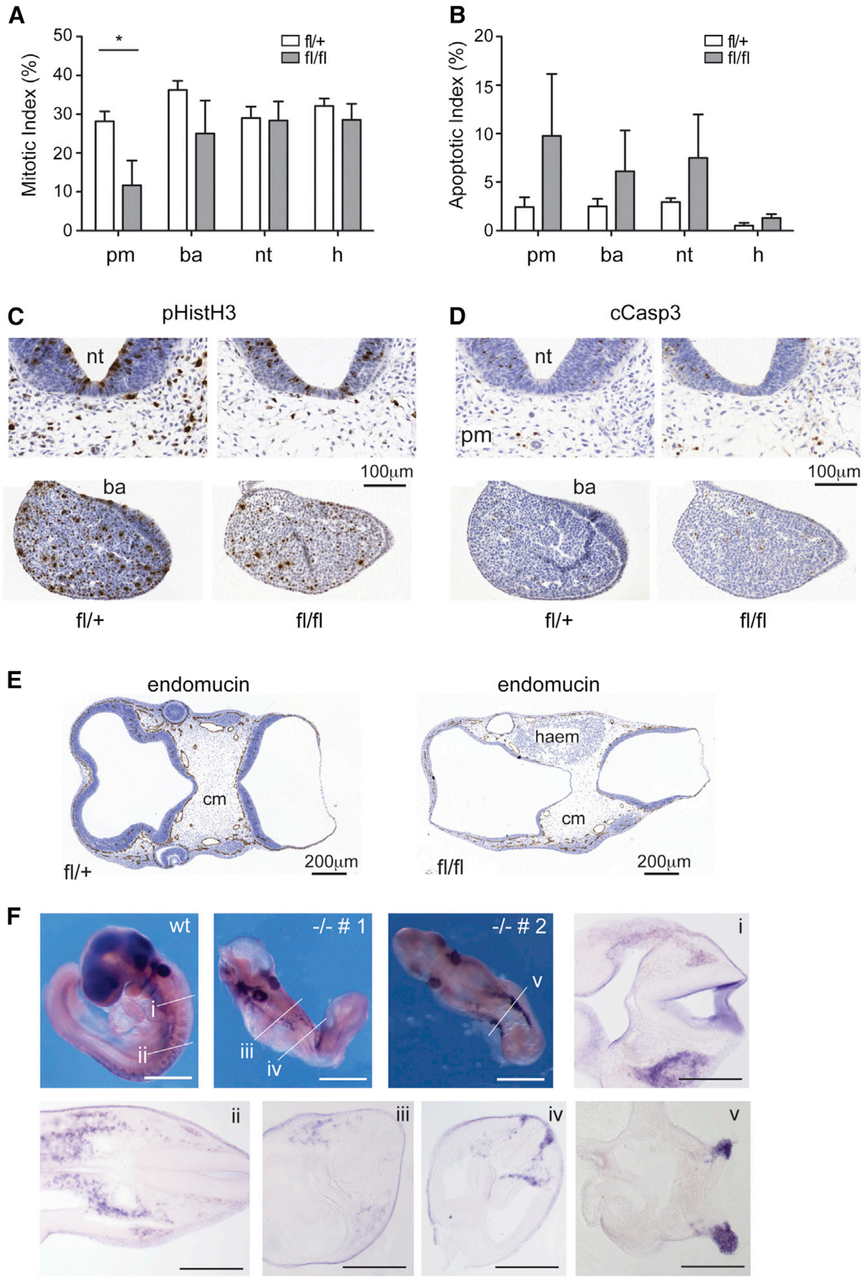

Figure 4. Induced Deletion of PKN2 In Vivo Decreases Mesodermal Proliferation and NCC Migration

(A–E) Tamoxifen was given on day 8 of pregnancy before embryo harvest at E10. Embryos were sectioned and stained for p-Histone H3 (pHistH3) to measure mitotic index and cleaved caspase 3 (cCasp3) to measure apoptotic index. (A) Aligned sections were counted to reveal a significant decrease in mitotic index in the pharyngeal mesoderm (pm) but not the branchial arches (ba), neural tube (nt), or heart (h) following PKN2 deletion in PKN2$^{fl/fl}$ but not PKN2$^{fl/+}$ embryos (n = 3; *$p < 0.05$). (B) Apoptosis was variably increased across all tissues but did not reach significance. (C and D) Examples of pHistH3 and cCasp3 section stains are displayed. (E) PKN2 loss results in collapse of the cephalic mesoderm (cm) as evidenced by hemorrhage (haem) and loss of cellularity.

(F) Whole-mount in situ staining reveals a deficit of migrating NCCs in PKN2 KO embryos. A single-WT and two KO embryos are presented; −/− # 1 has a partially closed neural tube, whereas −/− # 2 exhibits craniorachischisis. Vibratome sections reveal a deficit of NCCs and reduced ventral migration. Scale bars represent 100 μm.

migration (Figure S4). However, no differences in LPA-induced actin rearrangement, proliferative signaling, or transcription (CTGF or CYR61) were observed. Thus, while PKN2 may play some role, many aspects of LPA function remain intact.

The Rho GTPases are established regulators of convergent extension and neural tube formation, with focus on the Wnt/PCP pathway (Schlessinger et al., 2009). PCP pathway mutants exhibit craniorachischisis, a severe dorsal neural tube defect (Harris and Juriloff, 1999) also observed in PKN2$^{-/-}$ embryos. Closure of the forebrain neural fold, observed for our PKN2 KO, is further consistent with PCP mutants such as looptail/vangl2 (Copp et al., 1994). Apical accumulation of Rho as a PCP effector has been reported in neural plate morphogenesis in the chick embryo (Kinoshita et al., 2008), and PKN2 is present in this compartment (Figure S2D). Intriguingly, ROCK has been implicated as a key Rho effector here in both the mouse (Escuin et al., 2015; Ybot-Gonzalez et al., 2007) and the chick (Kinoshita et al., 2008) because of sensitivity to the ROCK inhibitor Y27632. However, our phenotypic observations, coupled with the reported sensitivity of PKN2 to Y27632 inhibition (Davies et al., 2000; Falk et al., 2014), place PKN2 as a potentially important Rho effector kinase in this context.

From an evolutionary perspective, *Drosophila* has a single PKN ortholog (dPkn), which is essential during embryogenesis, with mutant flies exhibiting dorsal closure failure (Lu and Settleman, 1999). In flies, dorsal closure occurs via a mechanism apparently requiring both contraction of the underlying amnioserosa and migration of the overlying epithelial layers (Jacinto et al., 2002). Considering the parallels between *Drosophila* dorsal closure and mammalian neural tube closure, it is tempting to speculate that PKN2 acts as the mammalian ortholog to dPkn in this context.

The genetic ablation of the PKN family reported here completes the description of KOs for the entire PKC superfamily in mice and reveals that PKN2 is one of only two family members essential for viability; atypical PKCλ (PKCι in *Homo sapiens*) KOs also die mid-gestation with gross morphological abnormalities (Soloff et al.,

2004). The finding that mammalian PKN2 is essential also unifies the requirement for Rho family-responsive PKC activity from yeast to man and highlights a surprising degree of isoform selectivity within the PKN family.

EXPERIMENTAL PROCEDURES

Mice

Studies in animals were approved by the Animal Ethics Committee of the London Research Institute (now the Francis Crick Institute) and the UK Home Office. PKN1 and PKN3 KO mice were generated by homologous recombination, and PKN2-targeted KO-first ESCs were purchased from KOMP consortium. Tie2-Cre and SM22α-Cre were provided by Taija Makinen and Ralf Adams. Rosa26CreERT2 mice (Gt(ROSA)26Sortm9(cre/ESR1)Arte) are from Taconic Biosciences. All mice were backcrossed to a C57BL/6J background.

Pathology and Immunostaining

Embryos and tissues were stained according to standard techniques. For PKN1, PKN2, and PKN3 in situ staining, an RNAscope 2.0 formalin fixed paraffin embedded assay kit was used (Advanced Cell Diagnostics) according to the manufacturer's instructions. Whole-mount ErbB3 in situ staining was carried out as described elsewhere (Pryor et al., 2014). Images were acquired on a 3DHISTECH slide scanner, Nikon Eclipse, or Zeiss 710 confocal microscope.

Cell Biology and Proteomics

MEF and ESC lines were derived according to standard protocols (see the Supplemental Information). Cell growth and viability were assessed by cell counting and MTT (3-(4,5-dimethylthiazol-2-yl)-2,5-diphenyltetrazolium bromide). For immunoblots, proteins were resolved by SDS-PAGE, transferred to nitrocellulose, and incubated with appropriate antibodies before enhanced chemiluminescence visualization. For cell cycle (propidium iodide), BrdU incorporation, and mitosis, cells were fixed in ice-cold 70% ethanol, stained with appropriate antibodies, and assessed by fluorescence-activated cell sorting. Migration was followed by time lapse (Essen IncuCyte). For RT-PCR, RNA (QIAGEN) was transcribed to cDNA and amplified with TaqMan RT and Sybr Green PCR Mix (Applied Biosystems). Quantification and analysis of phosphopeptides was performed by LC-MS/MS (Casado et al., 2013; Rajeeve et al., 2014).

Statistical Methods

For two-group comparisons, statistical significance was assessed using paired (cell cycle) or unpaired (viability, embryo quantifications, and protein expression or phosphorylation) Student's t tests; for multiple comparisons (growth curves and migration), statistical significance was assessed by ANOVA (GraphPad Prism). For phosphoproteomics, statistical significance across four independent biological replicates was assessed with a Benjamini and Hochberg adjusted p value < 0.05.

Full details are in the Supplemental Experimental Procedures.

SUPPLEMENTAL INFORMATION

Supplemental Information includes Supplemental Experimental Procedures, four figures, and four tables and can be found with this article online at http://dx.doi.org/10.1016/j.celrep.2015.12.049.

AUTHOR CONTRIBUTIONS

A.J.M.C. and P.J.P. conceived the project and wrote the manuscript. I.Q., A.J.M.C., and J.J.T.M. conducted the majority of the experiments. S.L., A.C., A.J.M.C., and I.R. derived the KO mice. C.F. and H.G. conducted cardiovascular studies. M.H., P.B., and J.T.W. assisted with cell migration. S.E. and A.J.C. conducted neural crest analysis. B.S.-D. conducted dissections and pathology with G.S. V.R. and P.C. contributed the MS analysis.

ACKNOWLEDGMENTS

We thank Cancer Research UK, Barts Cancer Institute, HEFCE, the Wellcome Trust (087525 to A.J.C.), and the Royal Society for funding; Lucy Collinson and Electron Microscopy for imaging; the Crick BRF staff for animal studies; and Natalia Kazakova for ESC help.

Received: April 15, 2015
Revised: November 6, 2015
Accepted: December 7, 2015
Published: January 7, 2016

REFERENCES

Aleku, M., Schulz, P., Keil, O., Santel, A., Schaeper, U., Dieckhoff, B., Janke, O., Endruschat, J., Durieux, B., Röder, N., et al. (2008). Atu027, a liposomal small interfering RNA formulation targeting protein kinase N3, inhibits cancer progression. Cancer Res. *68*, 9788–9798.

Britsch, S., Li, L., Kirchhoff, S., Theuring, F., Brinkmann, V., Birchmeier, C., and Riethmacher, D. (1998). The ErbB2 and ErbB3 receptors and their ligand, neuregulin-1, are essential for development of the sympathetic nervous system. Genes Dev. *12*, 1825–1836.

Bustelo, X.R., Sauzeau, V., and Berenjeno, I.M. (2007). GTP-binding proteins of the Rho/Rac family: regulation, effectors and functions in vivo. BioEssays *29*, 356–370.

Casado, P., Rodriguez-Prados, J.C., Cosulich, S.C., Guichard, S., Vanhaesebroeck, B., Joel, S., and Cutillas, P.R. (2013). Kinase-substrate enrichment analysis provides insights into the heterogeneity of signaling pathway activation in leukemia cells. Sci. Signal. *6*, rs6.

Copp, A.J. (1995). Death before birth: clues from gene knockouts and mutations. Trends Genet. *11*, 87–93.

Copp, A.J., Checiu, I., and Henson, J.N. (1994). Developmental basis of severe neural tube defects in the loop-tail (Lp) mutant mouse: use of microsatellite DNA markers to identify embryonic genotype. Dev. Biol. *165*, 20–29.

D'Amico, G., Jones, D.T., Nye, E., Sapienza, K., Ramjuan, A.R., Reynolds, L.E., Robinson, S.D., Kostourou, V., Martinez, D., Aubyn, D., et al. (2009). Regulation of lymphatic-blood vessel separation by endothelial Rac1. Development *136*, 4043–4053.

Davies, S.P., Reddy, H., Caivano, M., and Cohen, P. (2000). Specificity and mechanism of action of some commonly used protein kinase inhibitors. Biochem. J. *351*, 95–105.

Escuin, S., Vernay, B., Savery, D., Gurniak, C.B., Witke, W., Greene, N.D., and Copp, A.J. (2015). Rho-kinase-dependent actin turnover and actomyosin disassembly are necessary for mouse spinal neural tube closure. J. Cell Sci. *128*, 2468–2481.

Falk, M.D., Liu, W., Bolaños, B., Unsal-Kacmaz, K., Klippel, A., Grant, S., Brooun, A., and Timofeevski, S. (2014). Enzyme kinetics and distinct modulation of the protein kinase N family of kinases by lipid activators and small molecule inhibitors. Biosci. Rep. *34*, 93–106.

Flynn, P., Mellor, H., Palmer, R., Panayotou, G., and Parker, P.J. (1998). Multiple interactions of PRK1 with RhoA. Functional assignment of the Hr1 repeat motif. J. Biol. Chem. *273*, 2698–2705.

Graupera, M., Guillermet-Guibert, J., Foukas, L.C., Phng, L.K., Cain, R.J., Salpekar, A., Pearce, W., Meek, S., Millan, J., Cutillas, P.R., et al. (2008). Angiogenesis selectively requires the p110alpha isoform of PI3K to control endothelial cell migration. Nature *453*, 662–666.

Harris, M.J., and Juriloff, D.M. (1999). Mini-review: toward understanding mechanisms of genetic neural tube defects in mice. Teratology *60*, 292–305.

Huang, C., Sheikh, F., Hollander, M., Cai, C., Becker, D., Chu, P.H., Evans, S., and Chen, J. (2003). Embryonic atrial function is essential for mouse embryogenesis, cardiac morphogenesis and angiogenesis. Development *130*, 6111–6119.

Jacinto, A., Woolner, S., and Martin, P. (2002). Dynamic analysis of dorsal closure in *Drosophila*: from genetics to cell biology. Dev. Cell *3*, 9–19.

Kinoshita, N., Sasai, N., Misaki, K., and Yonemura, S. (2008). Apical accumulation of Rho in the neural plate is important for neural plate cell shape change and neural tube formation. Mol. Biol. Cell *19*, 2289–2299.

Koni, P.A., Joshi, S.K., Temann, U.A., Olson, D., Burkly, L., and Flavell, R.A. (2001). Conditional vascular cell adhesion molecule 1 deletion in mice: impaired lymphocyte migration to bone marrow. J. Exp. Med. *193*, 741–754.

Lachmann, S., Jevons, A., De Rycker, M., Casamassima, A., Radtke, S., Collazos, A., and Parker, P.J. (2011). Regulatory domain selectivity in the cell-type specific PKN-dependence of cell migration. PLoS ONE *6*, e21732.

Lee, S.J., Stapleton, G., Greene, J.H., and Hille, M.B. (2000). Protein kinase C-related kinase 2 phosphorylates the protein synthesis initiation factor eIF4E in starfish oocytes. Dev. Biol. *228*, 166–180.

Lepore, J.J., Cheng, L., Min Lu, M., Mericko, P.A., Morrisey, E.E., and Parmacek, M.S. (2005). High-efficiency somatic mutagenesis in smooth muscle cells and cardiac myocytes in SM22alpha-Cre transgenic mice. Genesis *41*, 179–184.

Lu, Y., and Settleman, J. (1999). The *Drosophila* Pkn protein kinase is a Rho/Rac effector target required for dorsal closure during embryogenesis. Genes Dev. *13*, 1168–1180.

Lucitti, J.L., Jones, E.A., Huang, C., Chen, J., Fraser, S.E., and Dickinson, M.E. (2007). Vascular remodeling of the mouse yolk sac requires hemodynamic force. Development *134*, 3317–3326.

Mukai, H. (2003). The structure and function of PKN, a protein kinase having a catalytic domain homologous to that of PKC. J. Biochem. *133*, 17–27.

Murdoch, J.N., Doudney, K., Paternotte, C., Copp, A.J., and Stanier, P. (2001). Severe neural tube defects in the loop-tail mouse result from mutation of Lpp1, a novel gene involved in floor plate specification. Hum. Mol. Genet. *10*, 2593–2601.

Noden, D.M., and Trainor, P.A. (2005). Relations and interactions between cranial mesoderm and neural crest populations. J. Anat. *207*, 575–601.

Palmer, R.H., Ridden, J., and Parker, P.J. (1995). Cloning and expression patterns of two members of a novel protein-kinase-C-related kinase family. Eur. J. Biochem. *227*, 344–351.

Plein, A., Fantin, A., and Ruhrberg, C. (2015). Neural crest cells in cardiovascular development. Curr. Top. Dev. Biol. *111*, 183–200.

Pryor, S.E., Massa, V., Savery, D., Andre, P., Yang, Y., Greene, N.D., and Copp, A.J. (2014). Vangl-dependent planar cell polarity signalling is not required for neural crest migration in mammals. Development *141*, 3153–3158.

Rajeeve, V., Vendrell, I., Wilkes, E., Torbett, N., and Cutillas, P.R. (2014). Cross-species proteomics reveals specific modulation of signaling in cancer and stromal cells by phosphoinositide 3-kinase (PI3K) inhibitors. Mol. Cell. Proteomics *13*, 1457–1470.

Schlaeger, T.M., Bartunkova, S., Lawitts, J.A., Teichmann, G., Risau, W., Deutsch, U., and Sato, T.N. (1997). Uniform vascular-endothelial-cell-specific gene expression in both embryonic and adult transgenic mice. Proc. Natl. Acad. Sci. USA *94*, 3058–3063.

Schlessinger, K., Hall, A., and Tolwinski, N. (2009). Wnt signaling pathways meet Rho GTPases. Genes Dev. *23*, 265–277.

Schmidt, A., Durgan, J., Magalhaes, A., and Hall, A. (2007). Rho GTPases regulate PRK2/PKN2 to control entry into mitosis and exit from cytokinesis. EMBO J. *26*, 1624–1636.

Skarnes, W.C., Rosen, B., West, A.P., Koutsourakis, M., Bushell, W., Iyer, V., Mujica, A.O., Thomas, M., Harrow, J., Cox, T., et al. (2011). A conditional knockout resource for the genome-wide study of mouse gene function. Nature *474*, 337–342.

Soloff, R.S., Katayama, C., Lin, M.Y., Feramisco, J.R., and Hedrick, S.M. (2004). Targeted deletion of protein kinase C lambda reveals a distribution of functions between the two atypical protein kinase C isoforms. J. Immunol. *173*, 3250–3260.

Tanaka, M., Okudaira, S., Kishi, Y., Ohkawa, R., Iseki, S., Ota, M., Noji, S., Yatomi, Y., Aoki, J., and Arai, H. (2006). Autotaxin stabilizes blood vessels and is required for embryonic vasculature by producing lysophosphatidic acid. J. Biol. Chem. *281*, 25822–25830.

Tzahor, E., and Evans, S.M. (2011). Pharyngeal mesoderm development during embryogenesis: implications for both heart and head myogenesis. Cardiovasc. Res. *91*, 196–202.

van Meeteren, L.A., Ruurs, P., Stortelers, C., Bouwman, P., van Rooijen, M.A., Pradère, J.P., Pettit, T.R., Wakelam, M.J., Saulnier-Blache, J.S., Mummery, C.L., et al. (2006). Autotaxin, a secreted lysophospholipase D, is essential for blood vessel formation during development. Mol. Cell. Biol. *26*, 5015–5022.

Vincent, S., and Settleman, J. (1997). The PRK2 kinase is a potential effector target of both Rho and Rac GTPases and regulates actin cytoskeletal organization. Mol. Cell. Biol. *17*, 2247–2256.

Yasui, T., Sakakibara-Yada, K., Nishimura, T., Morita, K., Tada, S., Mosialos, G., Kieff, E., and Kikutani, H. (2012). Protein kinase N1, a cell inhibitor of Akt kinase, has a central role in quality control of germinal center formation. Proc. Natl. Acad. Sci. USA *109*, 21022–21027.

Ybot-Gonzalez, P., Savery, D., Gerrelli, D., Signore, M., Mitchell, C.E., Faux, C.H., Greene, N.D., and Copp, A.J. (2007). Convergent extension, planar-cell-polarity signalling and initiation of mouse neural tube closure. Development *134*, 789–799.

Zhao, Z.S., and Manser, E. (2005). PAK and other Rho-associated kinases–effectors with surprisingly diverse mechanisms of regulation. Biochem. J. *386*, 201–214.

Cell Reports
Supplemental Information

Knockout of the PKN Family of Rho Effector Kinases Reveals a Non-redundant Role for PKN2 in Developmental Mesoderm Expansion

Ivan Quétier, Jacqueline J.T. Marshall, Bradley Spencer-Dene, Sylvie Lachmann, Adele Casamassima, Claudio Franco, Sarah Escuin, Joseph T. Worrall, Priththivika Baskaran, Vinothini Rajeeve, Michael Howell, Andrew J. Copp, Gordon Stamp, Ian Rosewell, Pedro Cutillas, Holger Gerhardt, Peter J. Parker, and Angus J.M. Cameron

Figure S1

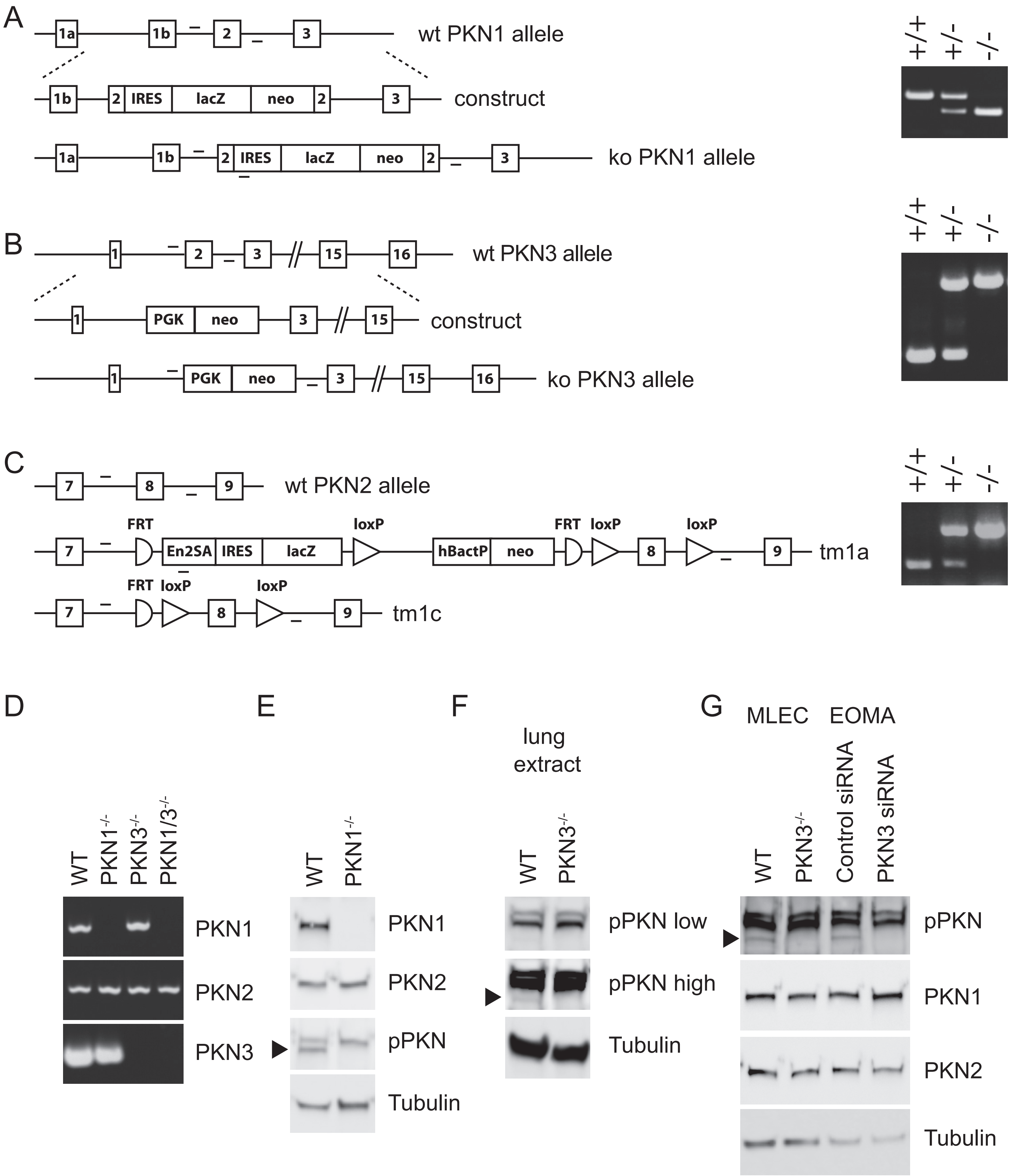
A
1a
1b
2
3
wt PKN1 allele
construct
IRES
lacZ
neo
ko PKN1 allele
+/+
+/-
-/-
B
1
15
16
wt PKN3 allele
PGK
ko PKN3 allele
C
7
8
9
wt PKN2 allele
FRT
loxP
En2SA
hBactP
tm1a
tm1c
D
WT
PKN1-/-
PKN3-/-
PKN1/3-/-
PKN1
PKN2
PKN3
E
pPKN
Tubulin
F
lung extract
pPKN low
pPKN high
G
MLEC
EOMA
Control siRNA
PKN3 siRNA

Figure S1. Generation of PKN1, PKN2 and PKN3 knockouts, related to figure 1. Schematic representation of the wild-type (wt) and targeted alleles for the three mouse PKN genes. For PKN1 (A) and PKN3 (B) a representation of the targeting construct is also provided. The PKN2 tm1a allele is a knockout first allele obtained from the KOMP consortium (C). WT and targeted alleles for each of the three PKN isoforms can be distinguished by PCR (right hand panels). The position of the primers used for PCR are indicated on the schematic and details of the primers and product sizes are provided in supplementary experimental procedures. RT-PCR demonstrating disruption of PKN1 and PKN3 mRNA extracted from mouse lungs (D). Specific primers used are detailed in supplementary experimental procedures. PKN expression in wt and PKN1 knockout (-/-) mouse embryo extracts was assessed by immunoblot (E). PKN3 was undetectable in mouse embryos but could be detected using a phospho-PKN specific antibody in mouse lung extracts (F) and mouse lung endothelial cells (G). To positively identify PKN3, protein was depleted by siRNA from murine EOMA cells. Detection of α-tubulin was used to control for protein loading.

Figure S2

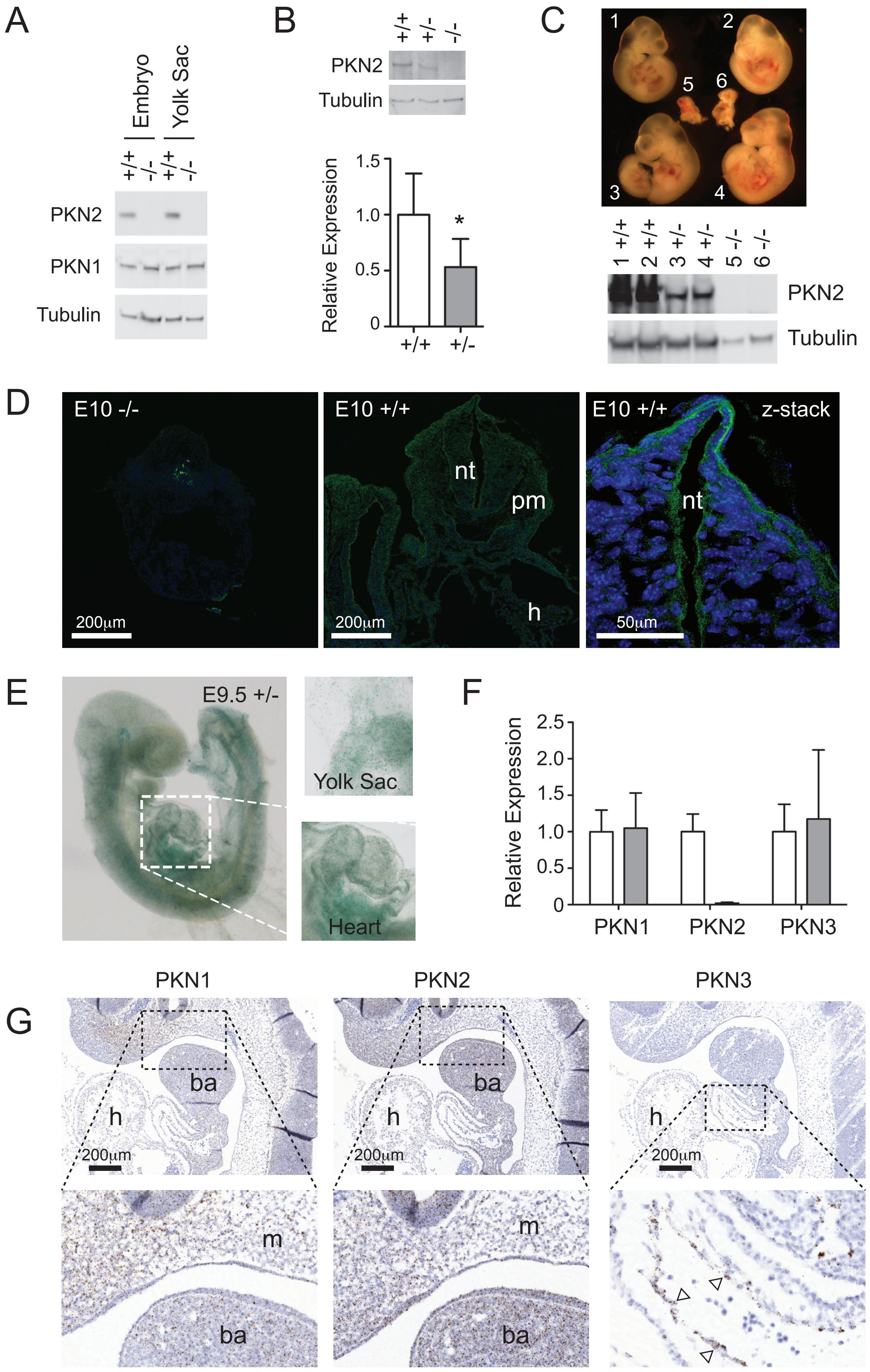

A
Embryo
Yolk Sac
+/+
-/-
+/+
-/-
PKN2
PKN1
Tubulin
B
+/+
+/-
-/-
PKN2
Tubulin
Relative Expression
1.5
1.0
0.5
0
*
+/+
+/-
C
1
2
3
4
5
6
1 +/+
2 +/+
3 +/-
4 +/-
5 -/-
6 -/-
PKN2
Tubulin
D
E10 -/-
200µm
E10 +/+
nt
pm
h
200µm
E10 +/+
z-stack
nt
50µm
E
E9.5 +/-
Yolk Sac
Heart
F
Relative Expression
2.5
2.0
1.5
1.0
0.5
0
PKN1
PKN2
PKN3
G
PKN1
PKN2
PKN3
ba
h
200µm
m
ba

Figure S2. PKN2 is broadly expressed and is required in the embryo proper, related to figure 1. PKN2 protein is expressed in the embryo and yolk sac (A) and is expressed at approximately half wild-type levels in heterozygous embryos (B); error bars represent S.D.; * < P 0.05, n=4). A single tetraploid rescue litter comprising two wild-type, two heterozygous and two knockout embryos (C). Genotypes were determined directly from genomic DNA isolated from the embryos. To assay for PKN2 protein, embryo lysates were subjected to Western blot analysis and α-Tubulin was used as a control for protein loading (C, bottom panels). Frozen section of E10 knockout (-/-) and wt embryos were stained with anti-PKN2 mAb (R&D systems) and an Alexa-488 secondary antibody. DAPI counterstained embryos were visualised by confocal microscopy (D). To visualise apical accumulation of PKN2, z-stack images were compiled as a projection (D, right hand panel). Whole mount x-gal staining reveals broadly ubiquitous expression in heterozygous embryos (E). Expression of PKN isoform mRNA expression was assessed in E8 embryo extracts by qPCR (F). PKN2 disruption is confirmed by loss of PKN2 mRNA. No expression compensation of PKN1 or PKN3 was observed on loss of PKN2. PKN1, PKN2 and PKN3 mRNA expression in E10 embryos was assessed by RNAscope *in situ* hybridisation as described in supplementary experimental procedures (G). Broad expression of PKN1 and PKN2 can be seen in all tissues whereas PKN3 is restricted; open arrow heads indicate high PKN3 expression in the endocardial lining of the bulbus arteriosus. Abbreviations: nt, neural tube; pm, pharyngeal mesoderm; h, heart; ba, branchial arch; m, mesenchyme.

Figure S3

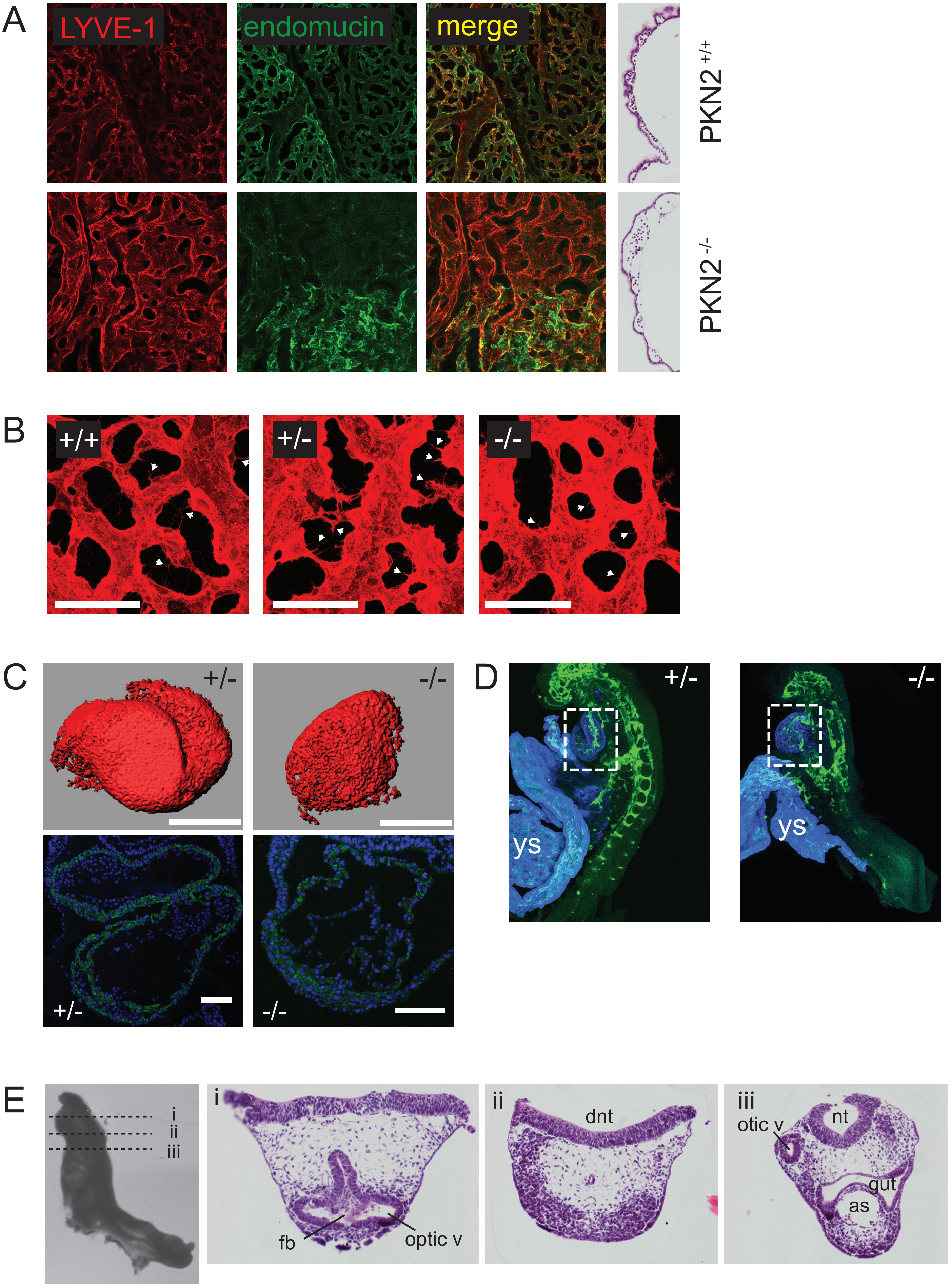
A
LYVE-1
endomucin
merge
PKN2 +/+
PKN2 -/-
B
+/+
+/-
-/-
C
+/-
-/-
+/-
-/-
D
+/-
-/-
ys
ys
E
i
ii
iii
i
fb
optic v
ii
dnt
iii
otic v
nt
gut
as

Figure S3. PKN2 knockout yolk sacs exhibit an immature vascular plexus and heart development is retarded, related to figure 1. Yolk sac endothelial cells were stained with endomucin and anti-LYVE-1 antibodies (A). Yolk sacs from E9.5 embryos were also paraffin embedded and stained with H&E (A, right hand panels). High magnification images of LYVE-1 staining reveal endothelial protrusions, indicated by arrows (B). To examine the delay in cardiac looping, E8.5 embryos were whole mount stained for desmin and 3D reconstructions were processed using Bitplane Imaris software (C, upper panels). Transverse sections were also stained for desmin followed by Alexa-488 secondary to reveal cardiomyocytes (C; lower panels). (D) Whole mount staining of PKN2 heterozygous and knockout embryos and yolk sacs (ys) with desmin (blue - Alexa 647) and endomucin (green – Alexa 488). Desmin stained hearts (boxed) are un-looped in the knockout embryos compared to littermates. Sections were counterstained with DAPI. Histological H&E transverse sections through the head of an E9.5 PKN2 knockout embryo revealed a fully open hind-brain neural tube (E). Abbreviations are: ys, yolk sac; otic v, otic vesicle; optic v, optic vesicle; nt, neural tube; dnt, dorsal neural tube; fb, forebrain; as, aortic sac.

Figure S4

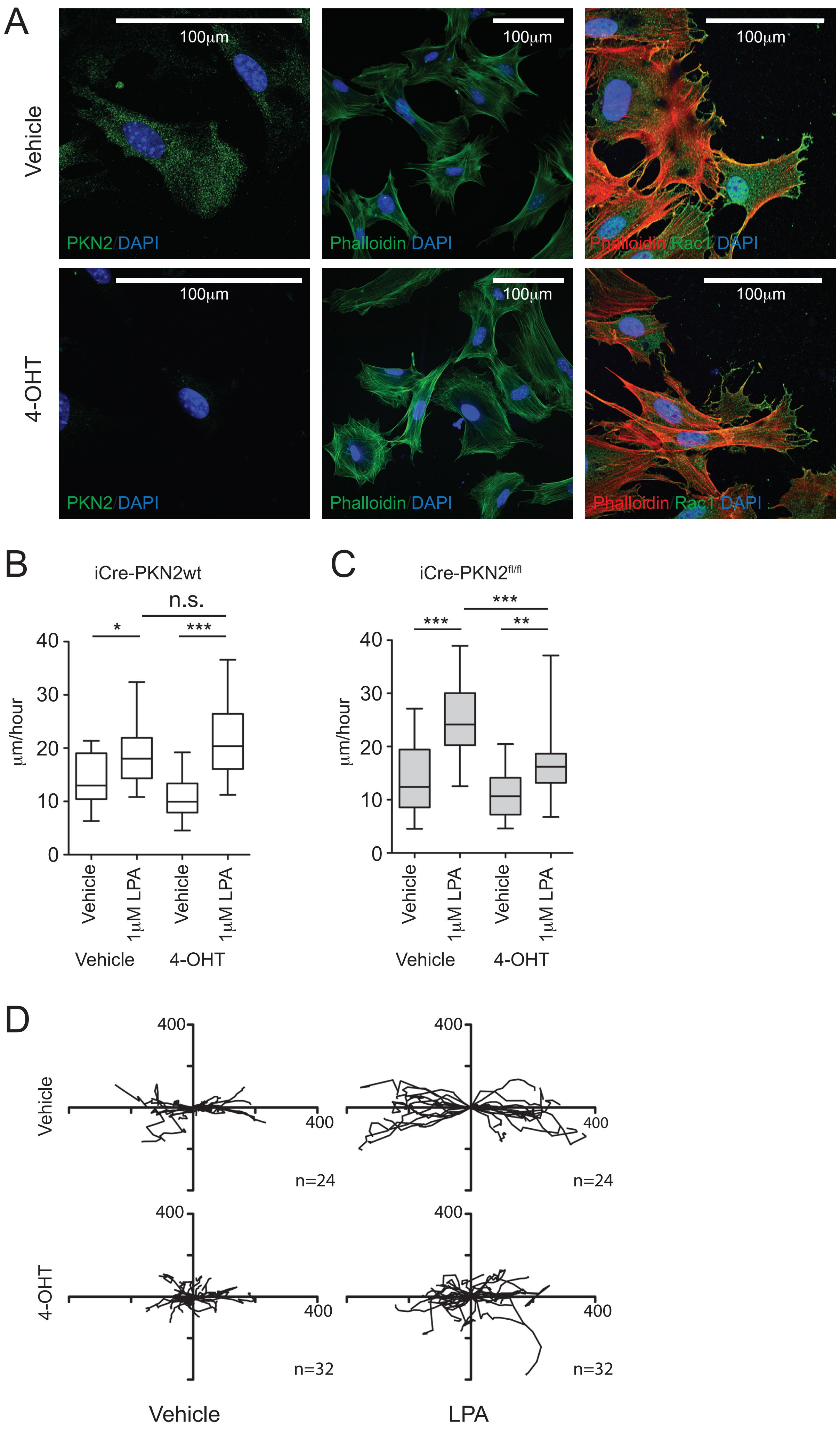
A
Vehicle
4-OHT
100µm
PKN2/DAPI
Phalloidin/DAPI
Phalloidin/Rac1/DAPI
B
iCre-PKN2wt
n.s.
*
***
µm/hour
Vehicle
1µM LPA
Vehicle
4-OHT
C
iCre-PKN2fl/fl
***
**
D
Vehicle
LPA
4-OHT
400
n=24
n=32

Figure S4. Effects of PKN2 deletion on actin organisation and LPA induced migration, related to figure 3. The actin cytoskeleton remains largely unaffected by 4-OHT induced PKN2 deletion (A). MEFs were methanol fixed and stained with anti-PKN2 mAb and alexa-488 labelled secondary, and DAPI, prior to confocal imaging (left panels); 4-OHT induced deletion of PKN2 (left bottom panel). Following PFA fixation, F-actin was stained with FITC-phalloidin (actin) and nuclei with DAPI (A, middle panels). Rac1 and actin are localised at the leading edge of migrating cells in PKN2 expressing or PKN2 deleted cells (A, right panels). Cells were stained with anti-Rac1 mAb and alexa-488 labelled secondary, Alexa-555 labelled phalloidin and DAPI. Single cell tracking reveals that PKN2 deletion supresses LPA induced migration speed of MEFs in scratch wound assays (B-D); 4-OHT induced Cre activity reduces migration of PKN2$^{fl/fl}$ but not PKN2wt cells. Box and whisker plots indicate the average, quartiles and range of individual cell speed; *<P 0.05; **p<0.01;***p<0.001 ANOVA. Single cell tracks from both wound edges are displayed for the iCre-PKN2$^{fl/fl}$ cells (D; axes indicate distance in μm).

Parent Genotype (male and female)	Age	Offspring/ Embryos	Wild-Type	Heterozygous	Knockout/ Homozygous
PKN1 $^{+/-}$	-	91	21	44	26
PKN3 $^{+/-}$	-	168	43	87	38
PKN1 $^{+/-}$ PKN3 $^{+/-}$	-	222	20	190	12
PKN2$^{tm1a\ +/-}$	-	171	81	90***†	0***
PKN2$^{tm1c\ +/fl}$	-	36	11	18	7
PKN2$^{tm1a\ +/-}$	E8.5	30	9	13	8
PKN2$^{tm1a\ +/-}$	E9.5	76	22	34	20
PKN2$^{tm1a\ +/-}$	E10.5	23	6	15	2
PKN2$^{tm1a\ +/-}$	E11.5+	56	15	41	0***

Table S1. Disruption of the three PKN genes in mouse reveals that PKN2 is essential in development, related to figure 1. All mice are on a C57B/6J background. For PKN1 $^{+/-}$ PKN3 $^{+/-}$ double het crosses, wild-type and knockout numbers refer to homozygosity at both alleles; heterozygous numbers indicate heterozygosity at either allele. PKN2^{tm1a} is a knockout first allele (KOMP), which is converted to a floxed (fl) conditional allele by flp recombinase to give pkn2^{tm1c}. †On this background there was a partially penetrant haplo-insufficiency despite adult wt and heterozygous mice being fertile, normal weight and overtly indistinguishable. Statistically significant deviation for expected numbers are indicated by asterisks; ***P < 0.001; χ^2 test.

Parent Genotypes	Age	Offspring/ Embryos	Cre negative		Cre positive	
			PKN2$^{fl/+}$	PKN2$^{fl/fl}$	PKN2$^{fl/+}$	PKN2$^{fl/fl}$
Tie2-Cre$^{+/-}$PKN2$^{fl/+}$ x PKN2$^{fl/fl}$	-	45	8	9	16	12
SM22-Cre$^{+/-}$PKN2$^{fl/+}$ x PKN2$^{fl/fl}$	-	48	48	31	40	7***
SM22-Cre$^{+/-}$PKN2$^{fl/+}$ x PKN2$^{fl/fl}$	E9.5	16	2	5	4	5
	E11.5	19	5[a]	6	13	4[b]
	E13.5	8	3	2	1	2
	E9.5-13.5	52	10	13	18	11
	Expected Fraction:		0.25	0.25	0.25	0.25

Table S2. Conditional PKN2 knockout offspring and embryo numbers, related to figure 2. All mice are on a C57B/6J background. Statistically significant deviation for expected numbers are indicated by asterisks; ***P < 0.001; χ^2 test. [a]one embryo malformed and dead; [b]one embryo small and dead.

Gene name and phosphorylation site identified/Protein name/Description and processes regulated.	Vehicle: 0.5% Serum	4-OHT: 0.5% Serum	Vehicle: 10% Serum	4-OHT: 10% Serum	Fold change in serum (Vehicle/4-OHT)
**Pkn2 pS582**: Deleted following 4-OHT treatment.				**	55.3
**Zc3hc1 pS394**: NIPA; E3 ligase complex component – mitotic entry.				**	17.6
**Anln pS180**: Anillin; structural integrity of cleavage furrow – cytokinesis.		***		***	12.9
**Top2a pT1350**: Topoisomerase 2; chromosome segregation –mitosis.		***		**	11.3
**Tpx2 pS486**: Spindle assembly factor and microtubule nucleation – mitosis.				*	8.5
**Anln pS293**: Anillin; structural integrity of cleavage furrow – cytokinesis.		*		*	7.9
**Tpx2 pS737**: Spindle assembly factor and microtubule nucleation – mitosis.		*		*	6.2
**Ska3 pS154**: Kinetochore complex component – mitsosis.		*		**	5.6
**Pkn2 pS619**: Deleted following 4-OHT treatment.		**		*	5.3
**Rgcc pS97**: Regulator of Cell Cycle; enhances cdk1 activity – cell cycle.		*		**	4.9
**Arpp19 pS23**: cAMP regulated pp19; mitotic PP2A inhibitor - mitosis		***		**	4.7
**Anln pS180**: Anillin; structural integrity of cleavage furrow – cytokinesis.		**		*	4.3
**Lig1 pS49**: DNA ligase 1; seals nicks during DNA replication – S-phase.		**		**	4.2
**Cep55 pS428**: Centrosomal protein required for mitotic exit - mitosis		*		***	3.8
**Lig1 pT77**: DNA ligase 1; seals nicks during DNA replication – S-phase.					3.6
**Ercc6l pS1021**: Spindle checkpoint helicase binding Mad2 – mitosis.		**		*	3.4
**Lig1 pS51**: DNA ligase 1; seals nicks during DNA replication – S-phase.		**		**	3.2
**Lig1 pS188**: DNA ligase 1; seals nicks during DNA replication – S-phase.		*		**	2.8
**Mcm3 pS672**: DNA replication licencing factor – replicative helicase – S-phase.				***	2.6
**Ncapd2 pS1320**: Condensin complex subunit 1 – mitosis.		**		*	2.6

**Mcm3 pS672**: DNA replication licencing factor – replicative helicase – S-phase		*		**	2.5
**Mcm2 pS41**: DNA replication licencing factor – replicative helicase – S-phase				*	1.7
**Mcm2 pS21**: DNA replication licencing factor – replicative helicase – S-phase		*		*	1.7
**Hmga2 pS101 pS104**: Transcriptional regulator - cell cycle		*			0.8
**Pard3 pS174**: Cell polarisation complex protein – cell cycle.		*			0.7
**Pard3 pS221**: Cell polarisation complex protein – cell cycle.				*	0.6
**Mcm2 pT39 pS41**: DNA replication licencing factor; helicase – S-phase.		*			0.5
**Hmga2 pT99 pS100 pS101**: Transcriptional regulator - cell cycle				**	0.4
**Arhgef2 pS955**: Rho GTPase exchange factor regulating division – mitosis.				**	0.1

-5	-4	-3	-2	-1	0	1	2	3	4	5

Key: Relative Fold Change

Table S3. Summary of cell cycle and mitosis associated phosphopeptides significantly modulated by PKN2 deletion, related to figure 3. PKN2 iMEFs were treated with 4-OHT or Vehicle, cultured for 48 hours to allow PKN2 loss, serum starved overnight and, where indicated, re-stimulated with serum for 30 minutes prior to cell lysis. Phosphopeptides were analysed by LC-MS/MS across 4 biological replicates. Hits are compiled from combined cell division (GO:0051301), mitosis (GO:0007067) and cell cycle (GO:0007049) ontologies. Only peptides significantly modulated by PKN2 loss are included and hits are ordered according to average fold change after 10% serum re-stimulation. Colours represent phosphopeptide abundance relative to the sum of the four conditions. Asterisks indicate significant differences between Vehicle and 4-OHT treatments under either serum free or serum stimulated conditions as indicated; Benjamini & Hochberg corrected p-values: * <0.05; **<0.01; ***<0.001.

Gene name and phosphorylation site identified/Protein name/Description and processes regulated.	Vehicle: 0.5% Serum	4-OHT: 0.5% Serum	Vehicle: 10% Serum	4-OHT: 10% Serum	Fold change in serum (Vehicle/4-OHT)
**Anln pS180:** Anillin; actin binding protein that regulates cytoskeletal dynamics.		***		***	12.9
**Anln pS293:** Anillin; actin binding protein that regulates cytoskeletal dynamics.		*		*	7.9
**Myo18a pS83:** Myosin 18a; modulates lamellar actomyosin - cell protrusion and migration		**		*	3
**Ppp1r18 pS212:** Protein Phosphatase 1, Regulatory Subunit 18; targets PP1 to F-actin				*	2.5
**Map1b pS1293 pS1307:** Microtubule-Associated Protein 1B; microtubule assembly		**		**	2.4
**Nes pS169:** Nestin; intermediate filament protein - cytoskeleton		**		*	2.4
**Flna pS1084:** Filamin A; actin-binding protein, links actin to membrane		*		***	2.3
**Cgn pS131:** Cingulin; *actin binding* – cytoskeletal dynamics		*			2.2
**Nes pS731:** Nestin; intermediate filament protein – cytoskeletal dynamics		*		***	2
**Nes pS688:** Nestin; intermediate filament protein – cytoskeletal dynamics		**		**	2
**Nes pS623:** Nestin; intermediate filament protein – cytoskeletal dynamics		*		**	1.8
**Zyx pS336:** Zyxin; concentrates at focal adhesions – cytoskeletal organization				*	1.6
**Palld pS782:** Palladin; component of actin-containing microfilaments - cytoskeletal dynamics				**	1.6
**Fhod1 pS502:** Formin Homology 2 Domain Containing 1; Role in assembly of F-actin structures				**	0.9
**Svil pS960:** Supervillin; links actin cytoskeleton and the membrane – cytoskeletal dynamics				*	0.7
**Epb41l1 pS782:** Erythrocyte Membrane Protein Band 4.1-Like 1 – actin binding		*		**	0.7
**Ssh3 pS639:** Slingshot Protein Phosphatase 3 – actin filament dynamics		**		***	0.4
**Ppp1r18 pS224**: Protein Phosphatase 1, Regulatory Subunit 18; targets PP1 to F-actin				*	0.3
**Myo1h pT356 pS364 pS365:** Myosin-1H; actin-based motor molecules with ATPase activity				**	0.2
**Arhgef2 pS955:** Rho/Rac Guanine Nucleotide Exchange Factor (GEF) 2; Activates Rho-GTPases				**	0.1

-5	-4	-3	-2	-1	0	1	2	3	4	5

Key: Relative Fold Change

Table S4. Summary of cytoskeleton, intermediate filament and focal adhesion associated phosphopeptides significantly modulated by PKN2 deletion, related to figure 3. PKN2 iMEFs were treated with 4-OHT or Vehicle, cultured for 48 hours to allow PKN2 loss, serum starved overnight and, where indicated, re-stimulated with serum for 30 minutes prior to cell lysis. Phosphopeptides were analysed by LC-MS/MS across 4 biological replicates. Hits are compiled from combined actin cytoskeleton (GO:0031523; GO:0003779), focal adhesion (GO:0005925), microtubule (GO:0001578) and intermediate filament (GO:0005882) ontologies. Only peptides significantly modulated by PKN2 loss are included and hits are ordered according to average fold change after 10% serum re-stimulation. Colours represent phosphopeptide abundance relative to the sum of the four conditions. Asterisks indicate significant differences between Vehicle and 4-OHT treatments under either serum free or serum stimulated conditions as indicated; Benjamini & Hochberg corrected p-values: * <0.05; **<0.01; ***<0.001.

SUPPLEMENTARY EXPERIMENTAL PROCEDURES

Mice

To generate PKN1 null mice, a HindIII/SpeI fragment of the PKN1 genomic sequence was cloned and an IRES/β-Galactosidase/neomycin cassette was introduced into a SmaI site in the second exon; PKN1 has two alternate transcripts which differ in their first exon usage (termed 1a and 1b) and the disrupted exon is the first exon common to both transcripts. PKN1 targeted GK129 Embryonic Stem clones (PKN1$^{+/-}$) were isolated and injected into C57BL/6 blastocysts to generate chimeras. Several chimeras gave germ line transmissions, which were used to establish the homozygous PKN1-deficient mouse line. Mice were backcrossed onto C57BL/6 for a minimum of 6 generations. Genotypic characterization of recombinant ES cells and adult mice was confirmed by Southern blot analysis of KpnI-digested genomic DNA. Mice were genotyped from ear clip genomic DNA with PKN1 specific sense (PKN1-F; 5'-AGAGCACTATGTGTTTTTAGCTGCTGAGCC-3') and antisense (PKN1-R; 5'-TGTCCTTAGGTATCTGGTGACAGTGTGGG-3') primers and an IRES cassette antisense (IRES-R; 5'-GCTAGACTAGTCTAGCTAGAGCGGCC-3') to generate a 900bp fragment for the wt locus (PKN1-F with PKN1-R) and a 450bp fragment for the PKN1 ko locus (PKN1-F with IRES-R).

To generate PKN3 null mice, a fragment of the PKN3 genomic sequence was subcloned from a BAC clone (BACPAC RP24-364D12) into the NotI and XhoI sites of pFlrt (Ralf Adams); the PKN3 fragment was bounded by the forward cloning primer, SHA-NotI-F (5'-ATAAGAATGCGGCCGCGTTACCGTGGAATATGCCACCG-3') and a naturally occurring XhoI site situated between exon 15 and exon 16 of PKN3. The coding sequence of Exon 2 was replaced by a PGK-neo cassette, to generate the PKN3 targeting construct using the Quick and Easy Conditional Knockout Kit (Gene Bridges) according to the manufacturers instructions. PKN3 targeted Primogenix B6.1 (C57Bl/6N) ES cells were identified by PCR

screening and injected into C57Bl/6 blastocysts to generate chimeras and germ line transmission. Correct integration was confirmed by multiple diagnostic PCR of genomic DNA using primers flanking both the short and long homology arms of the PKN3 knockout construct. Mice were genotyped from earclip genomic DNA using PKN3 specific sense (PKN3-F3; 5'- CCAGAAGGCACTGGGCGAAC -3) and antisense (PKN3-R1; 5'- CAACACAAGGCTAGAGTTCA -3) primers to generate a 540bp fragment for the wt locus and a 2kb fragment for the ko locus.

To generate PKN2 null mice targeted ES cells were obtained from the KOMP Repository (www.komp.org: Project ID66263 - pkn2 MGI:109211). Two independent knockout first ES cell clones underwent germline transmission: clone G05 (allele: Pkn2$^{tm1a(KOMP)Wtsi}$) and clone G08 (allele: Pkn2$^{tm1e(KOMP)Wtsi}$). Clone G08 has lost the 3' loxP site necessary to allow generation of a conditional mouse through FLP/FRT recombination so provides a null allele only. Mice were genotyped from ear clip genomic DNA with PKN2 specific sense (PKN2-F2; 5'- GGTTTGGTGACCAGTAAAAACTG -3') and antisense (PKN2-R1; 5'- GGAATACAGAACAAGGCAAAAGC -3') primers and an insert specific antisense primer (En2R; 5'-CCAACTGACCTTGGGCAAGAACAT -3') to generate a 1089bp fragment for the wt locus and a 395bp fragment for the PKN2 ko locus. To convert the PKN2 to a conditional allele, PKN2 heterozygous mice were crossed with a Flp deleter mouse (Tg(CAG-Flpo)1Afst; background C57Bl/6N); to genotype, sense primer PKN2-F2 was used with a second gene specific antisense primer (PKN2-R2; 5'- CTGAAGACACTTTGAAAAGGATG -3') to generate 489bp and 635bp products for the wt and conditional alleles respectively.

Tetraploid embryos for rescue experiments were generated by fusing 2 cell embryos (B6CBAF1 x F1) on the Cell-fusion instrument, CF-150B available from BLS Ltd., Hungary. Successfully fused embryos were held in culture and allowed to develop to the 8 cell

stage. 8 cell stage tetraploid embryos and PKN2 embryos (stemming from heterozygote x heterozygote matings) were rendered zona free with Acid Tyrodes solution before aggregation of 1-2 tetraploid embryos with each PKN2 embryo according to standard protocols (Nagy and Rossant, 1993). Aggregated embryos were transferred into pesudopregnant surrogate mice.

Tie2-Cre (Koni et al., 2001) and SM22α-Cre mice (Lepore et al., 2005) on a C57BL/6 background were kindly provided by Taija Makinen and Ralf Adams. Rosa26CreERT2 mice (Gt(ROSA)26Sortm9(cre/ESR1)Arte) are from Taconic biosciences. Cre positive mice were identified using Cre sense (Cre-F; 5'- ACTATCCAGCAACATTTGGGCC -3') and antisense (Cre-R; 5'- CCGGCAAAACAGGTAATTCGG -3') primers to generate a 450bp fragment. For *in-vivo* bromodeoxyuridine (BrdU) labeling SM22α-Cre mice were injected with 2mg BrdU i.p. 2 hours prior to sacrifice.

Antibodies and Staining

Antibodies used were: PRK1 mAb (BD biosciences), PKN2 mAb (Clone 509105; N-terminal epitope amino acids 18-207; R&D systems), PKN2 mAb (Clone 1D1; C-terminal epitope amino acids 555-718, Abcam), Tubulin mAb (Sigma; Clone B-5-1-2), LYVE-1 (Reliatech GmbH), PECAM-1 rat mAb (BD Biosciences), Desmin mAb (Dako), BrdU mAb (Dako), caspase 3 (R&D systems), Annexin V (Biolegend), phospho-Histone H3 S10 (EMD Millipore), anti-endomucin rat mAb (Santa Cruz), GAPDH (Santa Cruz), Cyclin A (Santa Cruz), Cyclin D1 (Spring Bioscience), PCNA (Oncogene Research Products), Mcm2 (Bethyl) and phospho-specific polyclonal antibodies (PRK1/2 T744, ERK, Akt, p70S6K, cdk1 Y15, eIF4E S209; Cell Signaling Technology). Secondary HRP (Amersham) and Alexa 488/555/647 (Invitrogen) antibodies were used as appropriate. For immunostaining, embryos were fixed overnight in 10% neutral buffered formalin, embedded in paraffin; 4μm sections were treated with

sodium citrate (pH 6.0) buffer prior to immunostaining. HRP-secondary antibodies were developed with 3,3'-diaminobenzidine (DAB). Fluorescently stained sections were incubated for 30 minutes in 0.1 % Sudan Black in 70% EtOH and mounted in Hardset mount with DAPI (Vector). For β-galactosidase staining, embryos were fixed in 0.4% paraformaldehyde and stained using X-gal according to standard protocols. For standard histological analysis 4μm sections were stained with hematoxylin and eosin (H&E). For frozen sections, embryos were embedded and frozen in OCT. 8mm sections were dried at room temperature for 30 min, permeabilized with 0.1% Triton-x100 for 10 min, blocked in PBS-BSA 2% and stained with anti-PKN2mAb (R&D systems), alexa488 secondary and DAPI. Images were acquired on a Nikon Eclipse 90i upright microscope or Zeiss LSM confocal and processed using NIS-elements software, Zen and Adobe Photoshop CS5.

For whole-mount staining, embryos were dissected, fixed in 4% PFA, permeabilised with 0.5% triton X100, blocked with PBSMT (PBS supplemented with 3% milk and 0.1% Tween 20) and stained overnight with appropriate primary antibodies. For PECAM stains embryos were then washed and incubated overnight with a peroxidase-conjugated goat anti-rat IgG (Pierce), washed and developed with Substrate G (Vector) and cleared in glycerol, based on published protocols (Adams et al., 1999). Images were acquired on a Nikon Eclipse 90i upright microscope and processed using NIS-elements software. For fluorescent staining appropriate alexa 488/555/647 were used as indicated and confocal images were acquired on a Zeiss LSM 710/780 inverted microscope and processed using ImageJ, Zen and Adobe Photoshop; 3D reconstructions of desmin stained hearts were processed using Bitplane Imaris software.

***In-situ* hybridization**

For PKN1, PKN2 and PKN3 in-situ staining, an RNAscope 2.0 FFPE assay kit was used

(Advanced Cell Diagnostics) according to the manufacturers instructions. The sections were incubated with RNAscope custom oligonucleotide probes: Mouse PKN1, accession no. NM_001199593.1, target region 384-1432; mouse PKN2, accession no. NM_178654.4, target region 634-1628; mouse PKN3, accession no. NM_153805.1, target region 401-1352. Probes were incubated for 2 hours at 40°C and signals developed according to manufacturers instructions using the RNAscope 2.0 HD Assay- BROWN system and hematoxylin counterstain. Whole-mount *in-situ* staining of ErbB3 was carried out as elsewhere described (Pryor et al., 2014).

Cells and treatments

For siRNA depletion of mouse PKN3, EOMA cells were transfected using 10 nM Dharmacon ON-TARGET plus siRNA smartpool (4 duplexes: #1: 5'- GCACCGACUUCGAGUAGAG -3'; #2: 5'- GGAAGGAAUUGGCUUUGGU -3'; #3: 5'- GGACACUUGUGAAACCUAU -3'; #4: 5'- GUACAAAGGAACAGGGAAA -3') using HiPerfect (Qiagen). Cells were harvested after 48 hours and examined by immunoblot. Mouse lung endothelial cells (MLEC) were isolated and cultured as described by Reynolds et al (Reynolds and Hodivala-Dilke, 2006). For mouse embryonic fibroblasts (MEF), embryos were decapitated and foetal liver was removed prior to trypsin digestion and serial passage in DMEM with 10% FBS. Lines were immortalised using a 3T3 protocol of serial passage and subsequent senescence escape. ES cells were isolated from blastocysts collected from heterozygous PKN2$^{fl/+}$ RosaCreERT crosses to enable inducible knockout of PKN2 in culture. Cells were derived as described (Bryja et al., 2006) with the addition of a MEK inhibitor (PD0325901) for the initial stages to improve plating efficiency. After 4 passages the cells were grown in standard, serum containing ES cell media. Male cell lines with a good chromosome count were selected. Induction of Cre in RosaCreERT cells was achieved through incubation with 4-hydroxytamoxifen (4-OHT;

typically 100nM) for 1 hour prior to media replacement.

Immunoblotting

Lysates were resolved by SDS page and transferred to PVDF or nitrocellulose membranes. Immunoblots were blocked in TBST (TBS containing 0.1% Tween 20) with 3% BSA and probed with primary antibodies as indicated. Following incubation with appropriate HRP conjugated secondary antibodies (Amersham), bands were visualised by ECL using an Image Quant digital imaging system (GE healthcare) or X-ray film.

Cell Growth and viability

Cell growth was assessed by cell counting, MTT (Methylthiazolyldiphenyl-tetrazolium), and FACs analysis. For MTT assays, cell grown in 96 well plates were incubated with 1mM MTT (Sigma) for 1 hour prior to DMSO solubilisation of formazan product and 550nm absorbance measurement (Tecan). For cell colony staining, monolayers were fixed with 4%PFA and incubated with 0.1% Crystal Violet for 30 minutes.

Cell cycle and apoptosis

For cell cycle and subG1 analysis, cells were fixed in ice cold 70% ethanol, washed with PBS and RNAse A treated prior to staining with propidium iodide. For 5-Bromo-2´-Deoxyuridine (BrdU) analysis, cells were incubated in culture with 10μM BrdU prior to ethanol fixation, acid treatment (2M HCl) and anti-BrdU mAb followed by alexa-488 secondary staining. Mitotic nuclei were stained with anti-phospho-Histone H3 (S10) antibody and alexa-488 secondary. For BrdU and mitotic assays, cell cycle was simultaneously assessed as described above. To measure apoptosis, cells were stained with alexa-647 conjugated anti-annexin V antibody (Biolegend) and propidium iodide in appropriate buffer (10 mM HEPES, pH 7.4; 140 mM NaCl; 2,5 mM $CaCl_2$). Data were acquired on an LSRII fortessa (BD biosciences)

and analysed using FlowJo.

Cell Motility

For migration analysis, confluent cell monolayers were scratch wounded in 96 well format using a 96-well Wound–maker (Essen). Wound closure was followed by time-lapse video capture on an Essen IncuCyte. Individual cells were tracked using the Manual Tracking plugin for ImageJ. A combined minimum of thirty, non-mitotic cells migrating from opposing edges of the scratch were tracked from three experimental repeats for each condition.

RT-PCR

RNA was isolated from mouse lungs and embryos using the RNeasy (Qiagen) RNA isolation system and converted to cDNA using TaqMan (Applied Biosystems) reverse transcription reagents. Specific primers spanning intron exon boundaries for PKN1 and PKN3 were used to assay disruption of transcripts in PKN1 and PKN3 knockout mice. PKN2 was used as an internal control. Specific primers used were: PKN1, sense 5'- GCGACGCCGTACAGAGTGA -3'and antisense 5'- CTGCAAGGGATTGGGTAGCA -3' (336bp), PKN2; sense 5'- AGCGGCATGGCATGTGTCTCT -3'and antisense 5'- TGCGTGCGTCAACGACTGGC -3' (303bp) and PKN3; sense 5'- GAGCCCCCAAGGATGAGAAA -3'and antisense 5'- AGTGTCCCTGTCAAGGCAATAG -3' (790bp). For real-time quantitative assessment of PKN1, PKN2 and PKN3, cDNA was amplified with Sybr Green PCR Master Mix (Applied Biosystems). Data were normalized using the DeltaDeltaCt method with GAPDH as the housekeeping gene, and expressed as the relative mRNA level compared to the control. Primers used were: PKN1, sense 5'-ATCCGGCACACTGGAGACA-3' and antisense 5'- TGGTGGATTCATGGGTTGGAG -3'; PKN2, sense 5'- CTGTGCCTGCAACAGTGC -3' and antisense 5'- CTGTAGAGTCACTGGCCGGA-3', PKN3, sense 5'-CTTGGGAACCCCCAACCTTT-3' and antisense

5’-CAGATCTTTGCAGCCCAGGA-3’ and GAPDH, 5’-GAAGCAGGCATCTGAGGGCC-3’and 5’-AGGCCATGTAGGCCATGAGG-3’.

Quantitative Phosphoproteomics

Following sample extraction and trypsin digestion of control and treated samples, TiO_2 chromatography was used to enrich phosphopeptides(Montoya et al., 2011). Identification and quantification of these phosphopeptides across quadruplicate samples was performed by LC-MS/MS as described elsewhere(Casado and Cutillas, 2011; Rajeeve et al., 2014). Statistical significance of phosphorylation site changes across conditions is assessed and sites are selected with a Benjamini & Hochberg adjusted p-value <0.05. Kinase substrate enrichment analysis (KSEA) was used to infer kinase activity from the phosphoproteomics data(Casado et al., 2013). Ontologies and pathways enriched in the phosphoproteomics data was investigated my matching to existing repositories(Ashburner et al., 2000; Schaefer et al., 2009).

REFERENCES

Adams, R.H., Wilkinson, G.A., Weiss, C., Diella, F., Gale, N.W., Deutsch, U., Risau, W., and Klein, R. (1999). Roles of ephrinB ligands and EphB receptors in cardiovascular development: demarcation of arterial/venous domains, vascular morphogenesis, and sprouting angiogenesis. Genes Dev *13*, 295-306.

Ashburner, M., Ball, C.A., Blake, J.A., Botstein, D., Butler, H., Cherry, J.M., Davis, A.P., Dolinski, K., Dwight, S.S., Eppig, J.T.*, et al.* (2000). Gene ontology: tool for the unification of biology. The Gene Ontology Consortium. Nature genetics *25*, 25-29.

Bryja, V., Bonilla, S., Cajanek, L., Parish, C.L., Schwartz, C.M., Luo, Y., Rao, M.S., and Arenas, E. (2006). An efficient method for the derivation of mouse embryonic stem cells. Stem cells *24*, 844-849.

Casado, P., and Cutillas, P.R. (2011). A self-validating quantitative mass spectrometry method for assessing the accuracy of high-content phosphoproteomic experiments. Molecular & cellular proteomics : MCP *10*, M110 003079.

Casado, P., Rodriguez-Prados, J.C., Cosulich, S.C., Guichard, S., Vanhaesebroeck, B., Joel, S., and Cutillas, P.R. (2013). Kinase-substrate enrichment analysis provides insights into the heterogeneity of signaling pathway activation in leukemia cells. Science signaling *6*, rs6.

Koni, P.A., Joshi, S.K., Temann, U.A., Olson, D., Burkly, L., and Flavell, R.A. (2001). Conditional vascular cell adhesion molecule 1 deletion in mice: impaired lymphocyte migration to bone marrow. The Journal of experimental medicine *193*, 741-754.

Lepore, J.J., Cheng, L., Min Lu, M., Mericko, P.A., Morrisey, E.E., and Parmacek, M.S. (2005). High-efficiency somatic mutagenesis in smooth muscle cells and cardiac myocytes in SM22alpha-Cre transgenic mice. Genesis *41*, 179-184.

Montoya, A., Beltran, L., Casado, P., Rodriguez-Prados, J.C., and Cutillas, P.R. (2011). Characterization of a TiO(2) enrichment method for label-free quantitative phosphoproteomics. Methods *54*, 370-378.

Nagy, A., and Rossant, J. (1993). Production of completely ES cell-derived fetuses. In Gene Targeting: A practical approach, A.L. Joyner, ed. (Oxford University Press), pp. 143-179.

Pryor, S.E., Massa, V., Savery, D., Andre, P., Yang, Y., Greene, N.D., and Copp, A.J. (2014). Vangl-dependent planar cell polarity signalling is not required for neural crest migration in mammals. Development *141*, 3153-3158.

Rajeeve, V., Vendrell, I., Wilkes, E., Torbett, N., and Cutillas, P.R. (2014). Cross-species Proteomics Reveals Specific Modulation of Signaling in Cancer and Stromal Cells by Phosphoinositide 3-kinase (PI3K) Inhibitors. Molecular & cellular proteomics : MCP *13*, 1457-1470.

Reynolds, L.E., and Hodivala-Dilke, K.M. (2006). Primary mouse endothelial cell culture for assays of angiogenesis. Methods in molecular medicine *120*, 503-509.

Schaefer, C.F., Anthony, K., Krupa, S., Buchoff, J., Day, M., Hannay, T., and Buetow, K.H. (2009). PID: the Pathway Interaction Database. Nucleic acids research *37*, D674-679.
